# Supplementary material for: HuD Is a Neural Translation Enhancer Acting on mTORC1-Responsive Genes and Counteracted by the Y3 Small Non-coding RNA
Source: Mol Cell. 2018 Jul 19;71(2):256–270.e10. doi: 10.1016/j.molcel.2018.06.032 (PMC6060611; doi:10.1016/j.molcel.2018.06.032)
Supplement: Document S1. Figures S1–S7 and Table S3 [file mmc1.pdf]

Molecular Cell, Volume *71*

Supplemental Information

HuD Is a Neural Translation Enhancer Acting on mTORC1-Responsive Genes and Counteracted by the Y3 Small Non-coding RNA

Toma Tebaldi, Paola Zuccotti, Daniele Peroni, Marcel Köhn, Lisa Gasperini, Valentina Potrich, Veronica Bonazza, Tatiana Dudnakova, Annalisa Rossi, Guido Sanguinetti, Luciano Conti, Paolo Macchi, Vito D'Agostino, Gabriella Viero, David Tollervey, Stefan Hüttelmaier, and Alessandro Quattrone

Supplementary Figures

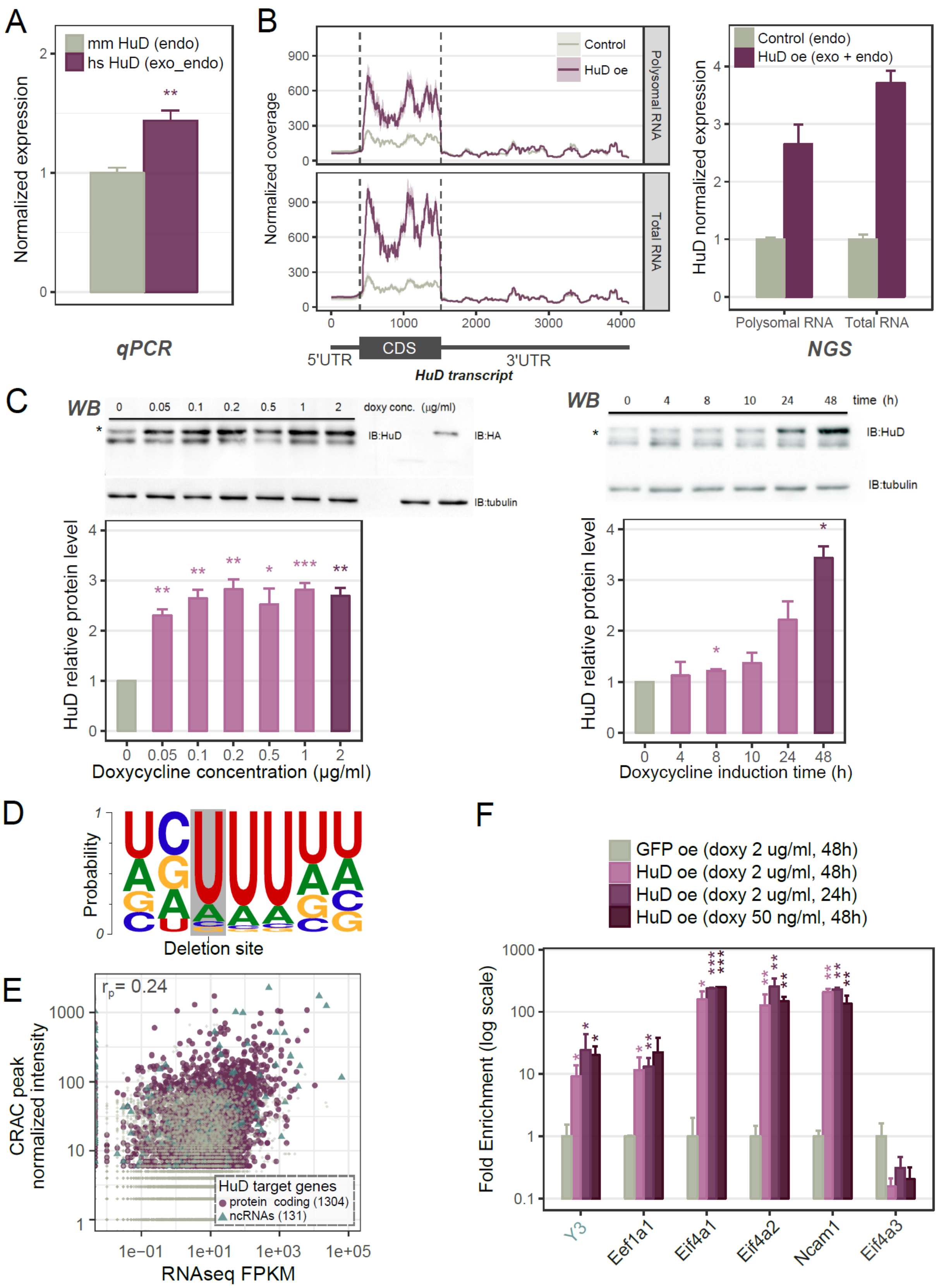

Figure S1. Related to Figure 1.

(A-C) Quantification of exogenous vs endogenous HuD expression in NSC-34 cells upon expression induction (doxy 2ug/ml, 48h).

(A) Quantitative RT-qPCR on human and murine HuD, monitoring endogenous and total HuD expression levels, respectively.

(B) Quantification of HuD exogenous/endogenous levels based on POL-seq and RNA-seq data, comparing the FPKM value of HuD 3'UTR, present only in the endogenous gene, and the FPKM of HuD coding sequence, present in both the endogenous and the exogenous genes.

(C) Quantification of HuD exogenous/endogenous protein expression levels by western blotting. NSC-34 cells were treated with different doses of doxycycline for 48h (upper panel) or with 2 µg/ml of doxycycline for different time points (lower panel). The levels of HuD and loading control α-tubulin were assessed; an HA antibody was used on the same gel to identify the exogenous HuD protein (*).

(D) Logo representation of HuD seed PWM (Position Weighted Matrix) determined from unique CRAC cross-linking locations with combined Fisher p-value < 0.05.

(E) Scatterplot displaying RNA-Seq transcript expression levels (FPKM) and normalized CRAC peak intensity values. The highest peak for each transcript is considered. HuD RNA targets are highlighted and annotated with their gene type.

(F) RIP analysis of HuD-interacting mRNAs encoding (Eef1a1, Eif4a1, Eif4a2 and Y3) under different doses (50 ng/ml or 2 µg/ml) or for different time (24 or 48h) of doxycycline treatment. Ncam1 and Eif4a3 were used a positive and negative control, respectively; data were normalized to Gapdh mRNA levels and expressed over control cells overexpressing His-HA-tagged GFP.

Data are represented as mean ± SEM. t-test $*p < 0.05$, $**p < 0.01$ and $***p < 0.001$.

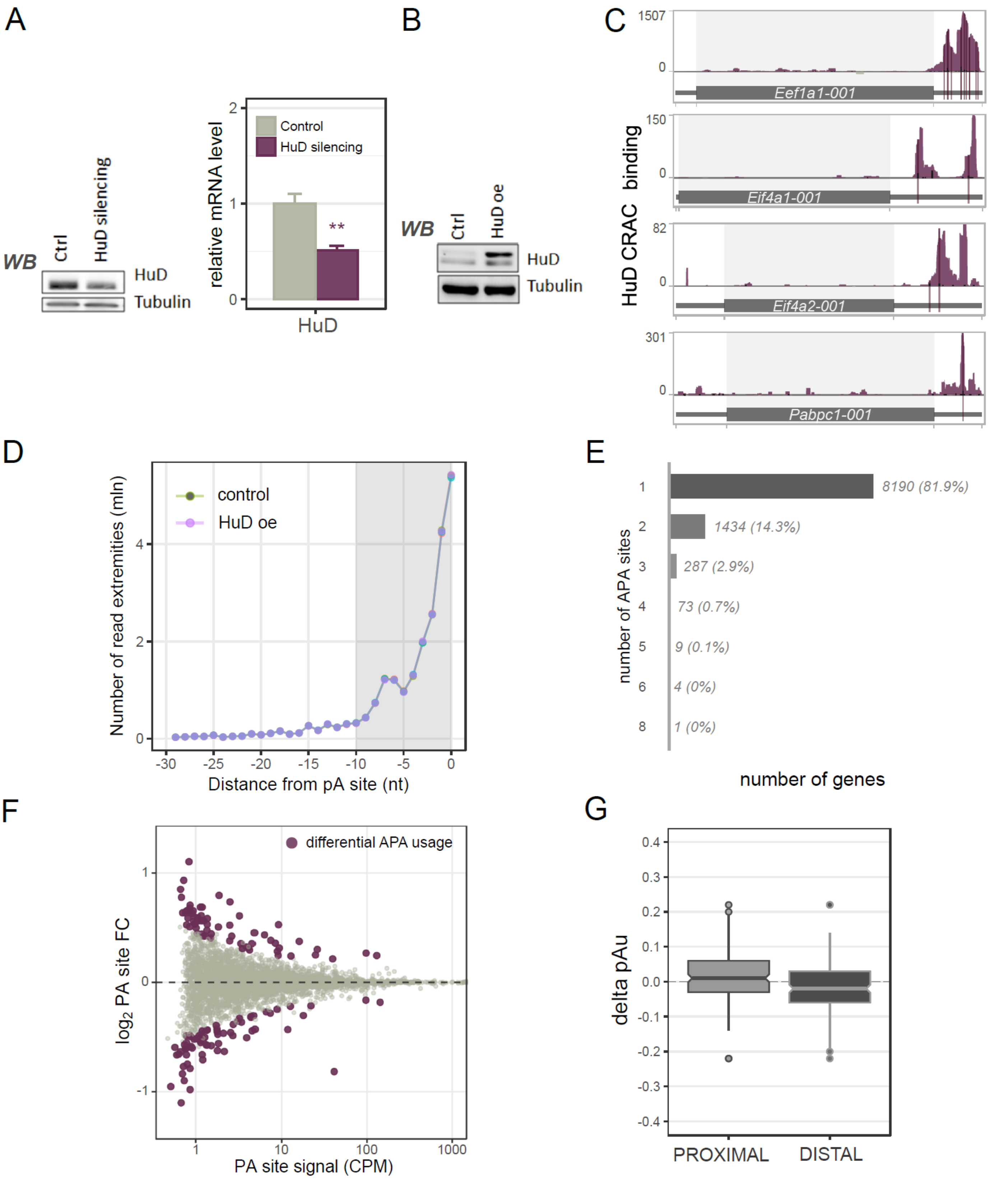

Figure S2. Related to Figure 2.

(A) HuD protein levels (left) and HuD mRNA levels (right) in NSC34 cells after transfection with Ctrl siRNA or HuD siRNA. Gapdh was used as reference gene for qPCR, while tubulin as a reference gene for western

blotting. Data were normalized to tubulin protein levels. Data are represented as mean ± SEM. t-test $**p < 0.01$.

(B) Western blot showing the overexpression of HuD protein upon doxycycline treatment (doxy 2ug/ml, 48h). Results were normalized to tubulin.

(C) Positional binding maps for three mTOR responsive HuD targets: Eef1a1, Eif4a1, Eif4a2 and Pabpc1.

(D-G): Transcriptome-wide alternative polyadenylation (APA) changes upon HuD overexpression in NSC-34 cells. Polyadenylation sites were identified and quantified by Lexogen 3'end mRNA sequencing.

(D) Polyadenylation site usage (pAu) values were determined counting the number of reads starting within 10 nucleotides from known polyadenylation sites.

(E) Distribution of the number of polyadenylation sites identified for each gene.

(F) Identification of differentially used polyadenylation sites upon HuD overexpression.

(G) Distribution of polyadenylation usage differences for proximal versus distal polyadenylation sites upon HuD overexpression.

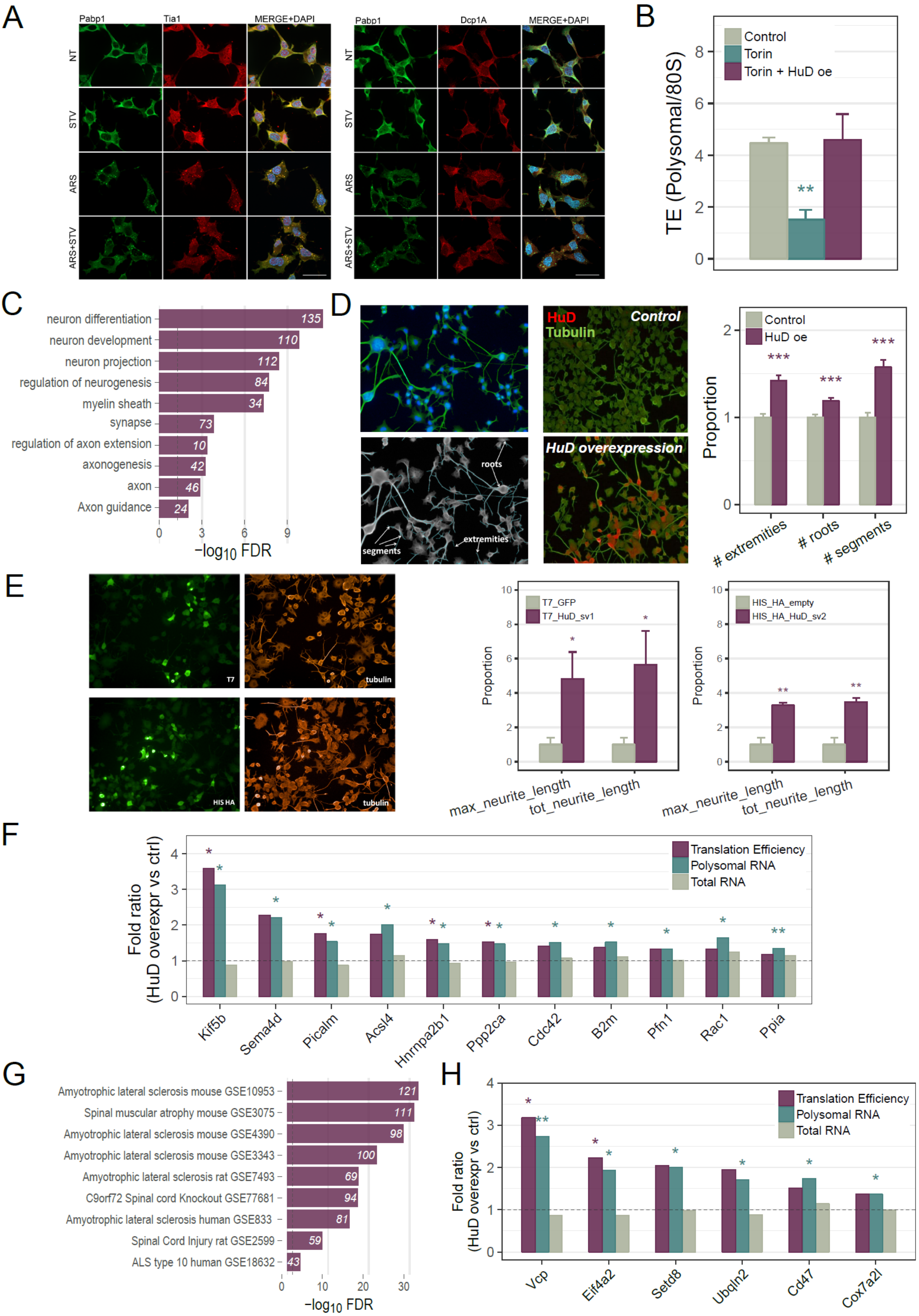
A
Pabp1
Tia1
MERGE+DAPI
Pabp1
Dcp1A
MERGE+DAPI
NT
STV
ARS
ARS+STV
B
Control
Torin
Torin + HuD oe
TE (Polysomal/80S)
**
0
2
4
6
8
C
neuron differentiation
neuron development
neuron projection
regulation of neurogenesis
myelin sheath
synapse
regulation of axon extension
axonogenesis
axon
Axon guidance
135
110
112
84
34
73
10
42
46
24
0
3
6
9
−log10 FDR
D
HuD
Tubulin
Control
HuD overexpression
roots
segments
extremities
Control
HuD oe
Proportion
***
***
***
0
1
2
extremities
roots
segments
E
T7
tubulin
HIS HA
tubulin
T7_GFP
T7_HuD_sv1
Proportion
*
*
max_neurite_length
tot_neurite_length
HIS_HA_empty
HIS_HA_HuD_sv2
Proportion
**
**
max_neurite_length
tot_neurite_length
0
2
4
6
8
10
F
Fold ratio
(HuD overexpr vs ctrl)
Translation Efficiency
Polysomal RNA
Total RNA
0
1
2
3
4
Kif5b
Sema4d
Picalm
Acsl4
Hnrnpa2b1
Ppp2ca
Cdc42
B2m
Pfn1
Rac1
Ppia
G
Amyotrophic lateral sclerosis mouse GSE10953
Spinal muscular atrophy mouse GSE3075
Amyotrophic lateral sclerosis mouse GSE4390
Amyotrophic lateral sclerosis mouse GSE3343
Amyotrophic lateral sclerosis rat GSE7493
C9orf72 Spinal cord Knockout GSE77681
Amyotrophic lateral sclerosis human GSE833
Spinal Cord Injury rat GSE2599
ALS type 10 human GSE18632
121
111
98
100
69
94
81
59
43
0
10
20
30
−log10 FDR
H
Fold ratio
(HuD overexpr vs ctrl)
Translation Efficiency
Polysomal RNA
Total RNA
0
1
2
3
4
Vcp
Eif4a2
Setd8
Ubqln2
Cd47
Cox7a2l

Figure S3. Related to Figure 3.

(A) Starvation in NSC-34 cells induces neither stress granule nor P-bodies formation. Left panel: NSC-34 were immunostained for Pabp1 (green) and Tia1 (red) to detect stress granules formation. Arsenite treatment was used as positive control for stress granules formation. The images show that starvation per se does not induce stress granules and does not increase arsenite-triggered stress granules. Right panel: NSC-34 were immunostained for Pabp1 (green) and Dcp1a (red) that is a P-bodies marker. Arsenite treatment was used as positive control for increasing P-bodies' size and number. The images show that starvation does not alter P-bodies in neither size nor number. The scale bars correspond to 40 μm.

(B) Measurement of global TE by sucrose gradient centrifugation as the ratio between the area under polysome peaks and the area under the 80S peak in the following conditions: control, Torin 1 treatment and Torin 1 treatment coupled with HuD overexpression. (data are represented as mean ± SEM. "Torin" was compared to "Control", and "Torin + HuDoe" was compared to "Torin" for testing statistical significance).

(C) Gene ontology neuronal-related enriched terms among HuD targets.

(D) Left: automated neurite segmentation performed with an Operetta HCS device (right). The cells were imaged on the High Content Screening System Operetta™ (PerkinElmer). The cytoplasm was immunostained with anti-tubulin antibody (green) to detect neuritic arborization, whereas the nuclei was identified by Hoechst staining (blue). The number of neurite segments, of extremities and of roots were quantified. Center: representative images of differentiated NSC-34 cells (control or overexpressing HA-tagged HuD) immunostained with anti-HA tag (red) and anti-tubulin antibodies (green); Right: neurite outgrowth quantification in differentiated NSC-34 cells overexpressing HA-tagged HuD compared to control.

(E) left: Representative images of PC12 cells overexpressing T7-HuD or HA-tagged HuD, immunostained with anti-HA or anti-T7 (green) and anti-tubulin antibodies (red); right: Maximum and total neurite length were analyzed using High content screening (HCS).

(F) Measurement of TE, polysomal RNA and total RNA variations on specific neuronal-related targets in HuD overexpressing cells compared to control. TE was calculated as the ratio between the polysomal and total RNA quantified by targeted sequencing and displayed in the plot in green and grey, respectively. HuD increases the TE of several neuronal-related targets, supporting neuronal differentiation.

(In panels B,C data are represented as mean ± SEM. t-test $^{*}p < 0.05$, $^{**}p < 0.01$ and $^{***}p < 0.001$).

(G) Enrichment of HuD targets among collections of genes with altered expression levels in motor neuron diseases (available in enrichR libraries).

(H) Measurement of TE, polysomal RNA and total RNA variations of HuD targets related to motor neuron diseases upon HuD overexpression in NSC-34 cells. TE was calculated as the ratio between the polysomal and total RNA quantified by targeted sequencing and displayed in the plot in green and grey, respectively.

(In panels B,D, E, F and H data are represented as mean ± SEM. t-test $^{*}p < 0.05$, $^{**}p < 0.01$ and $^{***}p < 0.001$).

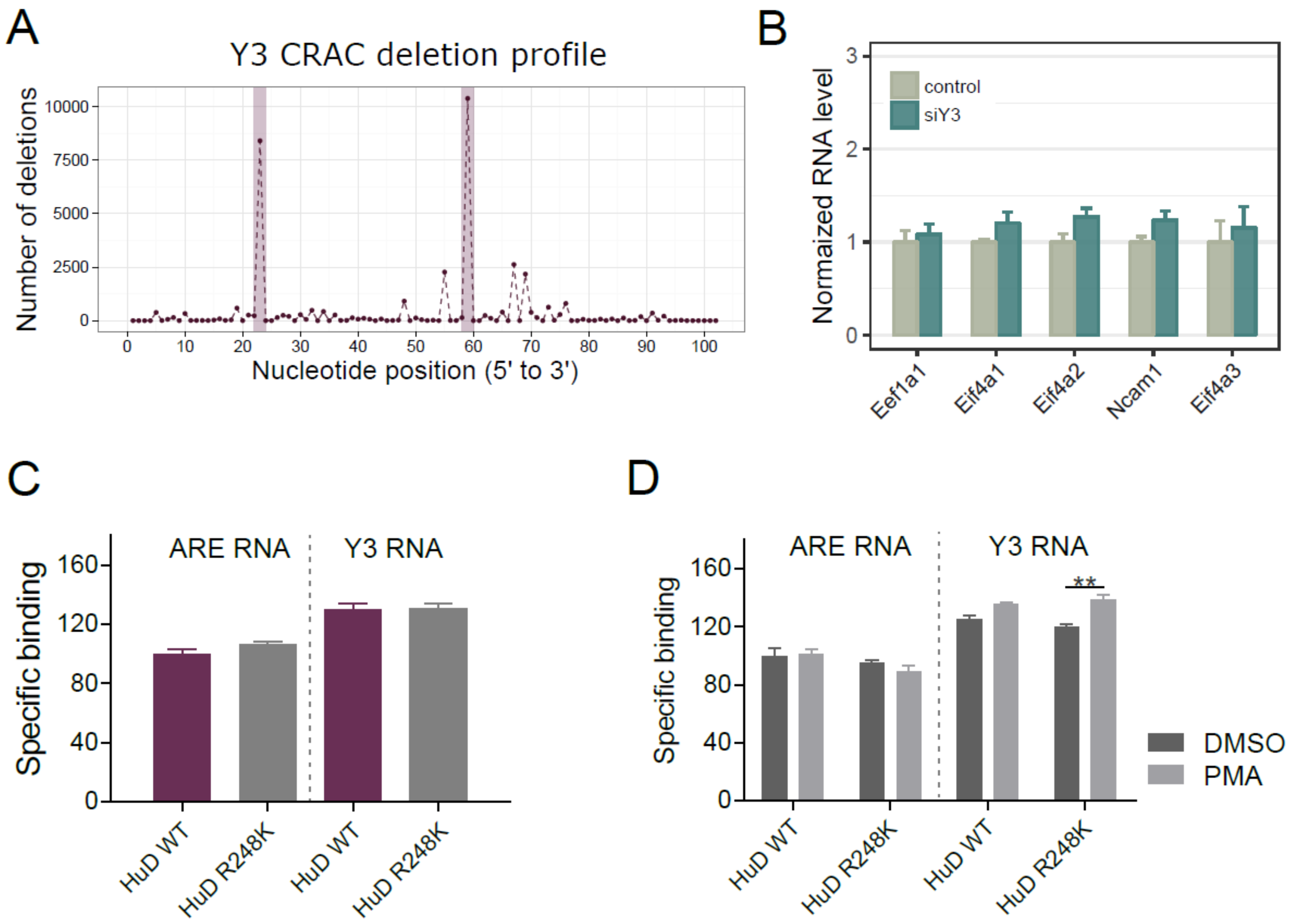

Figure S4. Related to figure 4.

(A) Y3 deletion profile, based on HuD CRAC. HuD binds to Y3 in two discrete locations, both positioned in polypirimidine tracts in the looped region outside the Y3 structural stem.

(B) mRNA levels of HuD target genes in control (scramble) or Y3 depleted cells (siY3) normalized to Gapdh. The graph is shown as mean ± SEM.

(C) Alpha screen assessing the specific binding of tagged-HuD and -HuD R248K proteins with ARE and Y3 RNA probes (C). Two independent experiments were performed at the hooking point with 50 nM of RNA probes.

(D) AlphaScreen assay measuring the specific binding of tagged-HuD and -HuD R248K proteins with ARE and Y3 RNA probes under PMA treatment for 45 min (D). Two independent experiments were performed at the hooking point with 50 nM of RNA probes.

(In panel D, data are represented as mean ± SEM. t-test **p < 0.01)

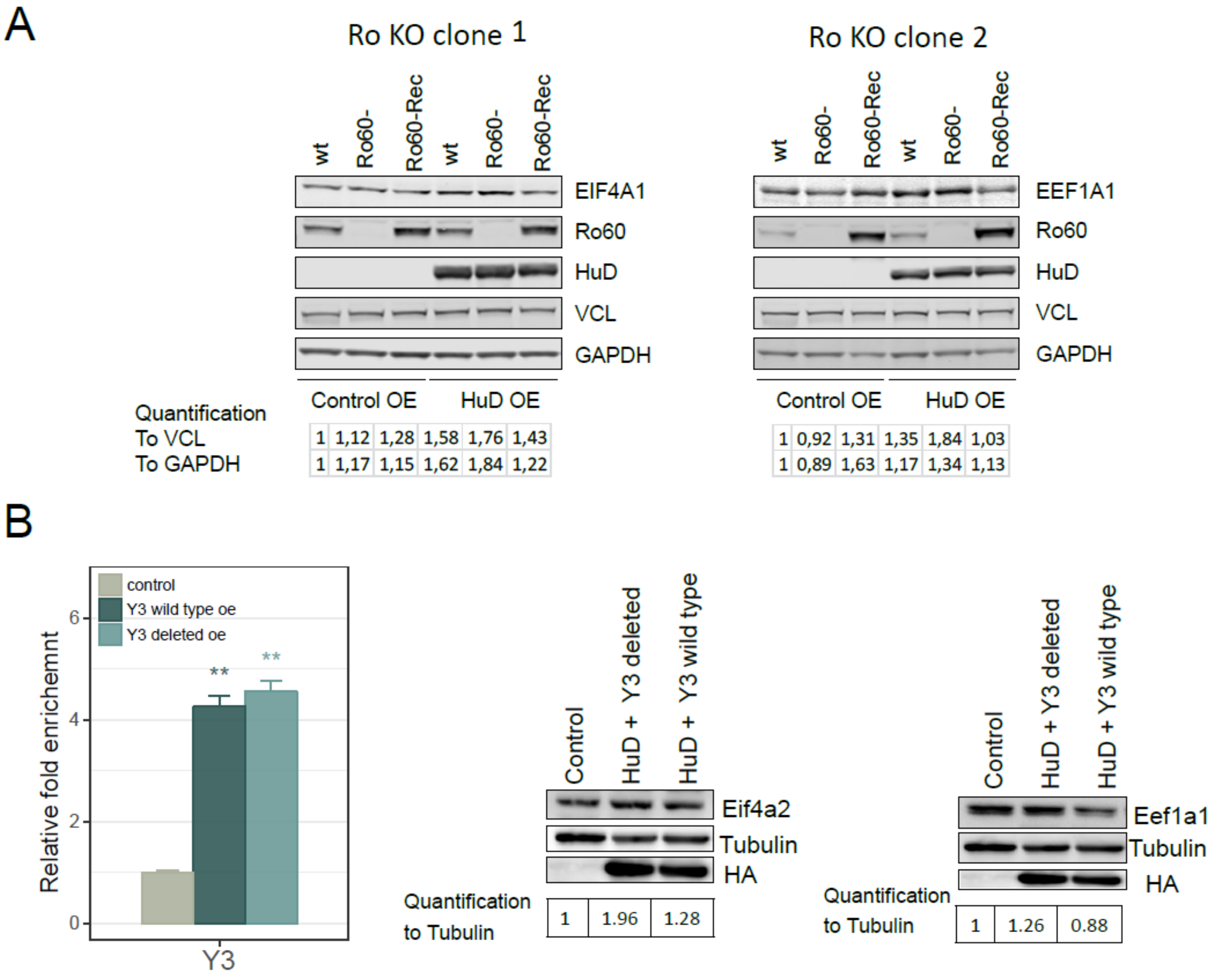

Figure S5. Related to Figure 5.

(A) The human ovarian cancer cell line ES2 was used to study the Ro60/Y RNA dependence of HuD activity on one of its target mRNAs. The set of wt cells, Ro60-knockout cells (Ro60-) and KO-cells with Ro60 recovery (Ro60-Rec) was either transfected with empty plasmid or HuD plasmid in both knockout clones. The HuD targets EIF4A1 and EEF1A1 were quantified by Western Blotting.

(B) HEK-293 cells were co-transfected with HIS-HA-HuD and deleted or wild type Y3. Left panel: qPCR assay confirmed the equal expression both Y3 wild type and Y3 mutant after transient transfection. 5S rRNA was used as housekeeping gene for normalization . Data are represented as mean ± SEM. t-test $**p < 0.01$. Right panel: the effect of the deletion was measured by western blotting on the HuD targets Eif4a2 and Eef1a1 by using α-Tubulin as a reference gene. HA antibody was used to verify HuD overexpression.

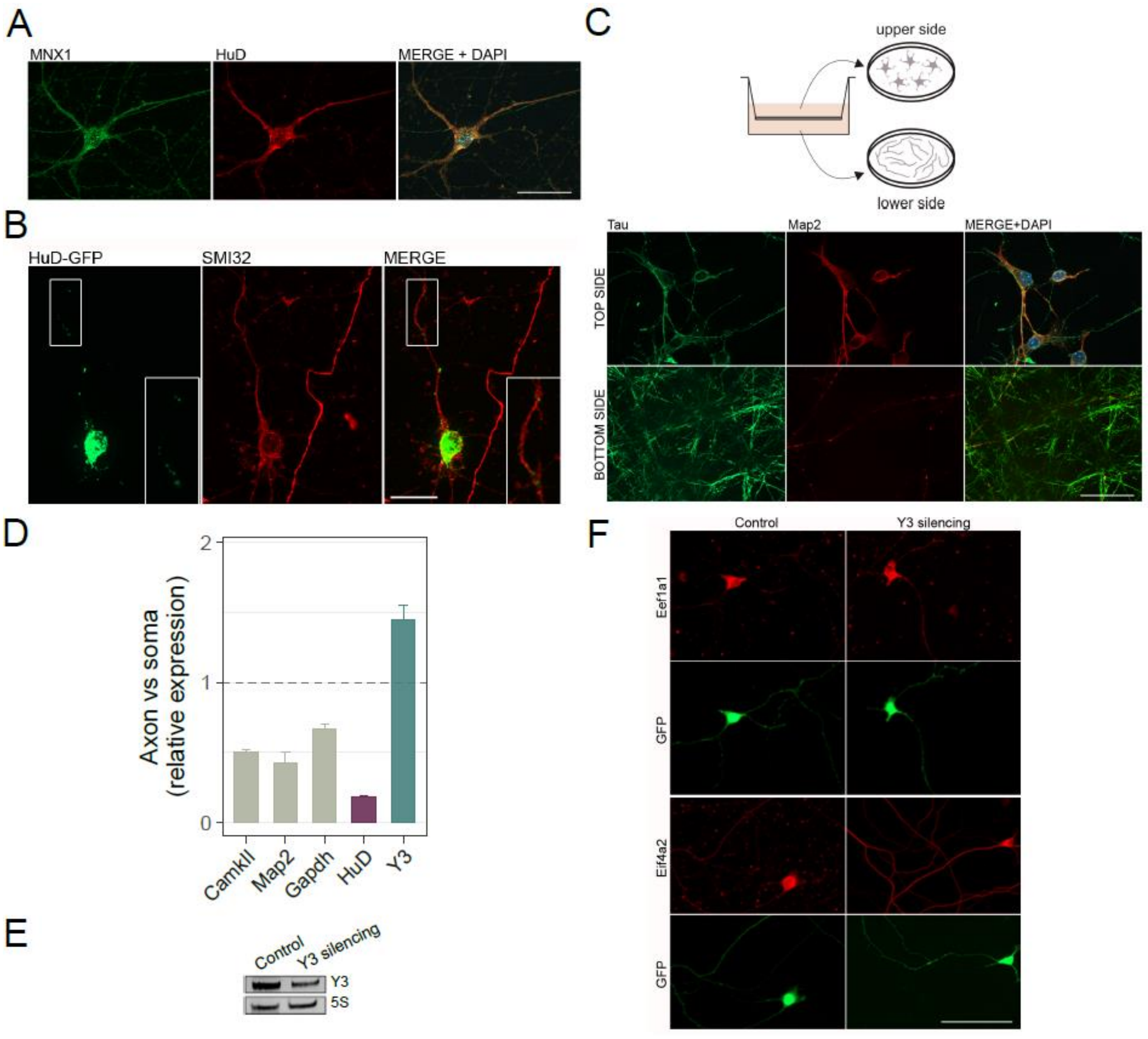

Figure S6. Related to Figure 5. HuD and Y3 are both expressed in motor neurons with a Y3 enrichment in axonal compartment.

(A) Primary motor neurons (5 DIV) immunostained for the motor neuronal marker Mnx1 (Hb9, green) and HuD (red). The scale bar corresponds to 40 μm.

(B) Motor neurons immunostained for the motor neuronal marker SMI32 after 48 hours transfection with pHuD-GFP vector. Both endogenous and transfected HuD shows a granular staining in the cell body, dendrites and along the axons. The scale bar corresponds to 20 μm.

(C) Top: Schematic overview of the separation system. Embryonic motor neurons were cultured in tissue culture inserts fitted with PET membrane with 3 μm pores. Neuron cell bodies remained on the upper membrane surface, whereas axons and few dendrites passed through membrane pores reaching the lower side of the membrane. Bottom: Immunofluorescent staining of motor neurons on PET membrane: neuronal

processes were marked with Tau (green), dendrites Map2 (red), cell nuclei with DAPI (blue). Images confirm that mainly axons (just few dendrites) cross the membrane and reaches the bottom side, with no contamination of cell bodies. On the contrary, cell bodies and dendrites were retained on the top side. The scale bar corresponds to 40 μm.

(D) RT-qPCR analysis of RNA extracted from motor neuron axons (scraped from the membrane bottom side) and compared with RNA extracted from cell bodies and dendrites (scraped from the membrane top side). Relative expression data show that *Y3* RNA is more present in the axons compared to cell soma and dendrites, while *HuD* and *Map2* are more localized to cell soma and dendritic tree.

(E) Assessment of the effective silencing of *Y3* RNA using the Y3-short hairpin vector.

(F) Immunostaining of Eef1a1 and Eif4a2 protein levels in primary motor neurons transfected with an shRNA construct directed against Y3 (sh_Y3) or a control vector (sh_Ctrl). GFP (green) identifies transfected cells that were subjected to quantification of either Eef1a1 or Eif4a2 signal (red). Acquisition and quantification was performed by Operetta HCS device. (n = >20 cells/condition).

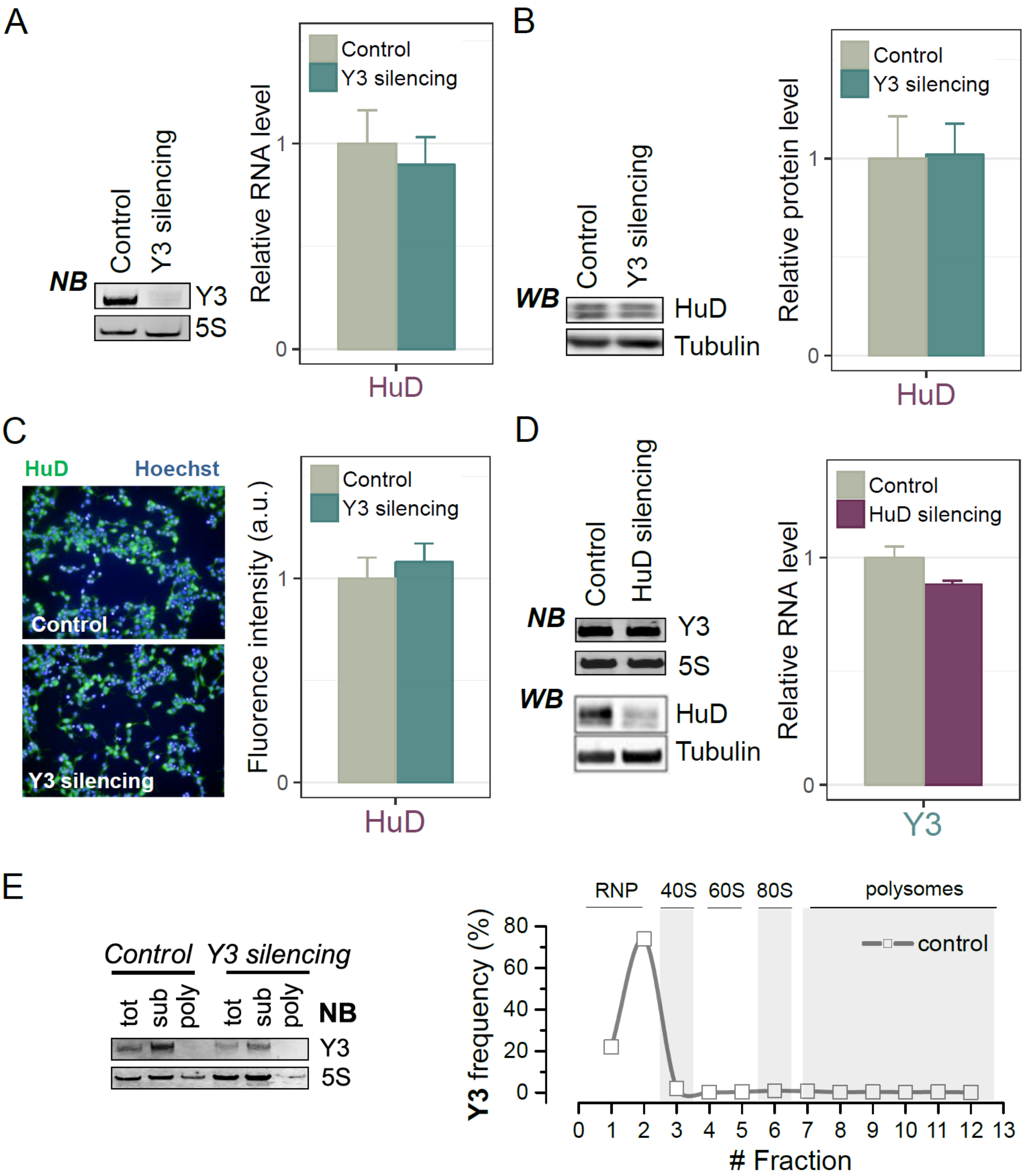

Figure S7. Related to Figure 6. HuD and Y3 do not mutually influence their expression.

(A) Left: Northern blot showing the efficient silencing of NSC-34 transfected with si-Y3 in comparison with control (scramble). 5S is used as housekeeping; right: level of HuD transcript quantified by RT-qPCR in si-Y3 NSC-34 cells compared to control. Rpl10a and Gapdh were used as reference.

(B) HuD protein levels quantified by Western Blot in control and Y3-siRNA cells.

(C) HuD protein levels quantified by immunofluorescence in control and Y3-siRNA cells.

(D) Y3 quantification by Northern blot in control and si-HuD cells. Western Blotting showing silencing of the HuD protein is also shown.

NB, WB and IF images are representative of at least three biological replicates.

(E) Left panel: Y3 quantification in total, subpolysomal and polysomal RNA fractions by Northern blot in control and Y3 depleted NSC-34 cells. Right panel: semi-quantitative analysis of Y3 relative transcript levels along the sucrose gradient fractions of control NSC-34 cells. Y3 is primarily localized in the RNP fractions.

Supplementary Tables

Target name	Forward primer	Reverse primer
**Als2**	catcatgttcaccaccttgaa	ctgtcaggcgccctagtta
**Bdnf**	caggaggaatttctgagtggcca	gcagaaggcctaagcaacttgac
**Eef1a1**	agtcacccgcaaagatgg	cgagttggtggtaggatacaatc
**Eef1b2**	acattgatctctttggatctgatg	gatggaagacttcgcaacaac
**Eif2a**	acttttagtaaggatgggacattgtt	tcccttgttagcgacattga
**Eif2s3x**	agccccgtctcattgtca	cacctcccttaaggtcatcg
**Eif4a1**	ggatcatgtctgcgagtcag	gctatccacaatctcgttcca
**Eif4a2**	cgatctacctaccaatcgtgaa	acctttcctcccaaatcgac
**Eif4a3**	cgaggaggtggacgtgac	tgctgaatcgctgaaggttt
**Eif4g2**	aaaggacagcttaatgcagatga	tgcactgggaggaatcatagt
**Eif4h**	tcaggaaaggtggacctgat	tcccatccacctctagattctc
**Gapdh**	ggaagggctcatgaccacag	acagtcttctgggtggcagtg
**HuD**	cagtgaacaacgtcaaggtcatc	ccttgcacttgttggtgttgaa
**Pabpc1**	ggtgccagacctcatcca	tgtgaggaagctggtctcatc
**Y3**	ggttggtccgagagtagtgg	aaaggctggtcaagtgaagc
**Rpl10a**	gaagaaggtgctgtgtttggc	tcggtcatcttcacgtggc
**Rpl32**	gggagcaacaagaaaaccaa	attgtggaccaggaacttgc
**Rps20**	tcctgactcaccgctgttc	ggcgtctttccggtatcttt
**Map2**	tgccacctgtttctctccac	tcttttgcttgctcgggatt
**Ncaml**	agggcaaggctgctttct	ccccatcatggtttggagt
**CamKII**	cacactcacaccacttcctt	gcattccatacaagagccaaac

Sample Name	Barcode	Read length after barcode removal
**CRAC_CTRL_1**	NNNCGCTTAGC	89
**CRAC_CTRL_2**	NNNTAAGC	92
**CRAC_CTRL_3**	NNNACTCAGC	90
**CRAC_HUD_1**	NNNGCGCAGC	90

**CRAC_HUD_2**	NNNATTAGC	91
**CRAC_HUD_3**	NNNGACTTAGC	89

Table S3. Related to STAR Methods.
Primer Sequences Used for Real-Time PCR and barcodes used for CRAC.
