# Supplementary material for: HuD Is a Neural Translation Enhancer Acting on mTORC1-Responsive Genes and Counteracted by the Y3 Small Non-coding RNA
Source: Mol Cell. 2018 Jul 19;71(2):256–270.e10. doi: 10.1016/j.molcel.2018.06.032 (PMC6060611; doi:10.1016/j.molcel.2018.06.032)
Supplement: Document S2. Article plus Supplemental Information [file mmc4.pdf]

Article

Molecular Cell

HuD Is a Neural Translation Enhancer Acting on mTORC1-Responsive Genes and Counteracted by the Y3 Small Non-coding RNA

Graphical Abstract

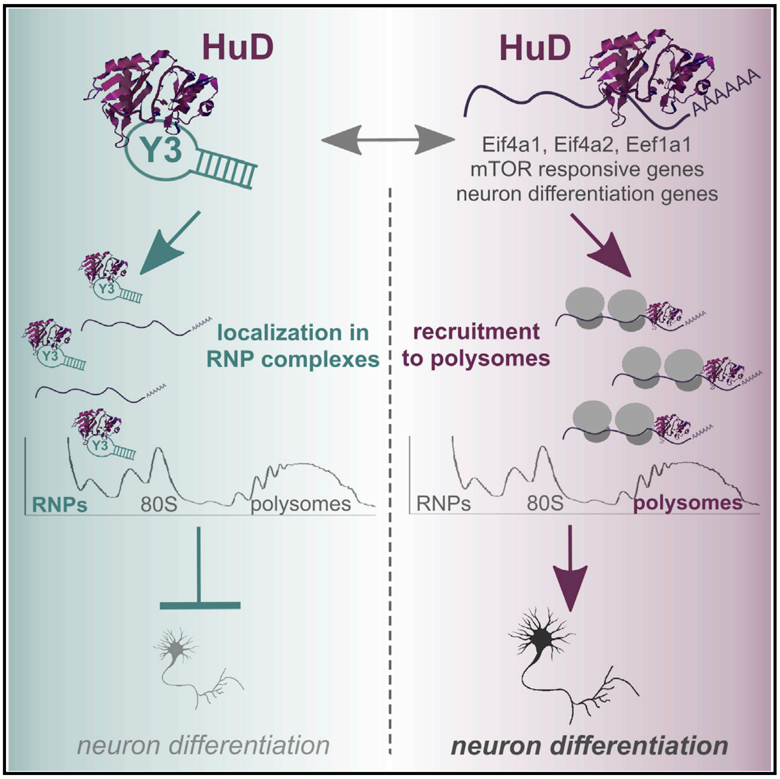

Highlights

- HuD enhances the efficiency of translation in a mTORC1-independent fashion
- The small non-coding RNA Y3 is the predominant HuD target in motor neuron-like cells
- Y3 sequesters HuD from polysomes and counteracts its translation enhancer activity
- Y3 blocks the function of HuD in neuronal differentiation

Authors

Toma Tebaldi, Paola Zuccotti, Daniele Peroni, ..., David Tollervey, Stefan Hüttelmaier, Alessandro Quattrone

Correspondence

alessandro.quattrone@unitn.it

In Brief

Tebaldi et al. report that the neuronal RNA binding protein HuD increases translation. The small non-coding RNA Y3 is a molecular sponge of HuD, limiting its recruitment to polysomes and neuron differentiation. These findings uncover an alternative route to the mTORC1 pathway for translational control, tunable by a non-coding RNA.

Tebaldi et al., 2018, Molecular Cell *71*, 256–270
July 19, 2018 © 2018 The Authors. Published by Elsevier Inc.
https://doi.org/10.1016/j.molcel.2018.06.032

Molecular Cell
Article

HuD Is a Neural Translation Enhancer Acting on mTORC1-Responsive Genes and Counteracted by the Y3 Small Non-coding RNA

Toma Tebaldi,[1,10,11] Paola Zuccotti,[1,10] Daniele Peroni,[1,10] Marcel Köhn,[2,3,10] Lisa Gasperini,[4] Valentina Potrich,[1] Veronica Bonazza,[1] Tatiana Dudnakova,[5] Annalisa Rossi,[4] Guido Sanguinetti,[6] Luciano Conti,[7] Paolo Macchi,[4] Vito D'Agostino,[8] Gabriella Viero,[9] David Tollervey,[5] Stefan Hüttelmaier,[2] and Alessandro Quattrone[1,12,*]
[1]Laboratory of Translational Genomics, Centre for Integrative Biology, University of Trento, Trento 38123, Italy
[2]Institute of Molecular Medicine, Martin-Luther-University Halle-Wittenberg, Halle 06120, Germany
[3]Julius-Bernstein-Institute of Physiology, Martin-Luther-University Halle-Wittenberg, Halle 06097, Germany
[4]Laboratory of Molecular and Cellular Neurobiology, Centre for Integrative Biology, University of Trento, Trento 38123, Italy
[5]Wellcome Trust Centre for Cell Biology, University of Edinburgh, Edinburgh EH9 3BF, UK
[6]School of Informatics, University of Edinburgh, Edinburgh EH8 9AB, UK
[7]Laboratory of Stem Cell Biology, Centre for Integrative Biology, University of Trento, Trento 38123, Italy
[8]Centre for Integrative Biology, University of Trento, Trento 38123, Italy
[9]Institute of Biophysics, CNR Unit at Trento, Trento 38123, Italy
[10]These authors contributed equally
[11]Present address: Yale Cancer Center, Yale University School of Medicine, New Haven, CT 06520, USA
[12]Lead Contact
*Correspondence: alessandro.quattrone@unitn.it
https://doi.org/10.1016/j.molcel.2018.06.032

SUMMARY

The RNA-binding protein HuD promotes neurogenesis and favors recovery from peripheral axon injury. HuD interacts with many mRNAs, altering both stability and translation efficiency. We generated a nucleotide resolution map of the HuD RNA interactome in motor neuron-like cells, identifying HuD target sites in 1,304 mRNAs, almost exclusively in the 3′ UTR. HuD binds many mRNAs encoding mTORC1-responsive ribosomal proteins and translation factors. Altered HuD expression correlates with the translation efficiency of these mRNAs and overall protein synthesis, in a mTORC1-independent fashion. The predominant HuD target is the abundant, small non-coding RNA Y3, amounting to 70% of the HuD interaction signal. Y3 functions as a molecular sponge for HuD, dynamically limiting its recruitment to polysomes and its activity as a translation and neuron differentiation enhancer. These findings uncover an alternative route to the mTORC1 pathway for translational control in motor neurons that is tunable by a small non-coding RNA.

INTRODUCTION

The intensively studied RNA-binding protein (RBP) *hu*man antigen *D* (HuD)/*e*mbryonic *l*ethal, abnormal *v*ision *l*ike 4 (ELAVL4) is predominantly expressed in differentiated neurons, as are the other neuronal members (nELAV) of the ELAV family, HuB (ELAVL2) and HuC (ELAVL3). In contrast, HuR (ELAVL1) is ubiquitously expressed (Pascale et al., 2008). HuD carries three *R*NA *r*ecognition *m*otif (RRM) domains and plays important roles in controlling the fate of many neuronal mRNAs. Functional analyses implicate HuD in the regulation of mRNA stability, alternative splicing, alternative polyadenylation, RNA localization, and translation (Bronicki and Jasmin, 2013).

HuD is one of the first markers expressed during neuronal differentiation and plays a fundamental role in controlling neuronal cell fate. Loss of HuD induces increased self-renewal of the neural stem and progenitor cells (Akamatsu et al., 2005), whereas overexpression promotes neurite outgrowth, neurogenesis, and neuronal plasticity (Perrone-Bizzozero and Bolognani, 2002).

Importantly, HuD is specifically implicated in motor neuron function, and HuD knockout mice show motor deficits (Akamatsu et al., 2005), while regeneration following peripheral axon injury is associated with increased levels of HuD and of its target GAP43 (Anderson et al., 2003). Recent studies pointed out the intimate relationship between HuD and motor neuron diseases. HuD has been characterized for its ability to localize mRNAs in primary motor neurons and restore axon outgrowth defects in spinal muscular atrophy (SMA) motor neurons (Akten et al., 2011; Fallini et al., 2011). Moreover, cytoplasmic inclusions of TDP-43, a pathological hallmark of amyotrophic lateral sclerosis (ALS), are proposed to sequester HuD (Fallini et al., 2012).

To understand the molecular mechanism that underpins the functions of HuD, we first sought to positionally identify its RNA targets in a comprehensive way. Selective antibodies for individual nELAV paralogs are currently not available, so cross-linking and immunoprecipitation (CLIP) analysis identified only RNAs cumulatively bound to nELAV proteins HuB, HuC, and HuD (Scheckel et al., 2016). Specific HuD targets were

 © 2018 The Authors. Published by Elsevier Inc.
This is an open access article under the CC BY license (http://creativecommons.org/licenses/by/4.0/).

previously identified by immunoprecipitating HuD from a HuD-overexpressing mouse (Bolognani et al., 2010). However, this approach could not provide positional information on the binding sites on RNA or distinguish between direct and indirect targets. To overcome these limitations, we specifically characterized the RNA interactome of HuD using the CRAC (cross-linking and analysis of cDNAs) method (Granneman et al., 2009). We performed our analysis in NSC-34 cells, which recapitulate motor neuron phenotypes *in vitro*.

We found that HuD directly and specifically enhances the translation efficiency of mRNAs known to be involved in motor neuron differentiation and axonogenesis. Surprisingly, we also found that a major HuD-bound cluster contains mRNAs encoding components of the translational machinery. HuD translation enhancer activity is independent from the major pathway affecting general translation, controlled by the mTORC1 complex, despite targeting an overlapping set of mRNAs.

Remarkably, the Y3 small noncoding RNA (ncRNA) was by far the strongest HuD binding partner. Y RNAs are abundant ncRNAs transcribed by RNA polymerase III (Köhn et al., 2013; Kowalski and Krude, 2015), ranging in size from 70 to 115 nt and folding into characteristic stem-loop structures. Y RNAs were proposed to be involved in DNA replication and histone mRNA processing (Köhn et al., 2015). However, their biological functions are still largely elusive. Here, we demonstrate that Y3 acts as a molecular sponge for HuD activity, by competing with HuD target mRNAs and by limiting HuD access to the polysomal compartment.

RESULTS

Identification of the HuD RNA Interactome in a Motor Neuron Cell Line

HuD shares a high sequence and structure similarity with the other members of the ELAV family, and all available antibodies fail to distinguish among them. To overcome this difficulty, we adapted the CRAC protocol to be used with mouse motor neuron NSC-34 cells engineered with doxycycline-inducible His-HA tagged HuD. We performed the CRAC experiment using doxycycline at 2 ug/ml for 48 hr to limit HuD levels to physiological values (Figures S1A–S1C). We used doxycycline-treated cells expressing only the tetracycline receptor (Trex cells) as control for the aspecific signal (Figure 1A and STAR Methods).

To precisely map the HuD RNA interactome, we developed a dedicated computational methodology (see also STAR Methods). This approach takes advantage of cross-linking induced mutations—primarily micro-deletions—to identify candidate binding sites with nucleotide resolution (Figure 1B). To increase specificity, we penalized locations with aligned reads in control experiments. We selected a set of 753 sequences surrounding locations with p value <0.05 to build a positional weight matrix (PWM). This "seed" PWM was defined on a region spanning 7 nt around the deletion site (Figure S1D). The size choice is based on previous crystallographic studies resolving the structure of the RRM1 and RRM2 domains of HuD bound to canonical AU rich elements (Wang and Tanaka Hall, 2001). We used the seed PWM to score all the other candidate binding sites and select high-confidence HuD bound sites, with the advantage of identifying in this way interaction sites even in transcripts with low expression levels. The strength of this methodology is revealed by the comparison between the distribution of scores associated with CRAC deletion sites and the distribution of random sequences (Figure 1C). The experimental distribution is peaked above the threshold score corresponding to the 95th percentile of the random distribution.

We performed parallel RNA-Seq in NSC-34 cells to quantify the steady-state levels of transcripts. Of note, HuD binding site intensities showed a low positive correlation with transcript levels (measured by FPKM, Pearson correlation = 0.24) (Figure S1E). Binding affinity could therefore be the main factor influencing peak intensity. We normalized CRAC binding site intensities for transcript levels, and we created a logo representation where each HuD binding site is weighted for its binding affinity (Figure 1D). The core of the resulting HuD affinity logo contains a triplet of U nucleotides (weight = 1), preceded by a non-U nucleotide with the following weights in decreasing order: C (weight = 0.40), A (weight = 0.32), and G (weight = 0.28). This result suggests that HuD binding affinity is similarly strong for canonical AU-rich elements (AUUU); GU-rich elements (GUUU, also reported as the main nELAV binding site in Scheckel et al., 2016); and in particular CU-rich elements (CUUU). Of note, CU-rich and GU-rich related elements were indirectly identified as HuD binding motifs also in Bolognani et al. (2010).

Our approach detected 5,153 high-confidence binding regions, mapped on 1,304 protein coding genes and 131 ncRNAs (Figure 1E; Table S1). Among the ncRNAs, 10 were long intergenic ncRNAs (lincRNAs) including Neat1, Malat1, and Yam1, known to be involved in cell-fate programming. Strikingly, the by far predominant HuD binding sites were found on the Y3 small ncRNA, representing 70% of all binding signal (Figure 1E).

We further validated interactions identified by CRAC with RNA immunoprecipitation (RIP) for 70 mRNAs and for the Y3 RNA. For the mRNAs tested, RIP followed by targeted sequencing confirmed the identification of bona fide HuD binding sites by CRAC, with a median log2 fold enrichment of 5.8 (Figure 1F; Table S2). We selectively enriched Y3 together with the positive control *Bdnf* mRNA in HuD ribonucleoprotein particles, but not in negative control cells (Figure 1G, left panel). For both conditions, no binding to the *Rpl10a* transcript (negative control mRNA) was detected. His-tag non-specific interactions were excluded by additional RIP assays in NSC-34 cells overexpressing His-HA-GFP or with a reduced HuD induction (Figure S1F). The interaction between HuD and Y3 was further confirmed in NSC-34 transiently transfected with SBP-tagged HuD (Figure 1G, right panel). No binding was detected for the Y1 small ncRNA, the only other member of the Y RNA family in the mouse genome, nor for the highly abundant small ncRNA signal recognition particle RNA (7SL). Additionally, we performed a pull-down assay by using Y3, Y1 and human Y4 (hY4) ncRNAs, as synthetic biotinylated probes, in both NSC-34 induced for HuD and in control cells. We demonstrated specific association between HuD and Y3 (Figure 1H, right panel).

In summary, we reliably profiled the HuD RNA interactome in NSC-34 cells, identifying the Y3 ncRNA as the by far most represented target.

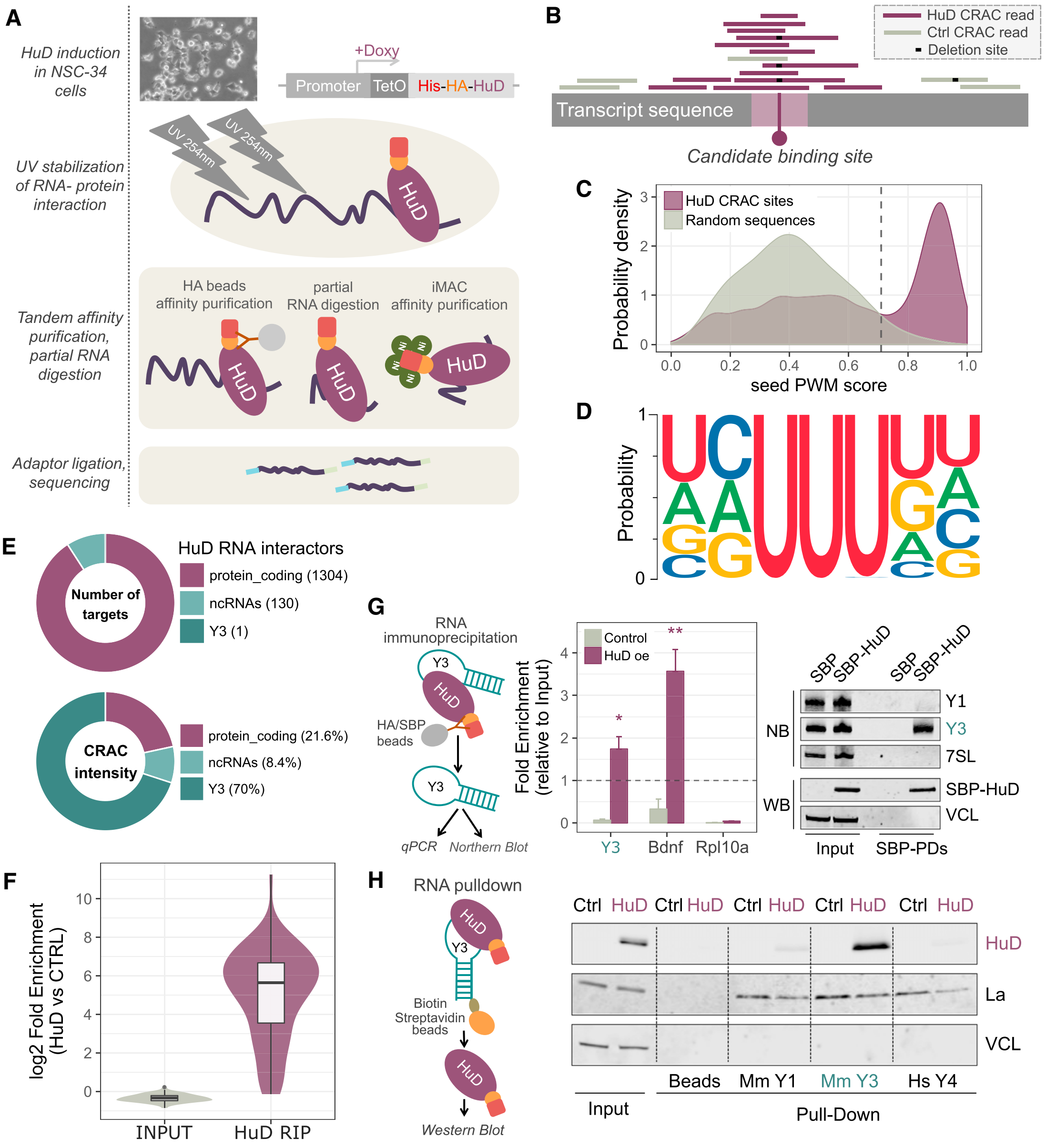

Figure 1. Defining the RNA Interaction Landscape of HuD in Motor Neuron Cells

(A) Schematic representation of CRAC performed on motor neuron NSC-34 cells.

(B) Identification of HuD binding sites from CRAC data.

(C) Distribution of HuD PWM scores, calculated from CRAC deletion sites (in violet) and compared with random sequences (in gray). The score threshold to identify bona-fide binding sites was set as the 95th percentile of the random distribution (vertical dashed line).

(D) Logo representation of HuD binding sites weighted by binding affinity, calculated as CRAC binding intensities scaled for transcript expression levels.

(E) Pie charts displaying the number of HuD RNA targets (upper panel) and the corresponding interaction weight (percentage of CRAC intensity, lower panel) for distinct RNA species.

(F) Validation by RNA immunoprecipitation (RIP) and targeted sequencing of 70 HuD targets identified by CRAC.

(G) Validation of HuD-Y3 interaction by alternative approaches: left panel, RIP assay followed by Northern blots in HuD transfected NSC-34 cells; right panel, RIP assay followed by RT-qPCR in NSC-34 HuD-inducible cells and in Trex NSC-34 cells (control). In (G), data are represented as mean ± SEM; t test: *p < 0.05, **p < 0.01.

(H) Streptavidin pull-down of synthetic biotinylated Y RNAs (Y3, Y1, and human Y4) followed by western blot analysis in NSC-34 cells induced for HuD expression. The La (SSB) and Vinculin (VCL) proteins were used as positive and negative control proteins, respectively, for binding to Y RNAs.

See also Figure S1 and Table S1.

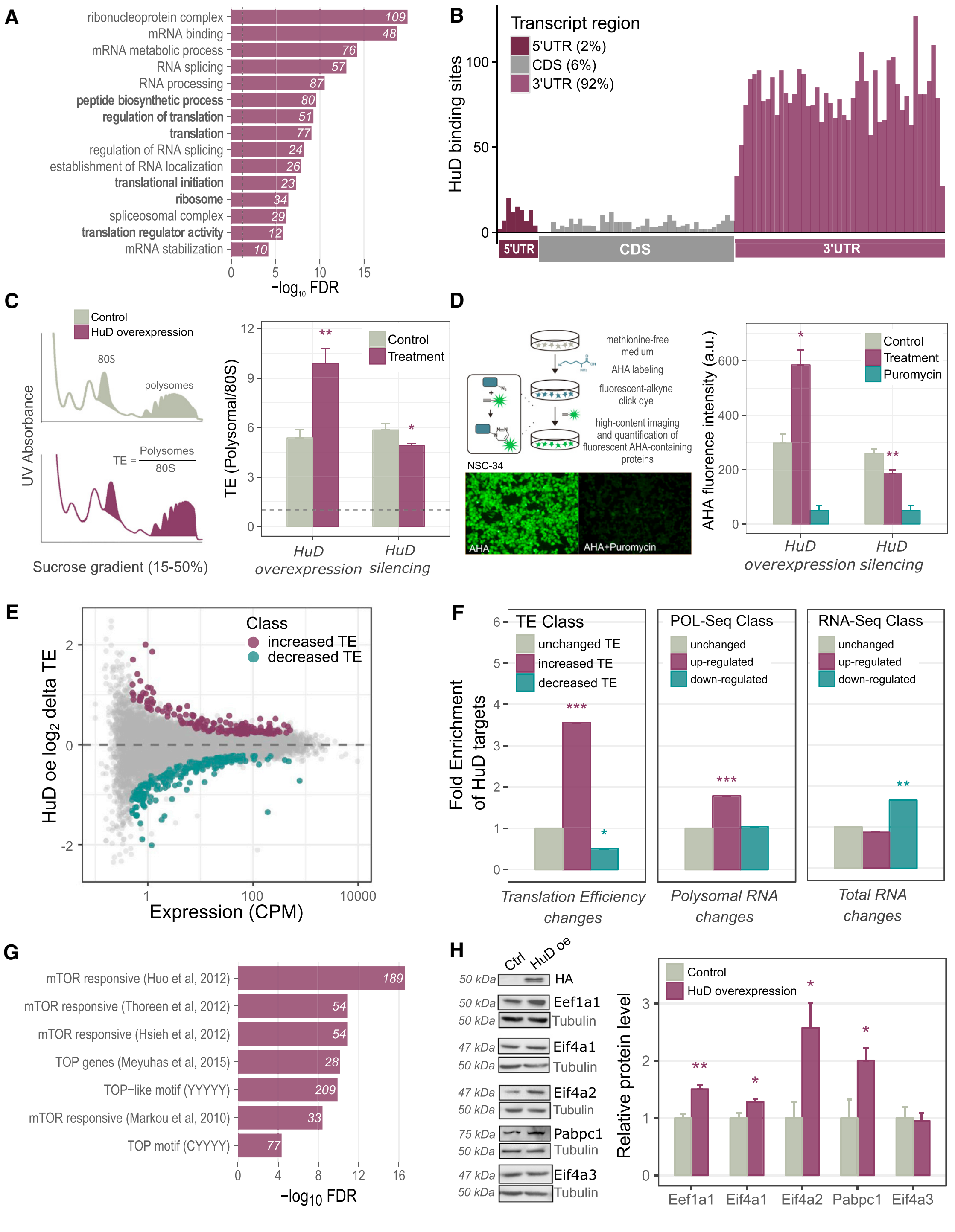

Figure 2. HuD Increases Global and Target-Specific Translation

(A) Top enriched Gene Ontology terms among HuD mRNA targets are related to RNA processes, including splicing, transport, stability, and translation (highlighted in bold).

(B) Metaprofile of HuD binding sites along protein coding transcripts, showing binding enrichment in 3′UTRs.

(C) Right panel: representative sucrose gradient profiles in control and HuD overexpressing NSC-34 cells. Left panel: calculation of the global translation efficiency upon HuD silencing and overexpression.

(D) Right: schematic representation of Click-iT AHA assay to quantify *de novo* protein synthesis in NSC-34 cells. Left: detection of *de novo* protein synthesis upon HuD silencing and overexpression. Puromycin, a translation inhibitor, was used as negative control.

(legend continued on next page)

HuD Enhances the Translation of Target Translation Factors

To provide a functional characterization of HuD-interacting RNAs, we performed enrichment analysis of Gene Ontology (GO) terms and pathways (Figure 2A). We identified significant enrichments for terms related to genes involved in mRNA processing and translation: 80 genes, including 34 ribosomal components and 12 translation initiation or elongation factors. Within mRNA targets, HuD binding sites were predominantly located in the 3′ UTR of protein coding transcripts (92%), consistent with functions in translation (Figure 2B).

The widespread HuD binding to mRNAs encoding ribosomal proteins and translation factors suggested that HuD could indirectly promote global translation through the post-transcriptional modulation of these mRNAs. We therefore assessed the role of HuD in modulating global translation by polysome profiling in NSC-34 cells with the overexpression or silencing of HuD (Figures S2A and S2B). The global translation efficiency (TE) of the cells was calculated as the ratio between the absorbance of polysomes and the total absorbance of non-translating 80S ribosomes (see STAR Methods and Figure 2C). As shown in Figure 2C, HuD overexpression significantly increased the global TE of NSC-34 cells. Conversely, HuD depletion by RNA interference resulted in a reduced global TE. To support this finding, we assessed the ability of HuD to promote *de novo* protein synthesis by metabolic labeling (see STAR Methods). We measured a substantial increase (about 2-fold) in overall *de novo* protein synthesis in HuD-overexpressing cells compared to control cells, whereas knockdown of HuD resulted in *de novo* protein synthesis reduction (Figure 2D).

We further confirmed the role of HuD as a translational enhancer of its targets by combining RNA-Seq and POL-Seq (polysomal RNA sequencing) of NSC-34 cells upon HuD overexpression (Figure 2E). Translation efficiency was defined for each gene as the ratio between POL-Seq and RNA-Seq levels. Importantly, HuD targets were strongly enriched in genes with increased translation efficiency (fold enrichment = 3.6, p value = 7.6e-10) and, conversely, underrepresented in genes with decreased translation efficiency (Figure 2F, right panel). This level of enrichment was observed only combining translatome and transcriptome variations and derived mainly from translatome effects (Figure 2F). On the other hand, transcriptome-wide alternative polyadenylation (APA) analysis upon HuD overexpression didn't reveal an enrichment of HuD targets among genes with differentially used polyadenylation sites (Figures S2D–S2G).

To investigate the mechanism through which HuD promotes translation, we focused on translation factors identified as HuD targets by the CRAC analysis. Notably, many of these factors are known to be mTOR responsive (Hsieh et al., 2012; Larsson et al., 2012; Thoreen et al., 2012), including 5′-TOP or 5′-TOP-like mRNAs (Meyuhas and Kahan, 2015) (Figure 2G). Among these mRNAs, we selected for validation the translation elongation factor *Eef1a1*, the cytoplasmic poly(A) binding protein *Pabpc1*, and the eukaryotic initiation factors *Eif4a1* and *Eif4a2*. *Eef1a1*, *Pabpc1*, *Eif4a1*, and *Eif4a2* mRNAs are strongly bound by HuD in their 3′ UTRs (Figure S2C). As shown in Figure 2H, overexpression of HuD significantly increased the protein levels of Eef1a1, Pabpc1, Eif4a1, and Eif4a2 with respect to tubulin. As negative control we used the exon junction complex component Eif4a3, which is a recognized Eif4a1 and Eif4a2 paralog, but neither a HuD target nor a TOP gene. Levels of Eif4a3 were unaffected by enhanced HuD expression.

HuD Translation Enhancement Activity Does Not Depend on the mTORC1 Pathway

Since mTOR-responsive genes were significantly enriched among HuD targets (Figure 2G), we next assessed if the HuD-dependent boost to global and target specific translation was mediated through the mTORC1 pathway. We serum starved cells to decrease activity of the mTORC1 pathway to less than 50%, as assessed by the phosphorylation status Eif4ebp1 and Rps6. This treatment did not affect the levels of endogenous HuD or inducible His-HA-HuD (Figure 3A) and did not induce the formation of P-bodies or stress granules (Figure S3A) in NSC-34 cells. We then measured global TE by polysome profiling. As expected, starvation caused a decrease in the global TE compared to serum-repleted cells. Interestingly, HuD overexpression restored and even increased TE in serum-depleted cells relative to repleted cells (Figure 3B). HuD overexpression also efficiently suppressed the effects of the mTORC1 inhibitor Torin1 (Figure S3B). We selected different classes of mTOR-responsive, HuD-bound mRNAs for TE quantification by qPCR: ribosomal proteins, polyadenylation factors, translation elongation, and initiation factors. The results consistently proved that HuD overexpression increased the TE of these target mRNAs upon starvation (Figure 3C), whereas Eif4a3 was unaffected. We further verified that the increase in TE correlated with enhanced Eef1a1 protein levels, with no effect on the negative control Eif4a3 (Figure 3D).

These results indicate that increased HuD activity is able to rescue global and target-specific translation inhibition exerted by partial suppression of mTORC1 pathway signaling. To confirm this, we further explored how HuD regulates the expression of the 5′ TOP gene Eef1a1, known to be selectively modulated by mTORC1. We cloned the 3′ UTRs of *Eef1a1* and the

(E) Transcriptome-wide translation efficiency changes upon HuD overexpression in NSC-34 cells. Scatterplot displaying for each gene the average expression signal (CPM) against the log2 change in translation efficiency (delta TE) upon HuD overexpression. Genes with increased or decreased TE are highlighted.
(F) Enrichment analysis of HuD RNA targets among genes with increased or decreased TE upon HuD overexpression, compared to enrichments associated with genes changing at either the polysomal or the total RNA level. Fisher's test *p < 0.05, **p < 0.01, and ***p < 0.001.
(G) Enrichment of mTOR responsive mRNAs among HuD targets, as listed in multiple literature sources.
(H) Western blot analysis of HuD targets (Eef1a1, Eif4a1, Eif4a2, Pabpc1) and negative control (Eif4a3) in HEK293 cells transiently transfected with HuD. Tubulin was used as reference. Experiments were performed at least in triplicate.
In (C), (D), and (H), data are represented as mean ± SEM; t test *p < 0.05, **p < 0.01, and ***p < 0.001.
See also Figure S2.

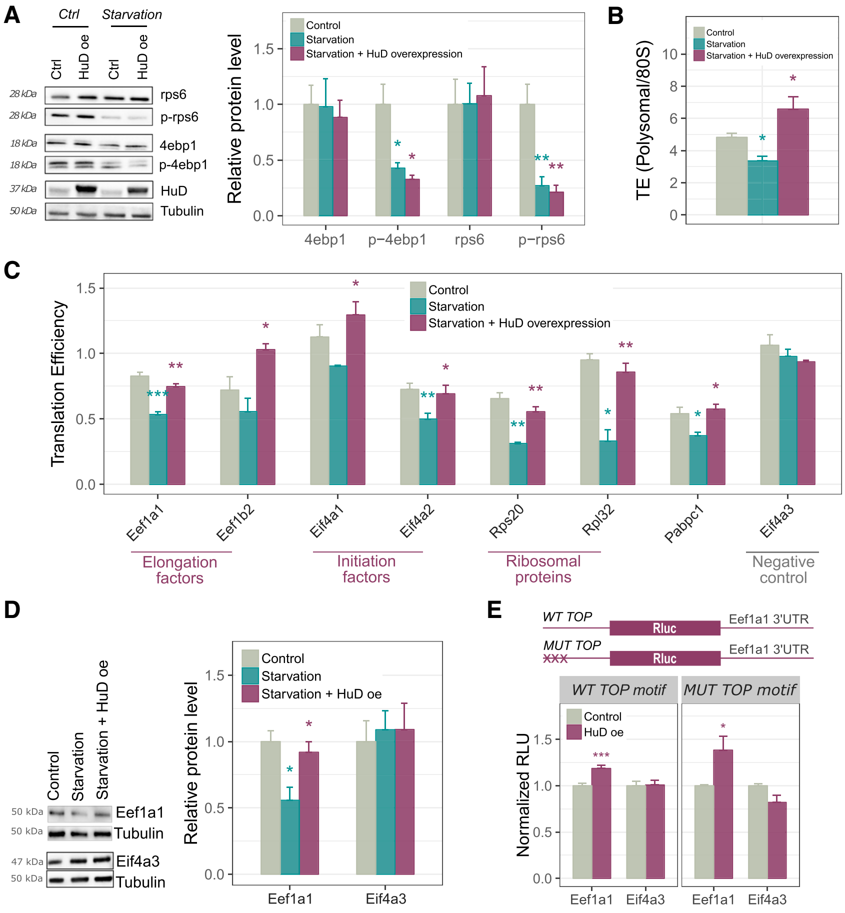

Figure 3. HuD Enhancement of Global and Target-Specific Translation Efficiency Does Not Depend on the mTORC1 Pathway

(A) Left: western blot analysis of Rps6 and Eif4ebp1 phosphorylation following serum deprivation (8 hr) in NSC-34 cells.

(B) Measurement of global TE by sucrose gradient centrifugation in the following conditions: control, starvation, and starvation coupled with HuD overexpression.

(C) TE quantification of selected mTOR-responsive mRNAs in control, starvation, and starvation coupled with HuD overexpression conditions. Target-specific TE is the ratio between polysomal and total RNA changes measured by RT-qPCR. Gapdh and Als2 were used as reference genes.

(D) Western blot analysis of Eef1a1 and Eif4a3 in NSC-34 cells collected in three different conditions: control, starvation, and starvation with HuD overexpression.

(E) Barplot displaying normalized luciferase intensity values in HEK293 cells transiently transfected with HuD, relative to transient transfection of the empty vector. Cells were co-transfected with wild-type (WT) or mutated (MUT) TOP motif bearing luciferase vectors with the 3′UTR of Eef1a1 (HuD target) or Eif4a3 (negative control).

In (A)–(E), data are represented as mean ± SEM t test *$p < 0.05$, **$p < 0.01$, and ***$p < 0.001$. In (A)–(C), “Starvation” was compared to “Control,” and “Starvation + HuD overexpression” was compared to “Starvation” for testing statistical significance. See also Figure S3.

negative control *Eif4a3* downstream of luciferase, in a reporter vector harboring a canonical TOP motif at the 5′ end (Thoreen et al., 2012). We expressed these reporters alone or in combination with HuD in HEK293 cells, not expressing endogenous HuD. Luciferase activity was enhanced by HuD co-expression in the case of the vector carrying the 3′ UTR from *Eef1a1*, but not for *Eif4a3* (Figure 3E). Importantly, we obtained the same results when using a luciferase vector with a mutated TOP motif not responding to mTOR signaling (MUT-TOP; Thoreen et al., 2012), in the 5′ UTR (Figure 3E). These results collectively demonstrate that the translational control exerted by the mTORC1 pathway on 5′ UTR TOP mRNAs can be independently tuned by the translational enhancement promoted by HuD through binding to the 3′ UTR.

HuD Stimulates the Translation of mRNAs Involved in Neuronal Fate Commitment and in Axonogenesis

Control of translation is a key step in mediating neuronal activity and synaptic plasticity. HuD was demonstrated to induce neuronal differentiation, acting on specific neuronal target mRNAs (Deschênes-Furry et al., 2007).

We identified as high-confidence hits multiple neuronal mRNAs previously reported to interact with HuD (Table S1). These included Gls, Ikzf5, Lmo4, Marcks, Msi1, Nova1, Nrn1, App, and Atg5 (Akten et al., 2011; Bronicki and Jasmin, 2013; Kang et al., 2014). Analysis of mRNAs responsible for neuronal specification in the CRAC data revealed enrichment for genes involved in neuronal differentiation and neurogenesis, and genes involved in axonogenesis, axon guidance, myelin deposition, axon localization, and synaptic functionality (Figure S3C). To assess whether HuD binding to these mRNAs results in phenotypic effects on neurogenesis, we induced HuD overexpression in differentiating NSC-34 cells. We observed a significant increase in neuronal outgrowth in HuD overexpressing cells compared to control cells (Figure S3D). We also confirmed that HuD promotes neurite extension in PC12 cells (Fukao et al., 2009), and that this ability is preserved by two HuD isoforms (HuD-sv1 and HuD-sv2), as reported by Hayashi et al. (2015) (Figure S3E).

Next, we inspected whether HuD expression correlated with enhanced TE for 11 selected HuD target mRNAs, known to play important roles in motor neurons and axons. As shown in Figure S4F, we found a significant TE increase in HuD overexpressing cells for each of these mRNAs. The increase was greater for *Kif5b*, *Sema4d*, *Picalm*, *Acsl4*, and *Hnrnpa2b1*. TE enhancement upon HuD overexpression was driven by increased polysomal occupancy, with almost no variation in total RNA levels. We then examined the overlap between HuD binding targets and mRNAs with altered expression in motor neuron diseases (Figure S3G). We observed a strong enrichment for motor

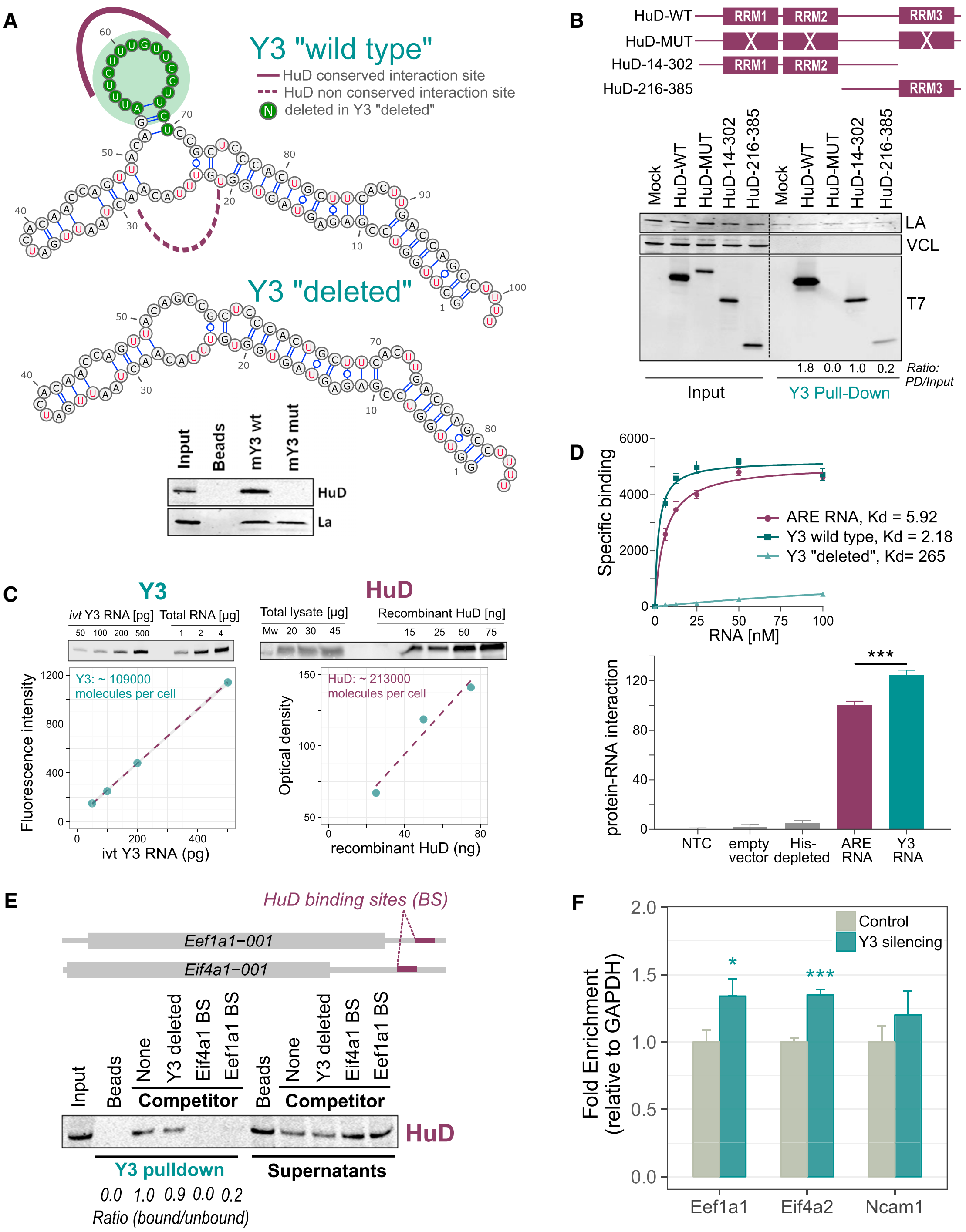

Figure 4. Y3 Competes for HuD Association with Target mRNAs

(A) Upper panel: secondary structure of Y3 with HuD interaction sites (visualized with VARNA) based on chemical probing. Center panel: representation of the Y3 “deleted” variant, obtained by eliminating the conserved HuD binding region. Lower panel: His-HA-HuD was induced in NSC-34 cells. Lysates were subjected to RNA pull-downs with biotinylated Y3, followed by immunoblot for HuD and La proteins. Either the wild-type Y3 sequence or the mutant that lacks the HuD binding site was used.

(B) Y3 RNA-pull-down showing that HuD interacts with Y3 by the RRM domains, mainly RRM1 and RRM2.

(C) Quantification of Y3 and HuD molecule number in NSC-34 cells. The estimated molecule number was calculated by means of a calibration plot generated by known amounts of standards, i.e., *in vitro*-transcribed (ivt) Y3 RNA and recombinant HuD, respectively.

(legend continued on next page)

neuron disease-associated genes among HuD targets, and we confirmed the effects of HuD overexpression on translation for specific genes associated with ALS and genes with altered expression in both ALS and SMA (Figure S3H). This observation highlights a potential role for HuD in modulating the expression of pathologically relevant transcripts in motor neurons.

Y3 Competes for HuD Binding against mRNAs

Quantitative analysis of the CRAC interactions clearly identified the 102 nt ncRNA Y3 as the largely dominant HuD target. Inspection of the CRAC deletion profiles revealed two binding sites in Y3 that map to loop regions closely positioned in the secondary structure (Teunissen et al., 2000) (Figures 4A and S4A). From an analysis of sequence evolutionary conservation, which confirmed previous literature (Farris et al., 1995), we found that the first Y3 HuD binding region (nt 20–25) is markedly less conserved than the second (nt 55–70). Based on this result, we generated a Y3 "deleted" variant by eliminating the conserved HuD binding region (Figure 4A). This variant is unable to interact with HuD, as assessed by RNA pull-down (Figure 4A, lower panel).

Similarly, we determined the region of HuD involved in Y3 binding. We transfected NSC-34 cells with four different HuD constructs (Fukao et al., 2009): (1) wild-type (WT); (2) HuD-MUT, lacking any RNA-binding activity; (3) HuD-14-302, lacking RRM3, the HuD RNA binding domain proposed to bind the poly(A); and (4) HuD-216-385, lacking the RNA binding domains RRM1 and RRM2. By Y3 pull-down, we found that the HuD RRM domains are necessary for the interaction with Y3, with a stronger contribution of the first and the second RRMs (Figure 4B).

After having established the molecular details of the HuD/Y3 interaction, we investigated their relative stoichiometry in cells. Using calibration curves, we estimated that NSC-34 cells contain on average approximately 213,000 molecules of HuD protein and 109,000 molecules of Y3 RNA (see STAR Methods; Figure 4C). If the two HuD binding sites on Y3 were occupied by different HuD molecules, this estimated ratio (1.95) would suggest that Y3 might be able to sequester much or all of the HuD population in standard NSC-34 culture conditions.

To quantitatively characterize the HuD/Y3 interaction, we applied a luminescence proximity assay optimized for protein/RNA interactions (D'Agostino et al., 2013). Biotinylated RNA probes, representing Y3, Y3 lacking the conserved HuD binding site (Y3-deleted) or a strong canonical 27 nt AU-rich sequence element (ARE), were incubated with the recombinant HuD protein. Saturation binding experiments demonstrated a stronger affinity for the Y3 RNA (Kd of 2.1 nM) as compared with the ARE RNA (Kd of 5.9 nM), with no appreciable binding of Y3-deleted (Figure 4D, upper panel). We confirmed these *in vitro* data with lysates from NSC-34 cells transfected with the HuD construct: HuD binding activity to the Y3 probe was enriched of about 30% with respect to the ARE RNA probe (Figure 4D, lower panel).

Due to both its high intracellular levels and its high binding affinity for HuD, Y3 might effectively compete with the HuD mRNA targets, acting as a specific HuD molecular decoy. To test this hypothesis, we evaluated whether Y3 could compete for HuD binding with some of the HuD mRNA targets we had previously validated. We selected two sequences from the 3′ UTRs of the target *Eif4a1* and *Eef1a1* mRNAs, containing HuD binding sites identified by CRAC and matching the size of the Y3 RNA (Figure 4E). Next, we measured the competition for HuD binding between the selected sequences and Y3. As shown in Figure 4E, while Y3-deleted is not able to compete for the HuD/Y3 interaction, the two selected mRNA target sites are able to compete. To confirm the action of Y3 in reducing HuD association with its target mRNAs, HuD was immunoprecipitated from NSC-34 cells with or without prior treatment with siRNAs directed against Y3, and three HuD-associated mRNAs (*Eef1a1*, *Eif4a2*, and *Ncam1*) were quantified. Cells depleted for Y3 showed increased co-precipitation with HuD for all the three targets (Figure 4F). We also verified that Y3 silencing did not affect the mRNA abundance of these targets (Figure S4B).

Finally, we tested whether HuD post-translational modifications such as methylation and phosphorylation could alter HuD binding to Y3 or to the ARE RNA probe, but we were unable to detect significant differences (Figures S4C and S4D).

Collectively, this body of results shows that the Y3 ncRNA sequesters HuD intracellularly and competes efficiently for HuD binding with its target mRNAs.

Y3 Counteracts the Translation Enhancement Activity of HuD

To assess the functional consequence of HuD sequestration by Y3, we tested whether Y3 modulates the translation enhancement ability exerted by HuD, by depleting Y3 in NSC-34 cells (Figure 5A). Measurement of the TE indicated increased ribosome engagement in active translation (Figure 5A), and this was supported by increased *de novo* protein synthesis following Y3 depletion (Figure 5B). These results indicate that Y3 acts as a general repressor of translation in NSC-34 cells.

To determine the relation between HuD and Y3 in the modulation of translation, we measured *de novo* protein synthesis after the following treatments: (1) HuD silencing by siRNA, (2) Y3 depletion by shRNA expression, and (3) combined silencing of HuD and Y3. Combined Y3 and HuD knockdown partially restored the reduction in protein synthesis observed following HuD knockdown alone (Figure 5C). These data indicate that

(D) Upper panel: saturation binding curves of recombinant HuD protein as function of increasing amount of RNA probes. Kd values were obtained by non-linear regression analysis. Three independent experiments were performed. Lower panel: AlphaScreen assay using ARE and Y3 RNA probes with lysates of NSC-34 cells expressing HuD protein. Two independent experiments were performed at the hooking point with 50 nM of RNA probes.

(E) HuD was induced in NSC-34 cells. Lysates were prepared and RNA pull-downs with biotinylated Y3 were conducted either without (none) or with competitor RNAs included in the extract (7× molar excess).

(F) RIP assay of HuD binding to Eef1a1, Eif4a2, and Ncam1 mRNAs after Y3 silencing; data were normalized to Gapdh mRNA levels in each IP.

In (D) and (F), data are represented as mean ± SEM t test *$p < 0.05$ and ***$p < 0.001$.

See also Figure S4.

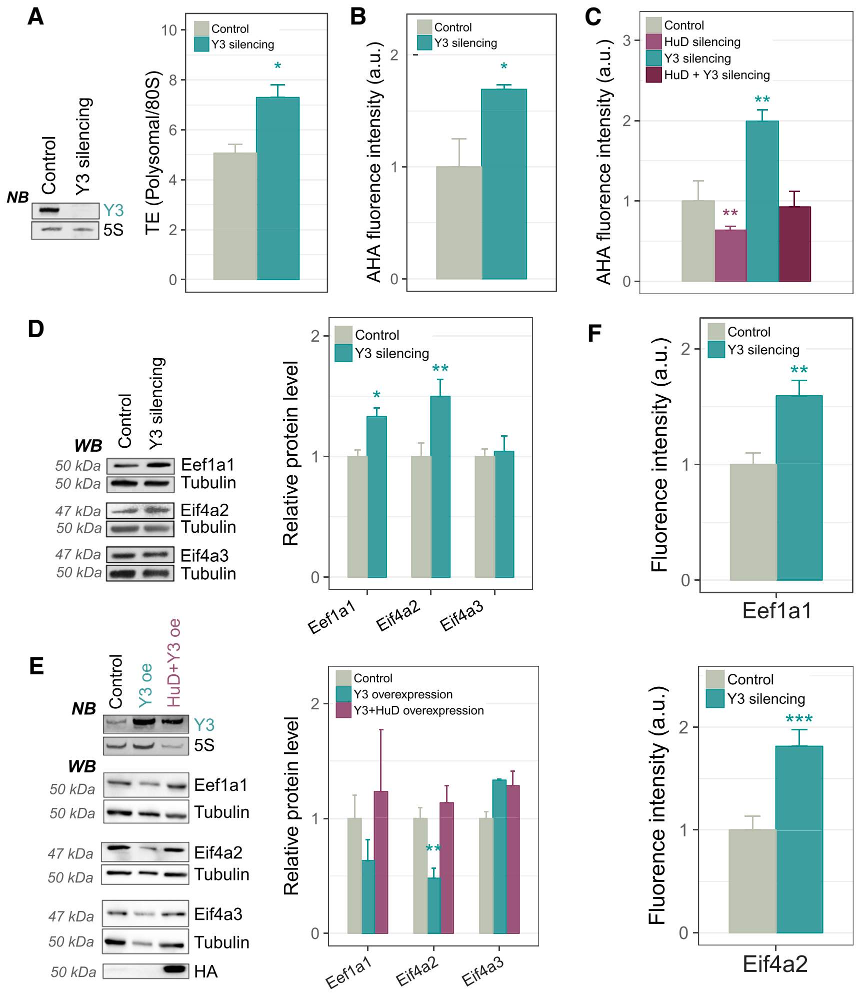

Figure 5. Y3 Modulates HuD Translation Functions

(A) Global translation output by sucrose gradient profiles upon Y3 silencing in NSC-34 cells.

(B) *De novo* protein synthesis by AHA labeling upon Y3 silencing in NSC-34 cells.

(C) AHA labeling experiments in NSC-34 cells depleted for Y3, for HuD, or for both, showing antagonism between Y3 and HuD on protein synthesis.

(D) Western blot of HuD targets (Eef1a1 and Eif4a2) and negative controls (Eif4a3) in NSC-34 cells transiently silenced for Y3. Experiments were performed at least in triplicate.

(E) Western blot analysis of EEF1A1, EIF4A2, and EIF4A3 levels in HEK293 cells transiently transfected with Y3 plasmid, alone or in combination with HuD vector. Experiments were performed at least in triplicate.

(F) Quantification of Eef1a1 and Eif4a2 protein levels in primary motor neurons transfected with an shRNA construct directed against Y3 (sh_Y3) or a control vector (sh_Ctrl) (n = >20 cells/condition).

In (A)–(F), data are represented as mean ±SEM t test *p < 0.05, **p < 0.01, and ***p < 0.001.

See also Figures S5 and S6.

the impact of HuD silencing on translation is mitigated if HuD sequestration by Y3 is also reduced, presumably due to an increase in the available pool of HuD (Figure 5C). To prove that the Y3 modulatory effect on global translation is mediated by the altered expression of HuD targets, we depleted Y3 in NSC-34 cells and assessed the protein levels of Eef1a1, Eif4a2 and the negative control Eif4a3. We observed a significant increase for both HuD targets, but not Eif4a3 (Figure 5D). We also tested the proposed molecular competition between Y3 and HuD on the translation of specific HuD targets by ectopic expression in HEK293 cells (Figure 5E). Overexpression of Y3 was associated with a specific decrease in the protein levels of Eef1a1 and Eif4a2, whereas no change was observed for Eif4a3. Co-expression of HuD restored protein expression of Eef1a1 and Eif4a2 to control levels.

To confirm that these effects are due to the HuD/Y3 interaction, we co-transfected HEK293 cells with HuD and wild-type Y3 RNA or Y3-deleted RNA. While overexpression of HuD combined with the Y3-deleted RNA leads to an increase of the Eef1a1 and Eif4a2 targets, the effect is reduced upon HuD overexpression together with the wild-type Y3 (Figure S5B). We further confirmed the ability of Y3 to prevent the translation of HuD target mRNAs using the human ovarian cancer cell line ES2. We took advantage of the fact that the interaction of the Y RNA with their major binding protein, the Ro60 autoantigen, is needed to avoid Y RNA degradation (Xue et al., 2003). We knocked out Ro60 by CRISPR/Cas9-based genome editing, obtaining two different Ro60-depleted cell clones. These clones had, as expected, much less Y3 RNA. Next, we rescued Ro60 expression in these clones. The ability of HuD to enhance the expression of its targets was conserved in ES2 cells, once more demonstrating that HuD exerts translational enhancement in neural cells. When Y3 was indirectly depleted by Ro60 knockout, the effect of HuD on translation was enhanced, and again reduced upon Ro60 rescue (Figure S5A).

We then verified if the functional interaction between HuD and Y3 was also present in mouse primary embryonic motor neurons (MNs). As previously reported (Fallini et al., 2011), HuD displays a distinctive granular pattern of localization in MNs (Figures S6A and S6B). Notably, primary MNs have high levels of endogenous Y3, mainly localized to the axonal compartment (Figures S6C and S6D). To test the effect of Y3 depletion on HuD targets, we performed transfection with either an shRNA vector targeting Y3 (shY3) or the empty control. We preliminarily tested the silencing efficiency of the shY3 vector (Figure S6E). Compared to control cells, shY3-treated MNs showed a significant increase in Eef1a1 and Eif4a2 protein levels, recapitulating the functional data obtained in NSC-34 cells (Figures 5F and S6F).

Collectively, these results show that Y3 counteracts the activity of HuD as a translation enhancer.

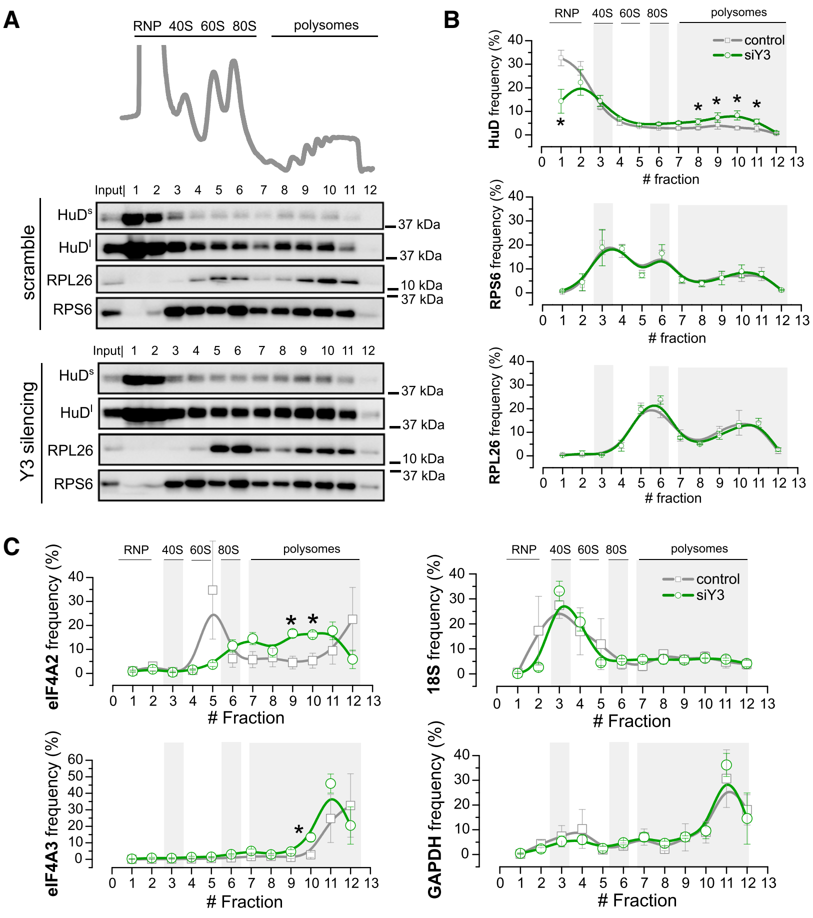

Figure 6. Y3 Reduces the Association with Polysomes of HuD and HuD mRNA Targets

(A) Example of sucrose gradient absorbance profile of NSC-34 cells treated with the scramble for siY3 (control cells, upper panel). The first peak contains free cytosolic light components (RNPs); the following peaks include the ribosomal subunits (40S and 60S) and not translating monosomes (80S). The peaks sedimenting at higher sucrose concentrations represent polysomes. In the lower panels, the co-sedimentation profiles of HuD (at short and long exposure time, HuDs and HuDl, respectively), RPL26 and RPS6 are shown under the corresponding sucrose gradient fractions for both the control (scramble, upper panels) and siY3 (lower panels).

(B) Semiquantitative analysis of HuD, RPL26, and RPS6 relative protein levels along the sucrose gradient fractions of control (gray lines) and siY3 (green lines) are shown as the mean values obtained from three independent experiments (n = 3).

(C) Semiquantitative analysis of Eif4a2, Eif4a3, 18S, and Gapdh relative transcript levels along the sucrose gradient fractions of control (gray lines) and Y3-depleted (green lines) cells are shown as the mean values obtained from three independent experiments (n = 3).

Data are represented as mean ± SEM t test *p < 0.05. See also Figure S7.

Y3 Sequesters HuD from the Polysomal Compartment

HuD can dynamically associate with polysomes (Bolognani et al., 2004). To determine whether Y3 can modulate HuD engagement on polysomes, we produced a co-sedimentation profile of HuD along an entire sucrose gradient in NSC-34 cells treated or not with Y3 siRNAs (Figure 6A). We found that HuD significantly moves from the subpolysomal RNP compartment to the polysomal one upon Y3 depletion (Figures 6A and 6B). Y3 silencing does not instead affect the localization of the ribosomal proteins RPS6 and RPL26, used as negative controls (Figures 6A and 6B). Moreover, we excluded the possibility that the enhanced association of HuD with polysomes was due to HuD increased expression after Y3 silencing, since HuD and Y3 do not mutually influence their abundance (Figures S7A–S7D). To corroborate these results at the level of single HuD target transcripts, we also monitored the changes in the localization of an HuD target mRNA along the sucrose gradient upon Y3 depletion. We chose Eif4a2 because it is one of the transcripts more heavily modulated by HuD and particularly affected by Y3 competition (Figure 6C), and because it is also the major form of eIF4A in neurons according to human expression databases and literature (Hornburg et al., 2014). Upon Y3 depletion, we observed a relevant increase in the *Eif4a2* mRNA polysomal localization (Figure 6C, top left panel), consistent with the increased polysomal localization of the HuD protein. Y3 silencing instead has minor or no effects on the localization of the *Eif4a3*, *18S*, and *Gapdh* RNAs, used as negative controls (Figure 6C).

These evidences, in combination with the specific localization of Y3 within the cytosolic RNP compartment and its absence from polysomes (Figure S7E), strongly support a role for Y3 in sequestering HuD away from polysomes and from its target mRNAs, preventing their translation.

Y3 Blocks the Function of HuD in Neuronal Differentiation

Given the established role of HuD in promoting neuronal differentiation during mammalian development, it seemed possible that a developmentally regulated switch in the HuD/Y3 ratio might control HuD availability for activity on mRNA targets, thus boosting neuronal differentiation in a specific temporal window. We analyzed changes in HuD and Y3 levels and ratio during neuronal development by converting mouse embryonic stem cells (mESCs) into neurons (Ying et al., 2003). We measured HuD and Y3 levels at three different stages of the differentiation procedure: mESC (D0), neural progenitors (D7), and early neurons (D10). We observed a progressive increase in levels of both Y3 and HuD during this process, but with different kinetics (Figure 7A). Y3 showed a substantial increase at the neural progenitor stage (2.5-fold at D7 relative to D0) but then showed only a modest further increase (3-fold at D10 relative to D0). In contrast, HuD exhibited a 5-fold increase at the neural progenitor stage (D7) and a 10-fold increase at the early neuron stage (D10). These results predict that a strong reduction in HuD sequestration by Y3 at the

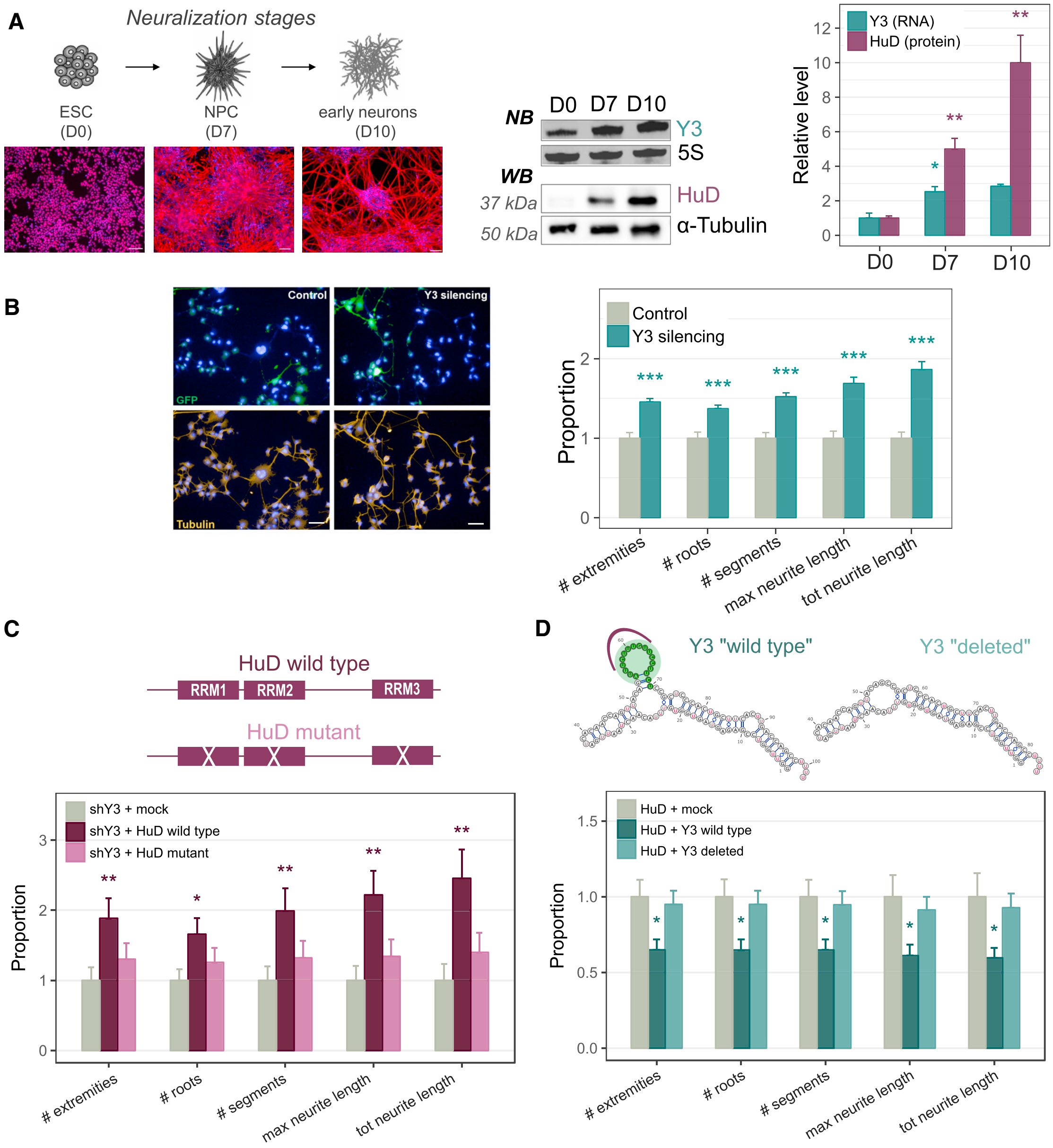

Figure 7. Y3 Counteracts HuD-Induced Neurogenesis

(A) Differentiating ESCs cultures assayed for Y3 and HuD expression levels by Northern blot and western blot, respectively. Cultures were immunostained for stage-specific markers: Oct4 (ESCs; red), Nestin (NPCs; red), and beta3-tubulin (early neurons; red); the scale bar corresponds to 75 μm. Relative quantification of Y3 and HuD levels are shown (right).

(B) Differentiated NSC-34 cells (control or silenced for Y3) immunostained with anti-tubulin antibody (yellow) to detect neurites (left panel); GFP (green) identified transfected cells subjected to high content analysis; the scale bar corresponds to 100 μm. Multiple parameters were analyzed using Operetta HCS device (right panel).

(C) Differentiation assay in control Y3 silenced cells, Y3 silenced cells transfected with wild-type HuD or with mutant HuD. A schematic representation of HuD constructs used in the experiment is provided.

(D) PC12 cells were co-transfected with HA-tagged HuD and mock or Y3 WT or Y3 "deleted" vectors. Co-transfected cells were immunostained with anti-HA antibody, and the neurites were stained for tubulin.

In (A)–(D), data are represented as mean ± SEM t test *p < 0.05, **p < 0.01 and ***p < 0.001.

neurogenic stage *in vivo* allows HuD to progressively drive neuronal differentiation.

To directly test for a negative role for Y3 in neuronal differentiation, we induced shY3 expression under differentiation conditions in NSC-34 cells. Y3 depletion significantly increased neurite outgrowth in comparison to control cells (Figure 7B). To demonstrate that this effect is specifically mediated by the HuD/Y3 interaction, we first transfected NSC-34 cells with either

the wild-type HuD construct or the mutated version unable to bind the Y3 RNA, after Y3 silencing and in differentiation conditions. As shown in Figure 7C, wild-type HuD enhanced neuronal differentiation in Y3-depleted cells, while mutant HuD lost this function. To further support this finding, we co-transfected the HuD vector with either wild-type Y3 or Y3-deleted vectors into PC12 cells upon nerve growth factor (NGF) stimulation. Overexpression of wild-type Y3 resulted in a reduced neurite extension in HuD transfected cells, while Y3-deleted, incapable of binding HuD, had no effect (Figure 7D).

These results show that Y3 effectively counteracts HuD-induced neuronal differentiation, and the increase of the HuD/Y3 ratio is a proposed way to trigger this program during differentiation.

DISCUSSION

The crucial role of HuD in motor neuron plasticity and axon regeneration (Akamatsu et al., 2005; Anderson et al., 2003; Deschênes-Furry et al., 2007) prompted us to set-up a method providing a nucleotide-resolution map of HuD binding in motor neuron-like cells. Our CRAC analysis showed that HuD is prevalently a 3′ UTR binding protein (92% of binding sites) in the coding transcriptome (Figure 2B). Functional analysis of the HuD interactome revealed, together with the strong neuronal differentiation signature, an unexpected functional enrichment related to translation. HuD resulted to bind up to 80 mRNAs of genes encoding for core components of the translational machinery (Figure 2A). The only available evidence of an action of HuD on global translation comes from (Fukao et al., 2009), demonstrating the binding of HuD to eIF4A1, which results in translation stimulation of a reporter luciferase mRNA in HeLa extracts. Interestingly, in their study the presence of the HuD binding site on the reporter construct does not influence translational stimulation, suggesting that indirect effects could be involved. We show for the first time a strong stimulation of HuD on global translation in motor neuron cells, assessed by increase in polysome formation and *de novo* protein synthesis (Figures 2C–2E). This global translation enhancement could be at least partially mediated by the direct effect of HuD on the elongation factors Eef1a1 and initiation factors Eif4a1 and Eif4a2 (Figure 2H). Increased availability of the helicase proteins and the induced HuD overexpression could favor the formation of more HuD/eIF4A complexes (Fukao et al., 2009, 2014), generating a positive feedback loop.

To our knowledge, such an extent of translational stimulation in mammalian cells is only possible by the engagement of the mTORC1 pathway, which mainly targets TOP and TOP-like mRNAs (Hsieh et al., 2012; Meyuhas and Kahan, 2015; Thoreen et al., 2012). Therefore, we checked the degree of coincidence between mTOR responsive genes and HuD targets, clearly demonstrating the high overlap among these lists (Figure 2G).

The mTORC1 pathway assures neuronal activity by promoting differentiation and synaptogenesis. Similarly to the HuD-induced phenotype in neurons (Figures S3C–S3E), the control of protein synthesis through mTORC1 is also essential for axonogenesis and dendritogenesis (Takei and Nawa, 2014). Therefore, we wondered if the newly found HuD control of global translation could act through stimulation of the mTORC1 pathway itself or instead follow an independent route. The multiple experiments we performed to resolve this issue (Figures 3A–3E) consistently favored the second possibility, showing that suppression of the mTORC1 translational burst can be rescued by HuD overexpression. Moreover, mRNAs respond to HuD with increased translation irrespective of the sequence at the 5′ end. We believe that this is the first demonstrated control of mTORC1-responsive mRNAs spatially segregated from the mRNA 5′ end. Indeed, while very recent evidences have indicated that the translation of TOP mRNAs is regulated to their 5′ terminal through the competitive binding between eIF4F, controlled by mTORC1 via 4E-BP proteins, and LARP1 (Philippe et al., 2018), we found that HuD exerts its function through the binding of the TOP or TOP-like mRNAs at the 3′ UTR.

These results can be interpreted in terms of a synthetic interaction in motor neurons between the mTORC1 pathway and HuD. We could hypothesize the existence of two independent and redundant triggers of the translational machinery, targeting two spatially segregated portions of the same mRNAs through a fail-safe mechanism to assure the correct translational output in highly polarized cells.

A second unexpected finding from our collection of HuD RNA interactions is the specific and extensive association with the Y3 RNA. Y RNAs are a conserved family of abundant small non-coding RNAs (ncRNA), 100 nt long on average. Although Y RNAs have been known for more than three decades, their cellular functions in vertebrates remain elusive. Using a pan-nELAV antiserum for CLIP analysis in human brain tissue, Scheckel et al. (2016) recently reported the first evidence of nELAV binding with 320 different Y sequences. So many different interactors are likely due to the existence of 1,000 Y retropseudogenes in the human genome (Perreault et al., 2005). The cumulative Y/nELAV binding increased in Alzheimer's disease brains and in UV-stressed neuroblastoma cells (Scheckel et al., 2016). Our data in murine motor neuron-like cells and with the specific nELAV HuD are instead in favor of a very specific interaction with the Y3 RNA, fitting the sequence consensus we found for HuD binding (Figures 4A and 4B). This high selectivity could have been favored also by the existence in the mouse genome of only 60 Y retropseudogenes, diverged in sequence from the two canonical Y RNA genes (Perreault et al., 2007).

Surprisingly, the extent of association between HuD and Y3 in our culture conditions is higher than the cumulative association of the other 1,304 coding and 130 non-coding RNAs. Considering our estimation of the number of HuD and Y3 molecules per cell (Figure 4C), in our conditions the majority of the expressed Y3 RNA could be associated to HuD. This is also supported by the higher affinity of HuD for Y3 with respect to a strong, ARE-containing HuD binding RNA (Figure 4D). This evidence is instrumental to the hypothesis that Y3 could efficiently modulate HuD in its function as translational enhancer. The subsequent set of experiments convinced us that Y3 negatively affects HuD translational activity by efficiently sequestering it from the translational compartment. In fact, Y3 is completely absent from polysomes and localizes within the cytosolic RNP compartment (Figure S7E). Consistently, Y3 silencing improved the association between HuD and its target mRNAs

(Figures 4E and 4F) and increased the polysomal localization of HuD (Figures 6A and 6B). On the functional side, Y3 depletion increased HuD ability to boost translation and effectively rescued HuD depletion (Figures 5A–C5). Similarly, HuD rescued transcript-specific translation when overexpressed in combination with Y3 (Figures 5E and S5).

Finally, we observed a variation of HuD/Y3 level ratio during neural mouse embryo stem cells differentiation (Figures 7A and 7B), and we demonstrated that neuronal differentiation can be specifically modulated by the HuD-Y3 interaction (Figures 7C and 7D). We therefore suggest that a developmentally regulated switch in the HuD/Y3 ratio *in vivo* may induce release of active HuD, thus boosting neuronal differentiation in a specific temporal window. Interestingly, we also report a localization enrichment of Y3 in primary motor neuron processes, mainly in the axons (Figure S6). HuD has been described to localize in axons and dendrites and to actively associate with polysomes upon depolarization (Tiruchinapalli et al., 2008). Therefore, the formation of a HuD/Y3 RNP could contribute to HuD silencing during neuritic transport, triggering translation in neuron microdomains following specific stimuli.

Our description of an efficient decoy activity on HuD function by Y3 suggests a new role for the Y ncRNAs, which could extend to other RBPs binding the loop region. The concept of competing endogenous RNAs (Tay et al., 2014) is well established, and applies mostly to microRNAs sequestered from target mRNAs. Functional sequestration of RBPs has been described for some lncRNAs such as cyrano, which sequesters the HuD paralog HuR (Kim et al., 2016), previously shown by us to associate to Y3 (Köhn et al., 2015). Functions of the ELAV RBPs could therefore be controlled by an extensive network of small and long ncRNAs in different cell types.

In conclusion, our work introduces a novel key function for HuD which could be exploited for therapeutic purposes. Limiting to motor neuron diseases, in SMA mice increased mTORC1 signaling by downregulation of its negative controller PTEN (Ning et al., 2010) rescues axonal defects and improves survival. For these reasons, attempts aimed at stimulating the mTORC1 pathway could have therapeutic potential for degenerating motor neurons. We report here a new activity of HuD as an mTORC1-independent global translational enhancer. This activity offers a window of therapeutic opportunity, which becomes even more interesting when considering the high modulation of HuD function exerted by the Y3 ncRNA.

STAR★METHODS

Detailed methods are provided in the online version of this paper and include the following:

- KEY RESOURCES TABLE
- CONTACT FOR REAGENT AND RESOURCE SHARING
- EXPERIMENTAL MODEL AND SUBJECT DETAILS
  - ○ Cell culture
- METHOD DETAILS
  - ○ Plasmids
  - ○ Generation of Tetracycline (Tet) inducible cell lines
  - ○ Isolation of motor neuron compartments
  - ○ Small interfering RNA (siRNAs) and cell transfections
  - ○ NSC-34 cell treatments
  - ○ Cell Differentiation
  - ○ Immunofluorescence microscopy
  - ○ Neurite outgrowth analysis
  - ○ CRAC
  - ○ Polysome profiling
  - ○ Extraction of total and polysomal RNA
  - ○ RT-qPCR analysis
  - ○ Library preparation for RNA-Seq and POL-Seq
  - ○ Library preparation for TruSeq Targeted RNA Expression
  - ○ RNP immunoprecipitation and RNA pulldown
  - ○ SBP Pulldown
  - ○ Northern and western blot
  - ○ AlphaScreen assays
  - ○ Quantification of HuD and Y3 molecules
  - ○ AHA assay
- QUANTIFICATION AND STATISTICAL ANALYSIS
  - ○ CRAC data analysis
  - ○ RNA-Seq and POL-Seq data analysis
  - ○ TruSeq Targeted RNA Expression data analysis
  - ○ Alternative polyadenylation data analysis
  - ○ Functional annotation enrichment analysis
  - ○ Statistical analysis
- DATA AND SOFTWARE AVAILABILITY

SUPPLEMENTAL INFORMATION

Supplemental Information includes seven figures and three tables and can be found with this article at https://doi.org/10.1016/j.molcel.2018.06.032.

ACKNOWLEDGMENTS

We thank Toshinobu Fujiwara for T7-HuD plasmids and Claudia Fallini for the EGFP-HuD vector. We acknowledge the following CIBIO core facilities for crucial support: High Throughput Screening, Next Generation Sequencing, Model Organism, Protein Technology, and Advanced Imaging. We thank Viktoryia Sidarovich for valuable suggestions, Paolo Struffi for the HuD recombinant protein, Alessandro Roncador for assisting with primary cultures, and Hendrik Täuber for experimental validations. This work was supported by Provincia Autonoma di Trento, Grande Progetto PAT 2012 AxonomiX, and by Fondazione Cassa di Risparmio di Trento e Rovereto, Convenzione UniTrento Fondazione Caritro 2016. In addition, we acknowledge financial support from the Wellcome Trust grant 109916 and the Deutsche Forschungsgemeinschaft-funded grant Hu1547/9-1, part of SPP1935.

AUTHOR CONTRIBUTIONS

T.T., P.Z., D.P., and A.Q. conceived the study. T.T., P.Z., D.P., M.K., L.G., G.V., V.D., and A.Q. designed the experiments. P.Z., D.P., M.K., L.G., V.P., V.B., T.D., G.V., A.R., and V.D. performed the experiments. T.T., P.Z., D.P., and G.S. analyzed the data. M.K., S.H., L.C., D.T., and P.M. discussed the results and edited the paper. T.T., P.Z., D.P., and A.Q. wrote the paper.

DECLARATION OF INTERESTS

The authors declare no competing interests.

Received: October 12, 2017
Revised: April 24, 2018
Accepted: June 21, 2018
Published: July 19, 2018

REFERENCES

Akamatsu, W., Fujihara, H., Mitsuhashi, T., Yano, M., Shibata, S., Hayakawa, Y., Okano, H.J., Sakakibara, S., Takano, H., Takano, T., et al. (2005). The RNA-binding protein HuD regulates neuronal cell identity and maturation. Proc. Natl. Acad. Sci. USA *102*, 4625–4630.

Akten, B., Kye, M.J., Hao, T., Wertz, M.H., Singh, S., Nie, D., Huang, J., Merianda, T.T., Twiss, J.L., Beattie, C.E., et al. (2011). Interaction of survival of motor neuron (SMN) and HuD proteins with mRNA cpg15 rescues motor neuron axonal deficits. Proc. Natl. Acad. Sci. USA *108*, 10337–10342.

Anderson, K.D., Merhege, M.A., Morin, M., Bolognani, F., and Perrone-Bizzozero, N.I. (2003). Increased expression and localization of the RNA-binding protein HuD and GAP-43 mRNA to cytoplasmic granules in DRG neurons during nerve regeneration. Exp. Neurol. *183*, 100–108.

Bernabò, P., Tebaldi, T., Groen, E.J.N., Lane, F.M., Perenthaler, E., Mattedi, F., Newbery, H.J., Zhou, H., Zuccotti, P., Potrich, V., et al. (2017). In vivo translatome profiling in spinal muscular atrophy reveals a role for SMN protein in ribosome biology. Cell Rep. *21*, 953–965.

Bolognani, F., Merhege, M.A., Twiss, J., and Perrone-Bizzozero, N.I. (2004). Dendritic localization of the RNA-binding protein HuD in hippocampal neurons: association with polysomes and upregulation during contextual learning. Neurosci. Lett. *371*, 152–157.

Bolognani, F., Contente-Cuomo, T., and Perrone-Bizzozero, N.I. (2010). Novel recognition motifs and biological functions of the RNA-binding protein HuD revealed by genome-wide identification of its targets. Nucleic Acids Res. *38*, 117–130.

Bronicki, L.M., and Jasmin, B.J. (2013). Emerging complexity of the HuD/ELAVl4 gene; implications for neuronal development, function, and dysfunction. RNA *19*, 1019–1037.

Conrad, R., Jablonka, S., Sczepan, T., Sendtner, M., Wiese, S., and Klausmeyer, A. (2011). Lectin-based isolation and culture of mouse embryonic motoneurons. J. Vis. Exp. *55*, 3200.

D'Agostino, V.G., Adami, V., and Provenzani, A. (2013). A novel high throughput biochemical assay to evaluate the HuR protein-RNA complex formation. PLoS ONE *8*, e72426.

Deschênes-Furry, J., Mousavi, K., Bolognani, F., Neve, R.L., Parks, R.J., Perrone-Bizzozero, N.I., and Jasmin, B.J. (2007). The RNA-binding protein HuD binds acetylcholinesterase mRNA in neurons and regulates its expression after axotomy. J. Neurosci. *27*, 665–675.

Fallini, C., Zhang, H., Su, Y., Silani, V., Singer, R.H., Rossoll, W., and Bassell, G.J. (2011). The survival of motor neuron (SMN) protein interacts with the mRNA-binding protein HuD and regulates localization of poly(A) mRNA in primary motor neuron axons. J. Neurosci. *31*, 3914–3925.

Fallini, C., Bassell, G.J., and Rossoll, W. (2012). The ALS disease protein TDP-43 is actively transported in motor neuron axons and regulates axon outgrowth. Hum. Mol. Genet. *21*, 3703–3718.

Farris, A.D., O'Brien, C.A., and Harley, J.B. (1995). Y3 is the most conserved small RNA component of Ro ribonucleoprotein complexes in vertebrate species. Gene *154*, 193–198.

Fukao, A., Sasano, Y., Imataka, H., Inoue, K., Sakamoto, H., Sonenberg, N., Thoma, C., and Fujiwara, T. (2009). The ELAV protein HuD stimulates cap-dependent translation in a Poly(A)- and eIF4A-dependent manner. Mol. Cell *36*, 1007–1017.

Fukao, A., Mishima, Y., Takizawa, N., Oka, S., Imataka, H., Pelletier, J., Sonenberg, N., Thoma, C., and Fujiwara, T. (2014). MicroRNAs trigger dissociation of eIF4AI and eIF4AII from target mRNAs in humans. Mol. Cell *56*, 79–89.

Granneman, S., Kudla, G., Petfalski, E., and Tollervey, D. (2009). Identification of protein binding sites on U3 snoRNA and pre-rRNA by UV cross-linking and high-throughput analysis of cDNAs. Proc. Natl. Acad. Sci. USA *106*, 9613–9618.

Hayashi, S., Yano, M., Igarashi, M., Okano, H.J., and Okano, H. (2015). Alternative role of HuD splicing variants in neuronal differentiation. J. Neurosci. Res. *93*, 399–409.

Helwak, A., Kudla, G., Dudnakova, T., and Tollervey, D. (2013). Mapping the human miRNA interactome by CLASH reveals frequent noncanonical binding. Cell *153*, 654–665.

Hornburg, D., Drepper, C., Butter, F., Meissner, F., Sendtner, M., and Mann, M. (2014). Deep proteomic evaluation of primary and cell line motoneuron disease models delineates major differences in neuronal characteristics. Mol. Cell. Proteomics *13*, 3410–3420.

Hsieh, A.C., Liu, Y., Edlind, M.P., Ingolia, N.T., Janes, M.R., Sher, A., Shi, E.Y., Stumpf, C.R., Christensen, C., Bonham, M.J., et al. (2012). The translational landscape of mTOR signalling steers cancer initiation and metastasis. Nature *485*, 55–61.

Kang, M.-J., Abdelmohsen, K., Hutchison, E.R., Mitchell, S.J., Grammatikakis, I., Guo, R., Noh, J.H., Martindale, J.L., Yang, X., Lee, E.K., et al. (2014). HuD regulates coding and noncoding RNA to induce APP→Aβ processing. Cell Rep. *7*, 1401–1409.

Kim, J., Abdelmohsen, K., Yang, X., De, S., Grammatikakis, I., Noh, J.H., and Gorospe, M. (2016). LncRNA *OIP5-AS1/cyrano* sponges RNA-binding protein HuR. Nucleic Acids Res. *44*, 2378–2392.

Köhn, M., Pazaitis, N., and Hüttelmaier, S. (2013). Why YRNAs? About versatile RNAs and their functions. Biomolecules *3*, 143–156.

Köhn, M., Ihling, C., Sinz, A., Krohn, K., and Hüttelmaier, S. (2015). The Y3** ncRNA promotes the 3′ end processing of histone mRNAs. Genes Dev. *29*, 1998–2003.

Kowalski, M.P., and Krude, T. (2015). Functional roles of non-coding Y RNAs. Int. J. Biochem. Cell Biol. *66*, 20–29.

Larsson, O., Morita, M., Topisirovic, I., Alain, T., Blouin, M.-J., Pollak, M., and Sonenberg, N. (2012). Distinct perturbation of the translatome by the antidiabetic drug metformin. Proc. Natl. Acad. Sci. USA *109*, 8977–8982.

Meyuhas, O., and Kahan, T. (2015). The race to decipher the top secrets of TOP mRNAs. Biochim. Biophys. Acta *1849*, 801–811.

Ning, K., Drepper, C., Valori, C.F., Ahsan, M., Wyles, M., Higginbottom, A., Herrmann, T., Shaw, P., Azzouz, M., and Sendtner, M. (2010). PTEN depletion rescues axonal growth defect and improves survival in SMN-deficient motor neurons. Hum. Mol. Genet. *19*, 3159–3168.

Pascale, A., Amadio, M., and Quattrone, A. (2008). Defining a neuron: neuronal ELAV proteins. Cell. Mol. Life Sci. *65*, 128–140.

Perreault, J., Noël, J.-F., Brière, F., Cousineau, B., Lucier, J.-F., Perreault, J.-P., and Boire, G. (2005). Retropseudogenes derived from the human Ro/SS-A autoantigen-associated hY RNAs. Nucleic Acids Res. *33*, 2032–2041.

Perreault, J., Perreault, J.-P., and Boire, G. (2007). Ro-associated Y RNAs in metazoans: evolution and diversification. Mol. Biol. Evol. *24*, 1678–1689.

Perrone-Bizzozero, N., and Bolognani, F. (2002). Role of HuD and other RNA-binding proteins in neural development and plasticity. J. Neurosci. Res. *68*, 121–126.

Philippe, L., Vasseur, J.-J., Debart, F., and Thoreen, C.C. (2018). La-related protein 1 (LARP1) repression of TOP mRNA translation is mediated through its cap-binding domain and controlled by an adjacent regulatory region. Nucleic Acids Res. *46*, 1457–1469.

Sanna, M.D., Peroni, D., Quattrone, A., Ghelardini, C., and Galeotti, N. (2015). Spinal RyR2 pathway regulated by the RNA-binding protein HuD induces pain hypersensitivity in antiretroviral neuropathy. Exp. Neurol. *267*, 53–63.

Scheckel, C., Drapeau, E., Frias, M.A., Park, C.Y., Fak, J., Zucker-Scharff, I., Kou, Y., Haroutunian, V., Ma'ayan, A., Buxbaum, J.D., and Darnell, R.B. (2016). Regulatory consequences of neuronal ELAV-like protein binding to coding and non-coding RNAs in human brain. eLife *5*, e10421.

Takei, N., and Nawa, H. (2014). mTOR signaling and its roles in normal and abnormal brain development. Front. Mol. Neurosci. *7*, 28.

Tay, Y., Rinn, J., and Pandolfi, P.P. (2014). The multilayered complexity of ceRNA crosstalk and competition. Nature *505*, 344–352.

Teunissen, S.W., Kruithof, M.J., Farris, A.D., Harley, J.B., Venrooij, W.J., and Pruijn, G.J. (2000). Conserved features of Y RNAs: a comparison of experimentally derived secondary structures. Nucleic Acids Res. *28*, 610–619.

Thoreen, C.C., Chantranupong, L., Keys, H.R., Wang, T., Gray, N.S., and Sabatini, D.M. (2012). A unifying model for mTORC1-mediated regulation of mRNA translation. Nature *485*, 109–113.

Tiruchinapalli, D.M., Caron, M.G., and Keene, J.D. (2008). Activity-dependent expression of ELAV/Hu RBPs and neuronal mRNAs in seizure and cocaine brain. J. Neurochem. *107*, 1529–1543.

Wang, X., and Tanaka Hall, T.M. (2001). Structural basis for recognition of AU-rich element RNA by the HuD protein. Nat. Struct. Biol. *8*, 141–145.

Xue, D., Shi, H., Smith, J.D., Chen, X., Noe, D.A., Cedervall, T., Yang, D.D., Eynon, E., Brash, D.E., Kashgarian, M., et al. (2003). A lupus-like syndrome develops in mice lacking the Ro 60-kDa protein, a major lupus autoantigen. Proc. Natl. Acad. Sci. USA *100*, 7503–7508.

Ying, Q.-L., Stavridis, M., Griffiths, D., Li, M., and Smith, A. (2003). Conversion of embryonic stem cells into neuroectodermal precursors in adherent monoculture. Nat. Biotechnol. *21*, 183–186.

STAR★METHODS

KEY RESOURCES TABLE

REAGENT or RESOURCE	SOURCE	IDENTIFIER
Antibodies		
Mouse monoclonal anti-MAP2	Sigma Aldrich	Catalog number M4403; RRID: AB_477193
Rabbit polyclonal anti-TAU	Synaptic System	Catalog number 314 002; RRID: AB_993042
Mouse monoclonal anti-SMI32	Abcam	Catalog number ab7795; RRID: AB_306084
Rabbit polyclonal anti-MNX1 (HB9)	Merck Millipore	Catalog number ABN174; RRID: AB_2732012
Mouse monoclonal anti-HUD (E-1)	Santa Cruz Biotechnology	Catalog number sc-28299; RRID: AB_627765
Mouse monoclonal Anti-β-Tubulin III	Sigma Aldrich	Catalog number T8578; RRID: AB_1841228
Mouse monoclonal anti-eEF1A1, clone CBP-KK1	Merck Millipore	Catalog number 05-235; RRID: AB_309663
Rabbit polyclonal anti eIF4A2	Abcam	Catalog number ab31218; RRID: AB_732123
Rabbit polyclonal anti-PABP	Abcam	Catalog number ab21060; RRID: AB_777008
Mouse monoclonal anti-DCP1A	Abcam	Catalog number ab57654; RRID: AB_942144
Mouse monoclonal Anti-TIA-1	Santa Cruz Biotechnology	Catalog number sc-166247; RRID: AB_2201545
Mouse monoclonal anti-Oct4 (C-10)	Santa Cruz Biotechnology	Catalog number sc-5279; RRID: AB_628051
Mouse monoclonal anti-Nestin, (clone rat-401)	Merck Millipore	Catalog number MAB353; RRID: AB_94911
Mouse monoclonal anti-β-Tubulin III	Promega	Catalog number G712A
Goat anti-Rabbit IgG (H+L) polyclonal, Cross-Adsorbed Secondary Antibody, Alexa Fluor 488	Thermo Fisher Scientific	Catalog number A-11008; RRID: AB_143165
Goat anti-Rabbit IgG (H+L) polyclonal, Cross-Adsorbed Secondary Antibody, Alexa Fluor 594	Thermo Fisher Scientific	Catalog number A-11012; RRID: AB_2534079
F(ab)2-Goat anti-Mouse IgG (H+L) polyclonal, Cross-Adsorbed Secondary Antibody, Alexa Fluor 488	Thermo Fisher Scientific	Catalog number A-11017; RRID: AB_2534084
F(ab)2-Goat anti-Mouse IgG (H+L) polyclonal, Cross-Adsorbed Secondary Antibody, Alexa Fluor 594	Thermo Fisher Scientific	Catalog number A-11020; RRID: AB_2534087
Donkey anti-Rabbit IgG (H+L) polyclonal, Highly Cross-Adsorbed Secondary Antibody, Alexa Fluor 488	Thermo Fisher Scientific	Catalog number A-21206; RRID: AB_2535792
Donkey anti-Goat IgG (H+L) polyclonal, preadsorbed Secondary Antibody, Alexa Fluor 594	Abcam	Catalog number ab150136
mouse monoclonal anti-β-tubulin (3F3-G2)	Santa Cruz Biotechnology	Catalog number sc-53140; RRID: AB_793543
Rabbit polyclonal anti-HA	Bethyl laboratories	Catalog number A190-108A; RRID: AB_67465
Rabbit polyclonal anti-eIF4A1	Abcam	Catalog number ab31217; RRID: AB_732122
Rabbit anti-eIF4A3	Home made by Prof. Macchi's Lab	
Rabbit polyclonal anti-eEF1A1	Sigma Aldrich	Catalog number SAB2108050

(*Continued on next page*)

Continued

REAGENT or RESOURCE	SOURCE	IDENTIFIER
Rabbit polyclonal anti-PABPC1	Sigma Aldrich	Catalog number SAB2101708; RRID: AB_10604467
Rabbit polyclonal anti-Rpl26	Abcam	Catalog number ab59567; RRID: AB_945306
Rabbit monoclonal anti-S6	Cell Signaling Technology	Catalog number 2217; RRID: AB_331355
Bacterial and Virus Strains		
XL1 Blue	Stratagene	Catalog number 200249
DH5alpha	This study	N/A
Chemicals, Peptides, and Recombinant Proteins		
Doxycycline	Sigma-Aldrich	Catalog number A3656
Torin1	EMD MILLIPORE	Catalog number 475991
Sodium arsenite solution	EMD MILLIPORE	Catalog number 1.06277
Cycloheximide (CHX)	Sigma-Aldrich	Catalog number C7698
Phorbol 12-myristate 13-acetate (PMA)	Sigma-Aldrich	Catalog number P8139
Dimethyl Sulfoxide (DMSO)	Fisher Scientific	Catalog number BP2311
NGF	Sigma-Aldrich	Catalog number N6009
GDNF	Peprotec	Catalog number 450-44-10
CNTF	Peprotec	Catalog number 450-13-10
BDNF	Peprotec	Catalog number 450-02-10
Collagen type IV	Sigma-Aldrich	Catalog number C5533
Laminin Mouse Protein, Natural	Thermo Fisher Scientific	Catalog number 23017015
Lectin Sigma L9640	Sigma-Aldrich	Catalog number L9640
Poly-DL-ornithine hydrobromide	Sigma-Aldrich	Catalog number P8638
Recombinant His-HuD protein	This study	N/A
Critical Commercial Assays		
Pierce Anti-HA Magnetic Beads	Thermo Fisher Scientific	Catalog number 88836
IBA Lifesciences Ni-NTA Superflow	Fisher Scientific	Catalog number 2-3206-025
Pierce Anti-HA Agarose Beads	Thermo Fisher Scientific	Catalog number 26181
Streptavidin MyOne T1 beads	Thermo Fisher Scientific	Catalog number 65601
ECL Prime Western Blotting System GE Healthcare	Sigma-Aldrich	Catalog number GERPN2232
Bradford Reagent	Sigma-Aldrich	Catalog number B6916
Lipofectamine RNAiMAX Reagent	Thermo Fisher Scientific	Catalog number 13778030
Lipofectamine 2000	Thermo Fisher Scientific	Catalog number 11668027
Dual-Glo Luciferase Assay System	Promega	Catalog number E2920
Retinoic acid	Sigma-Aldrich	Catalog number R2625
Click-iT AHA Alexa Fluor Protein Synthesis HCS Assay	Thermo Fisher Scientific	Catalog number C10289
Hoechst 33342	Thermo Fisher Scientific	Catalog number 62249
Starting Kit: Magnetic Plate + NeuroMag 200 µL	OZ Bioscience	Catalog number KC30800
AlphaScreen HA (Hemagglutinin) Detection Kit	PerkinElmer	Catalog number 6760612C
TruSeq Stranded mRNA Library Prep	Illumina	Catalog number 20020594
TruSeq Targeted RNA Custom Panel Kit	Illumina	Catalog number RT-101-1001
QuantSeq 3′ mRNA-Seq Library Prep Kit REV	Lexogen	Catalog number 016.24
iScriptcDNA synthesis kit	Biorad	Catalog number 1708891
KAPA SYBR FAST Universal 2X qPCR Master Mix	Kapa Biosystems	Catalog number KK4601 – 07959389001

(*Continued on next page*)

Continued

REAGENT or RESOURCE	SOURCE	IDENTIFIER
Deposited Data		
Raw Imaging files	This study, Mendeley Data	https://doi.org/10.17632/p34w7w78hy.1
Sequence files	This study, GEO GSE115490	https://www.ncbi.nlm.nih.gov/geo/query/acc.cgi?acc=GSE115490
Reference mouse genome annotation Gencode M6	Gencode	https://www.gencodegenes.org/mouse_releases/6.html
Experimental Models: Cell Lines		
H. sapiens: HEK293T	Quattrone A. Lab (CIBIO)	RRID: CVCL_0045
M. musculus: NSC34	Tebu-bio	RRID: CVCL_D356
M. musculus: NSC-34-Trex	This study	N/A
M. musculus: NSC-34-HuD	This study	N/A
M. musculus: NSC-34-shHuD	This study	N/A
R. norvegicus: PC12	Quattrone A.Lab (CIBIO)	RRID: CVCL_0481
M. musculus: 46C ES	Conti L. Lab (CIBIO)	RRID: CVCL_Y482
H. sapiens: CRISPR Knockout Ro60 ES2, Clone 1	Huettelmaier S. Lab	N/A
H. sapiens: CRISPR Knockout Ro60 ES2, Clone 1	Huettelmaier S. Lab	N/A
Experimental Models: Organisms/Strains		
C57BL/6J mice	The Jackson Laboratory	Catalog number 000664; RRID: IMSR_JAX:000664
Oligonucleotides		
See Table S2 for complete list of primers used for qPCR analysis and barcodes used for CRAC	This study	N/A
Y3 siRNA AACUAAUUGAUCACAACCAGU	Köhn et al., 2015	N/A
Ctrl siRNA AGGUAGUGUAAUCGCCUUG	This study	N/A
HuD siRNA	Santa Cruz Biotechnology	Catalog number sc-37836
Control siRNA	Santa Cruz Biotechnology	Catalog number sc-37007
Y1 Northern Blot probe, ATAACTCACTACCTTCGGA CCAGCC	Köhn et al., 2015	N/A
Y3 Northern Blot probe, CTGTAACTGGTTGTGATCA ATTAGT	Köhn et al., 2015	N/A
Biotinylated ARE RNA AUUAUUUAUUAUUUAUUUA UUAUUUA	This study	N/A
Biotinylated mY1 RNA, GGCTGGTCCGAAGGTAGTG AGTTATCTCAATTGATTGTTCACAGTCAGTTACAGAT TGAACTCCTGTTCTACACTTTCCCCCCTTCTCACTA CTGCACTTGACTAGTCTTTT	Köhn et al., 2015	N/A
Biotinylated mY3 RNA, GGTTGGTCCGAGAGTAGTG GTGTTTACAACTAATTGATCACAACCAGTTACAGAT TTCTTTGTTCCTTCTCCGCTCCCACTGCTTCACTT GACCAGCCTTTT	Köhn et al., 2015	N/A
Biotinylated hY4 RNA, GGCTGGTCCGATGGTAGTG GGTTATCAGAACTTATTAACATTAGTGTCACTAAAG TTGGTATACAACCCCCCACTGCTAAATTTGACTG GCTTTTT	Köhn et al., 2015	N/A
Recombinant DNA		
pCMV6-AN-His-HA	Origene	Catalog number PS100017
pCMV6-His-HA-HuD	This study	N/A
pCMV6-His-HA-HuD (R248K)	This study	N/A
pLenti CMV/TO His-HA-HuD	This study	N/A
pGEM-T-Y3wt	Köhn et al., 2015	N/A
pGEM-T-Y3mut (mutant lacking the HuD binding motif)	This study	N/A

(*Continued on next page*)

CellPress

Continued

REAGENT or RESOURCE	SOURCE	IDENTIFIER
pT7-HuD-WT	Fukao et al., 2009	N/A
pT7-HuD-MUT(mutant lacking any RNA-binding activity	Fukao et al., 2009	N/A
pT7-HuD-14-302	Fukao et al., 2009	N/A
pT7-HuD-216-385	Fukao et al., 2009	N/A
pIS*1*-Eef25UTR-TOPwt	Thoreen et al., 2012, A	Addgene Plasmid, Catalog number 38235
pIS1-Eef25UTR-TOPmut	Thoreen et al., 2012, A	Addgene Plasmid, Catalog number 38236
pIS*1*-Eef25UTR-TOPwt-3′UTR Eef1a1	This study	N/A
pIS1-Eef25UTR-TOPmut-3′UTR Eif4a3	This study	N/A
pIS*1*-Eef25UTR-TOPwt-3′UTR Eef1a1	This study	N/A
pIS1-Eef25UTR-TOPmut-3′UTR Eif4a3	This study	N/A
pHuD-GFP vector	Fallini et al., 2011	N/A
pshHuD	This study	N/A
pshY3	This study	N/A
pCDNA-SBP-HuD	This study	N/A
Software and Algorithms		
Prism	GraphPad, v5	https://www.graphpad.com/
Harmony software version 4.1	PerkinElmer	N/A
ImageJ software version 1.43u	NIH	https://imagej.nih.gov/ij/
Microscope Software Zen 2012 (Blue Edition)	Zeiss	https://www.zeiss.com/
Adobe Photoshop 7.0	Adobe Systems Incorporated	https://www.adobe.com/products/photoshop.html
hyb	https://github.com/gkudla/hyb	N/A
Tophat (version 2.0.14)	http://ccb.jhu.edu/software/tophat/index.shtml	N/A
R	https://www.r-project.org/	N/A
STAR (version 2.5.3a)	https://github.com/alexdobin/STAR	N/A
Bioconductor	https://www.bioconductor.org/	N/A
enrichR	http://amp.pharm.mssm.edu/Enrichr/	N/A
Other		
Stratalinker UV crosslinker 1800	Stratagene	N/A
UA-6 UV/VIS detector	Teledyne Isco	N/A
High Content Screening System Operetta	PerkinElmer	N/A

CONTACT FOR REAGENT AND RESOURCE SHARING

Further information and requests for resources and reagents should be directed to and will be fulfilled by the Lead Contact, Alessandro Quattrone (alessandro.quattrone@unitn.it).

EXPERIMENTAL MODEL AND SUBJECT DETAILS

Cell culture

NSC-34 is a murine hybrid cell line produced by fusion of mouse neuroblastoma cells with motoneuron-enriched embryonic spinal cord cells. NSC-34 cells were grown in DMEM medium with 10% FBS, 100 U/ml penicillin streptomycin and 0.01 mM L-glutamine (all medium ingredients were obtained from GIBCO). Human embryonic kidney HEK293 and human carcinoma (ES-2) cell lines were cultured in the same media and conditions. PC12 cells were cultured in Dulbecco's modified Eagle's medium supplemented with 10% fetal bovine serum and 5% horse serum, 100 U/ml penicillin streptomycin and 0.01 mM L-glutamine.

Mouse 46C ESCs (Ying et al., 2003) were maintained in Glasgow Minimal Essential medium (Sigma) supplemented with 10% heat-inactivated fetal bovine serum (EuroClone), 100 μM non-essential amino acids (Thermo Fisher), 1 mM sodium pyruvate (Thermo Fisher), 2 mM L-glutamine (Thermo Fisher), 100 U ml−1 penicillin (EuroClone), 100 μg ml−1 streptomycin (EuroClone), 1 mM β-mercaptoethanol (Thermo Fisher) and 1,000 U ml−1 murine leukemia inhibitor factor (ESGRO, Millipore) in gelatinized tissue culture flasks. Cells were passaged every 2-3 days after dissociation with 0.05% trypsin-EDTA (Thermo Fisher).

Primary motor neurons were isolated from embryonic mouse spinal cord. Lumbar spinal cord tissues were carefully dissected under microscopy, dissociated in trypsin and transfer in a lectin-coated plate. Lectin has been shown to specifically bind to $p75^{NTR}$ helping motorneurons enrichment. After washing, the cells were resuspended in neurobasal medium supplemented with 1% GlutaMAX, 2% B27 supplement, 5% horse serum and neurotrophic factors (BDNF, GDNF and CTNF at 10 ng/ml), and plated on PORN-H/laminin-coated plates. These mice-related activities were authorized by the Institutional Review Board of the University of Trento.

All cultures were grown at 37°C in a 5% CO_2 incubator.

METHOD DETAILS

Plasmids

To generate pCMV6-HIS-HA-HuD plasmid, the cDNA sequence of human HuD was amplified from SK-N-BE(2) neuroblastoma cell line using the following primers containing Sgf I and Mlu I restriction sites:

Fw HuD 5′-GAGGCGATCGCCGAGCCTCAGGTGTCAAATGG-3′

Rv HuD 5′-GCGACGCGTTCAGGACTTGTGGGCTTTGTTGG-3′

The amplified fragment was digested with Sgfi and MluI enzymes and cloned into the same sites of pCMV6-AN-His-HA vector, that contains an amino-terminal polyhistidine (His) tag and an hemagglutinin (HA) epitope (PS100017, OriGene, Rockville, MD). Site-directed mutagenesis was used to create an "unmethylatable" form of HuD. Briefly, pHA-HuD vector was used as PCR-template to generate a mutant of HuD, replacing the arginine at position 248 with a lysine (R248K). The primers containing the mutation are listed as follows:

R248K: (F) 5′-CCACCAGGCTCAGAAGTTCAGGCTGGACA-3′ and

(R) 5′-TGTCCAGCCTGAACTTCTGAGCCTGGTGG-3′;

To generate a lentiviral vector expressing tagged HuD, His-Ha-HuD was excised from pCMV6-AN-His-HA using BamHI and XhoI enzymes and subcloned in the same sites of pENTR-DsRed2 N1 (CMB1) vector. This plasmid was then recombined into pLenti CMV/TO Puro DEST (670-1, Addgene) destination vector using the Gateway system (Life technologies).

For HuD knockdown, the following oligonucleotides were synthesized and annealed:

5′-GATCCCGCATCCTGGTTGATCAAGTGTGTGCTGTCCACTTGATCAACCAGGATGCTTTTTGGAAA-3′;

5′-AGCTTTTCCAAAAAGCATCCTGGTTGATCAAGTGGACAGCACACACTTGATCAACCAGGATGCGG-3′.

Annealed fragments were ligated into the BglII and HindIII sites of pENTR/pSUPER+ (Addgene 575-1) and transferred into pCMV-GFP-DEST (Addgene 736-1), taking advantage of Gateway technology.

For knockdowns of Y3 by shRNAs, the following oligonucleotides were used:

5′-GATCCCCAACtAAttGAtCACAACCAGtTTCAAGAGAACTGGTTGTGATCAATTAGTTTTTTC-3′

5′-TCGAGAAAAAACTAATTGATCACAACCAGTTCTCTTGAAaCTGGTTGTGaTCaaTTaGTTGGG-3′. Annealed primers were ligated into pSuperior-GFP (OligoEngine), which was cut with BglII/XhoI. The empty vector served as negative control.

For Y3 overexpression, a pGEM-T clone including the whole Y3 gene (Köhn et al., 2015) was used. The sequence of Y3 mutant, lacking the HuD binding motif (AUUUCUUUGUUCCUUCU), was derived from CRAC data analysis, synthesized and cloned into pGEM-T vector.

To characterize Y3 binding with HuD, the following plasmids, kindly provided by Dr. Toshinobu Fujiwara, were used: pHuD-wt expressing murine HuD wild-type (wt), HuD-MUT vector lacking any RNA-binding activity, the HuD-14-302 lacking the poly(A)-binding domain RRM3 and theHuD-216-385 lacking the ARE-binding domain (RRM1 and RRM2).

The luciferase reporter vectors were generated by cloning the specific 3′UTR sequences into pIS1-Eef25UTR-renilla vector (Addgene 38235), that harbors a canonic TOP motif in 5′UTR. Specifically, the 3′UTR of Eef1a1, Eif4a1, Eif4a2, Eif4a3 and Rpl10 were amplified from murine cDNA by using the following primers:

Eef1a1 Fw 5′-GCACGGATATCATATTACCCCTAACACCTGC-3′

Rv 5′-GCACGTCTAGACAGATTTCTCATTAAACTTG-3′;

Eif4a1 Fw 5′-GCACGGATATCGGGGCTGTCCTGCGACCTGGCC-3′

Rv 5′-GCACGTCTAGAAGGCAGTTTCCAAGTAATTTTA-3′;

Eif4a2 Fw 5′-GCACGGATATCGGATGAGATAGTTTTGAATGC-3′

Rv5′-GCACGTCTAGACTTCATTAAGACATGTGCAAT-3′;

Eif4a3 Fw 5′-GCACGGATATCAGCTGGTGCTGGTGCACCGAG-3′

Rv 5′-GCACGTCTAGATCACAGGAAAATGTCCACGTT-3′;

Rpl10a Fw 5′-TTTTTGATATCCACGTGAAGATGACCGATGAT-3′

Rv 5′-TTTTTTCTAGAGAGTGGCAGCAGTGAGGTTTAT-3′.

The amplified 3′UTRs were then digested with EcoRV and XbaI enzymes and cloned in the same sites of pIS1-Eef25UTR-renilla vector. In addition, Eef1a1 3′UTR and Eif4a3 3′UTR were cloned into pIS1-Eef25UTR-TOPmut-renilla vector (Addgene 38236), that contains a mutated TOP motif in 5′UTR. All plasmids were sequence-verified.

Generation of Tetracycline (Tet) inducible cell lines

Tetracycline (Tet) inducible cell lines were generated as previously described (Sanna et al., 2015). Briefly, NSC-34 cells were primarily transduced with the pLentiCMV_TetR_Blast vector (716-1, Addgene). To establish an inducible cell line overexpressing the human HuD protein, NSC-34-Trex cells were infected with a lentiviral vector expressing His-HA tagged HuD. Alternatively, NSC-34-Trex cells were stably transfected with pSUPERIOR.neo+GFP plasmid containing the short hairpin sequence for Y3 or the empty vector as a negative control. In both cell lines, the inducible expression of the transgene (HuD or shRNA respectively) was induced by adding 2 μg/ml doxycycline (Clontech) to the culture medium.

Isolation of motor neuron compartments

Primary motor neurons were isolated from embryonic mouse spinal cord and cultured as previously reported (Conrad et al., 2011). To separate motor neuron axons from cell soma and dendrites, the use of coated filter insert (3.0 μm pores PET membrane) was adopted. After 5 days, the different cellular compartments were rapidly collected by scraping the both sides of PET membranes and RNA was extracted by Trizol (Life Technologies). To qualitatively analyze the separation of motor neuron axons from cell soma and dendritic tree, a small PET membrane piece was cut away, immersed in 4% PFA and processed for immunofluorescence.

Small interfering RNA (siRNAs) and cell transfections

For gene silencing of Y3, the following siRNA duplexes were used: AACUAAUUGAUCACAACCAGU for Y3 (Köhn et al., 2015) and AGGUAGUGUAAUCGCCUUG as non specific control (47% GC) (Eurofins Genomics); HuD was silenced by transfection of HuD siRNA (sc-37836, Santa Cruz) or control siRNA (sc-37007) from Santa Cruz (Kang et al., 2014). Cells were transfected with 100 nM of the indicated siRNAs for 24h by using Lipofectamine RNAiMAX Reagent (Life technologies).

HEK293 cells were transiently transfected with His-HA HuD plasmid or the His-HA empty vector as control. The transfections were performed using Lipofectamine 2000 (Life Technologies); 48h after transfections cells were harvested for the analysis.

For luciferase assay, HEK293 cells were transfected with His-HA HuD or His-HA empty vector (75ng). After 24 h, the cells were transfected with both the different renilla luciferase reporter vectors (50 ng) and Firefly luciferase (5 ng) for the normalization. The luciferase activity was measured after 24h using Dual-Glo Luciferase Assay System (Promega) following the manufacturer's instructions.

Motor neurons (2 DIV) were transfected by magnetofection using NeuroMag (OZ Bioscience) according to the manufacturer's protocol. At 5 DIV, neurons were fixed in 4% PFA and immunostained.

NSC-34 cell treatments

To inhibit mTORC1 pathway, NSC-34 cells were starved by serum depletion in DMEM medium without FBS for 8h or treated with Torin1 (500nM) for 1h. After the incubation time, the cells were collected for the following analysis. For the induction of cytoplasmic stress granules, NSC-34 cells were starved for 8h and then treated with 0.25 mM of sodium arsenite (Sigma-Aldrich). After 45 min, the cells were fixed und subjected to immunofluorescence analysis.

Cell Differentiation

NSC-34 cells were seeded onto collagen coated (50 ug/mL) 96-well microplate. The normal medium was exchanged 24h after seeding to differentiation medium containing 1:1 DMEM/F-12, 1% FBS, 1% modified Eagle's medium nonessential amino acids (NEAA), 1% P/S, 5 μM retinoic acid for seven days. HuD overexpression or Y3 short hairpin were induced by 2 ug/mL doxycycline. The differentiation medium was changed after three days. For PC12 cells, they were plated on collagen-coated plates and differentiated with 100 ng/ml NGF in Dulbecco's modified Eagle's medium supplemented with 1% horse serum. After 24h, the cells were transfected with HUD and Y3 vectors and maintained for 5 days in differentiation medium.

ESC neural conversion/neuronal differentiation procedure was performed as previously described (Ying et al., 2003). Briefly, ESCs were dissociated and plated onto 0.1% gelatin-coated tissue culture plastic dishes at a density of 1 × 104 cells per cm2 in N2B27 medium. Medium was completely renewed every 2 days. N2B27 medium was a 1:1 mixture composed of DMEM/F12 supplemented with N2 and Neurobasal medium supplemented with B27 (Thermo Fisher). After day 9, cell culture medium was shifted to diff-N2B27 composed of a 1:4 mixture of DMEM/F12 supplemented with N2 and Neurobasal medium supplemented with B27 (Thermo Fisher).

Immunofluorescence microscopy

Immunofluorescence of both motor neurons, NSC-34 cells and differentiating ESCs, was performed with the same protocol. After fixation in 4% PFA, cells were permeabilized in PBS + 0.1% Triton X-100 for 5 min and incubated in blocking solution (2% bovine serum albumin, 2% fetal bovine serum, 0.2% gelatin in PBS) for 30 min at RT. Primary antibodies were incubated for 2 hours at RT in blocking solution diluted 1:10 in PBS. The following primary antibodies were used: mouse anti-MAP2 1:300 (M4403, Sigma-Aldrich), rabbit anti-Tau 1:300 (314 002, SynapticSystem), anti-SMI32 (200 KDa neurofilament) 1:300 (Ab7795, Abcam), rabbit anti-MNX1 1:100 (ABN174, Millipore), mouse anti-HuD 1:200 (sc-28299, Santa Cruz), mouse anti-beta III Tubulin (T8578, Sigma

Aldrich), mouse anti-eEF1A1 (05235, Millipore), rabbit anti-eIF4A2 (31218, Abcam), rabbit anti-PABP 1:500 (Ab21060, Abcam), mouse anti-DCP1A 1:200 (Ab57654, Abcam), goat anti-TIA1 1:100 (sc-166247, Santa Cruz), mouse anti-Oct4 1:400 (sc-5279, Santa Cruz), mouse anti Nestin 1:400 (MAB353, Merck-Millipore), mouse anti beta3-Tubulin 1:1000 (G712A, Promega). The following secondary antibodies, diluted 1:800, were used: goat anti-rabbit Alexa Fluor 488 (A11008, Thermo Fisher Scientific), goat anti-rabbit Alexa Fluor 594 (A11012, Thermo Fisher Scientific), goat anti-mouse Alexa Fluor 488 (A11017, Thermo Fisher Scientific), goat anti-mouse Alexa Fluor 594 (A11020, Thermo Fisher Scientific), donkey anti-rabbit Alexa Fluor 488 (A21206, Thermo Fisher Scientific), donkey anti-goat Alexa Fluor 594 (Ab150136, Abcam). Nuclei were stained with DAPI. Images were acquired with Zeiss Observer Z.1 Microscope implemented with the Zeiss ApoTome device. The objective used for image acquisition was either PlanApo oil immersion lens 63x/1.4 or EC Plan-Neofluor 20x/0.5. Pictures were acquired using AxioVision imaging software package (Zeiss) and assembled with Adobe Photoshop 7.0. Images were not modified other than adjustments of levels, brightness and magnification.

Neurite outgrowth analysis

NSC-34 cells were fixed after seven days of differentiation and stained with Hoechst and mouse anti-Tubulin antibody (1:800; sc-53140, Santa Cruz). For HuD overexpressing cells, an additional immunostaining with a rabbit anti-HA antibody (1:600; A190-108A, Bethyl Laboratories) was performed. The following secondary antibodies, diluted 1:800, were then used: goat anti-rabbit Alexa Fluor 594 (A11012, Thermo Fisher Scientific), goat anti-mouse Alexa Fluor 488 (A11017, Thermo Fisher Scientific) and goat anti-mouse Alexa Fluor 594 (A11020, Thermo Fisher Scientific).

Neurite outgrowth was then analyzed on tubulin positive cells by High Content Screening System Operetta (PerkinElmer). Briefly, plates (96-well CellCarrier, PerkinElmer) were imaged and acquired in preselected fields with LWD 20x objective. For the feature extraction, the images were analyzed by Harmony software version 4.1 (PerkinElmer). Based on the Hoechst dye cell nuclei were identified. Starting from the cell body region, neurites were then detected in tubulin positive cells. The building block "Find Neurites" automatically calculated for each cell a set of neurite properties.

CRAC

The CRAC protocol was modified from the published one used in (Helwak et al., 2013). Trex-HuD NSC-34 and control Trex NSC-34 cells were seeded onto 150 mm plates (Nunc, Thermo Scientific). Cells were then induced for human HuD production with 10 μg/ml Tetracycline. 24 h post induction growing cells were UV crosslinked on ice with $\lambda = 254$ nm in Stratalinker 1800 (Stratagene). The cells were lysed and treated with DNase (Promega M610A). Cell lysates were incubated with HA agarose beads (26181 Pierce). Ribonucleoprotein complexes on HA beads were trimmed with 0.5 unit RNaseA+T1 mix (RNace-IT, Stratagene 400720-81) and HuD-RNA complexes were eluted. The eluate was incubated with Ni-NTA Agarose (Ni-NTA Superflow 50% suspension IBA 2-3206-010). RNAs bound to HuD were radiolabelled with 32P-γ-ATP and 3′ miRCat-33 linker ligation was performed. Then RNA ligase 1 and barcoded 5′ linker were added and the reaction mixture HuD-RNA complexes were eluted by incubation with NuPage-Eluition buffer. Protein-RNA complexes were resolved on a 4%–12% Bis-TrisNuPAGE gel (Life Technologies, NP0335) in NuPAGE SDS MOPS running buffer (Life Technologies, NP0001) and transferred to nitrocellulose membrane (GE Healthcare, AmershamHybond ECL). Air-dried membrane was exposed on film o.n. and the radioactive bands corresponding to the HuD complexes were cut out. RNA was extracted and reverse transcribed. cDNA was amplified and PCR products were precipitated, resuspended and separated on a 2.5% MetaPhoragarose (Lonza). After purification with Gel Extraction Kit with MinElute columns (QIAGEN) the samples were sequenced on Illumina HiSeq 2000 platform.

Polysome profiling

Polysomal profiling was performed according to previously described protocols (Bernabò et al., 2017). Briefly, the cells were treated with cycloheximide and then lysed in 300 μL of cold lysis buffer. The lysate was centrifuged at 4°C for 5min at 20.000 g to pellet cell debris. The cytoplasmic lysates loaded on a linear 15%–50% [w/v] sucrose gradient and centrifuged in a SW41Ti rotor (Beckman) for 1 h 40 min at 180.000 g at 4°C in a Beckman Optima Optima XPN-100 Ultracentrifuge. Fractions of 1 mL of volume, were then collected monitoring the absorbance at 254 nm with the UA-6 UV/VIS detector (Teledyne Isco).

Extraction of total and polysomal RNA

Sucrose fractions corresponding to polysomes and total RNA were pooled together and the RNA was processed by acid phenol–chloroform extraction. Alternatively, the mRNAs were isolated from single fractions along sucrose gradient as described in (Bernabò et al., 2017).

RT-qPCR analysis

The retrotranscription reaction was performed with 1 μg of polysomal or total RNA using the iScriptcDNA synthesis kit (Biorad) in accordance with the manufacturer's instructions. The obtained cDNA was used as template in aqPCR reaction with the KAPA SYBR FAST qPCR (Kapa Biosystem) and specific primers as reported in Table S3. qPCR were run in three biological and three technical replicates. The relative expression was calculated with the delta delta Ct method. Gapdh and Als2 were used as reference genes. The gene-specific Translation Efficiency (TE) was calculated as the ratio between the fold change at the polysomal level and the fold change at the total level of the gene of interest.

Library preparation for RNA-Seq and POL-Seq

Total and Polysomal RNA samples were converted to cDNA libraries according to the Illumina TruSeq Stranded mRNA Library Prep. The sequencing was performed on Illumina HiSeq 2500 platform.

Library preparation for TruSeq Targeted RNA Expression

The library was prepared using TruSeq Targeted RNA Expression following the manufacturer's instruction and the sequencing was performed on the MiSeq Illumina platform.

RNP immunoprecipitation and RNA pulldown

The HuD ribonucleoproteincomplex was isolated as previously described (Sanna et al., 2015). Immunoprecipitated and input samples were resuspended in Trizol reagent (Life Technologies) and RNA extraction was performed following manufacturer's instructions.

RNA pulldowns were essentially performed as previously described (Köhn et al., 2015). For synthesis of the RNA baits (Y1, Y3, Y4) T7-Polymerase mediated *in vitro* transcription was used.

SBP Pulldown

To pull down HuD and HuD-fragments inserts were cloned into the pCDNA-SBP-Flag vector. After transfection into NSC-34, cells were harvested after 48h. Cell pellets were lysed using BB (100 mMKCl, 10mM EDTA, 10 mM HEPES pH 7.4, 0.5% NP-40) and the supernatant was incubated with Streptavidin MyOne T1 beads (Life Technologies). Beads were then washed three times with BB and bound proteins were eluted by addition of BB+1% SDS and heating at 65°C. Eluates were then separated for RNA and protein preparations. Input and pulldown RNA was purified using Trizol (Sigma-Aldrich) and subjected to Northern Blot. Protein samples were subjected to Western Blot.

Northern and western blot

Northern Blot was essentially performed as previously described (Köhn et al., 2013, 2015).

For western blot analysis, NSC-34 and HEK293 cells were homogenized in RIPA lysis buffer (Sigma) following the manufacturer's instructions. The isolation of proteins along sucrose gradient were performed as described in (Bernabò et al., 2017). Protein lysates were resolved on SDS-PAGE and transferred to nitrocellulose membrane.

The following antibodies were used: mouse anti HuD (sc-28299, Santa Cruz), rabbit anti HA (A190-1081, Bethyl Laboratories), rabbit anti eIF4A1 (ab312-17, Abcam), rabbit anti-eIF4A2 (31218, Abcam), rabbit anti-eIF4A3 (homemade, generously provided from Prof. Macchi's lab), rabbit anti eEF1A1 (SAB2108050, Sigma), mouse anti Tubulin (sc-53140, Santa Cruz) and rabbit Anti-PABPC1 (SAB2101708, Sigma), rabbit anti Rpl26 (Ab59567, Abcam), rabbit anti S6 (2217, Cell Signaling Technology).

AlphaScreen assays

Recombinant HuD-His-HA proteins, expressed and purified from NSC-34 cells by Ni Sepharose beads (GE Healthcare), were tested in saturation binding conditions using biotinylated ARE RNA (5′-AUUAUUUAUUAUUUAUUUAUUAUUUA) or Y3 RNA probes and the AlphaScreen Hemagglutinin detection kit (Perkin Elmer) with an optimized protocol already described (D'Agostino et al., 2013). Equilibrium dissociation constants (Kd) were determined from nonlinear regression fits of the data according to a 1-site binding model in GraphPad Prism, version 5.0 (GraphPad Software, Inc., San Diego, CA). The RNA binding activity of recombinant HuD proteins in cell lysates was measured by reacting 50 nM of biotinylated probe upon determination of the assay specificity and hooking point.

Quantification of HuD and Y3 molecules

NSC-34 cells ($5x10^6$) were lysed using RIPA buffer and the protein concentration was determined using standard Bradford Protein assay (Sigma). Known amount of cell lysates and HuD recombinant protein (a generous gift of Dr. Paolo Struffi, University of Trento) were separated by 10% SDS-PAGE and transferred to nitrocellulose membrane. Samples were analyzed by western blotting using rabbit anti-HuD antibody (sc-28299, Santa Cruz) and the optical density (OD) of the protein bands were quantified by ImageJ. To estimate the number of HuD molecules NSC-34 cells, a standard curve was generated by plotting the known amounts of HuD recombinant protein (15, 25, 50, 75 ng) on the x axis, and their respective OD values on the y axis. This reference plot was used to inferred the amount of HuD protein in our NSC-34 lysate and calculate the amount for cell.

To estimate the number of Y3 molecules in NSC-34 cells, murine Y3 was synthesized by *in vitro* transcription. Total RNA was extracted from NSC-34 cells with Trizol (Sigma-Aldrich). The amount of RNA was normalized to the cell number and corrected for purification efficiencies. Then quantitative Northern Blots were performed to determine the amount of Y3 in NSC-34 total RNA by using *in vitro* transcribed Y3 as a standard. Finally, the amount of Y3 per NSC-34 cell could be determined.

AHA assay

De novo synthesized proteins were quantified using the Click-iT AHA Alexa Fluor Protein Synthesis HCS Assay (Molecular Probes, Life Technologies). In brief, NSC-34 cells were plated at density of 10,000 cells/well in 96-well plates for 24h. The cells were then induced to overexpress HuD (a) or silenced for HuD (b) or silenced for Y3 (c) or subjected to HuD overexpression and Y3 silencing

(d). After 48h, the cells were washed, incubated with L-azidohomoalanine (AHA) 50 μM for 1h and fixed. During AHA incorporation, control cells were treated with puromycin (100 ug/ml), a protein synthesis inhibitor, to evaluate background labeling. Click-chemistry reactions were sequentially performed according to the manufacturer's instructions and the relative AHA incorporation was then analyzed by high content imaging approach. To detect cell nuclei, the kit was multiplexed with Hoechst 33342. Plates (96-well CellCarrier, PerkinElmer) were imaged on the High Content Screening System Operetta (PerkinElmer). In each well, images were acquired in preselected fields with LWD 20x objective. For the feature extraction, the images were analyzed by Harmony software version 4.1 (PerkinElmer). Based on the Hoechst dye and Alexa 488 fluorescence intensity, cell nuclei and cell cytoplasm were identified respectively. To quantify nascent protein synthesis, the mean fluorescence intensity of Alexa Fluor 488 was quantified in the cytoplasm.

QUANTIFICATION AND STATISTICAL ANALYSIS

CRAC data analysis

Adaptor removal and collapse of duplicate reads (also with identical random barcode, marking PCR duplicates) were performed with hyb (https://github.com/gkudla/hyb). Reads were aligned to the mouse genome (GRCm38.p4) with Tophat (version 2.0.14), using the Gencode M6 transcript annotation as transcriptome guide. All programs were used with default settings unless otherwise specified.

In order to detect CRAC binding sites, we developed and implemented a dedicated computational methodology (MAPAS, standing for Mutation And PWM Assisted Search) that takes advantage of cross-linking induced mutations, consisting primarily in deletions in our experiment, in order to localize candidate binding sites. After the integration of replicates, to increase specificity, we penalized locations with aligned reads and deletions in control experiments (noise subtraction and removal). For each of the remaining locations, we calculated a combined p value based on a) the number of deletions, b) the number of aligned reads (coverage). P values were empirically calculated from the genome-wide experimental distributions of coverage and number of deletions. Coverage and deletion p values were combined with the Fisher method.

A pool of 753 sequences surrounding unique genomic locations with a combined p value < 0.05 were selected to build a PWM (Positional Weight Matrix), hence used as a "seed" matrix to score to all the other candidate binding sites. To create the seed PWM, we defined a region spanning seven nucleotides around the deletion site. This size choice is based on previous crystallographic studies resolving the structure of the RRM1 and RRM2 domains of HuD bound to canonical AU rich elements. PWM analysis was performed with functions implemented in the Biostrings R package. The seed PWM was used to score all deletion sites and select high-confidence HuD bound sites. A PWM score threshold was chosen, based on the 95 percentile of scores obtained from random heptamers. HuD "high confidence" binding sites were selected among those with i) PWM score > PWM score threshold, ii) number of HuD deletions > = 3 (at least one for replicate), iii) number of aligned reads > = 6 (at least two for replicate). This procedure identified 5153 high confidence HuD binding sites (Supplemental Information).

RNA-Seq and POL-Seq data analysis

For RNA-Seq and POL-Seq data of NSC-34 cells, after quality control (FastQC) reads generated from each sample were aligned to the mouse genome (GRCm38.p4) with STAR (version 2.5.3a,–quantMode TranscriptomeSAM GeneCounts), using the Gencode M6 transcript annotation as transcriptome guide. Normalization with the TMM method and identification of genes with altered TE upon HuD overexpression were performed with the edgeR package. The experiment was performed in biological duplicate.

TruSeq Targeted RNA Expression data analysis

TruSeq Targeted sequencing of 75 genes (including 70 HuD targets and 5 negative control genes) was performed to validate HuD RNA interactome (RIP-Seq in NSC-34 cells) and to monitor expression variations of HuD targets upon HuD overexpression (total RNA and polysomal RNA in NSC-34 cells), with the Illumina MiSeq platform. Raw counts were determined from the alignment of reads to targeted gene sequences. For HuD overexpression assay, normalization with the TMM method and identification of differentially expressed genes (p value < 0.05) were performed with the edgeR package. For the RIP-seq assay, negative control genes were used as housekeeping and data were normalized for the geometric mean of their expression values. Experiments were performed in biological triplicate.

Alternative polyadenylation data analysis

Transcriptome-wide alternative polyadenylation (APA) analysis upon HuD overexpression in NSC-34 cells was performed in biological triplicate by 3′end mRNA sequencing, using the Lexogen QuantSeq 3′ mRNA-Seq Library Prep Kit REV.

After quality control (FastQC), reads generated from each sample were aligned to the mouse genome (GRCm38.p4) with STAR (version 2.5.3a,–quantMode TranscriptomeSAM GeneCounts), using the Gencode M6 transcript annotation as transcriptome guide. Polyadenylation site usage (pAu) values were determined counting the number of reads starting within 10 nucleotides from known polyadenylation sites. Normalization and differential polyadenylation analysis were performed with the edgeR package.

Functional annotation enrichment analysis

Functional annotation enrichment analysis with Gene Ontology terms, KEGG and REACTOME pathways were performed using the clusterProfiler Bioconductor package.

Functional annotation enrichment analysis with lists of genes derived from experimental datasets was performed with the enrichR gene set libraries.

Statistical analysis

Unless stated otherwise all quantitative experiments were performed in triplicate and average with standard error of the mean (SEM) was reported in the corresponding figure legends. Analysis of data from sequencing and other experiments was carried out using programs described in the Key Resources Table and corresponding sections in STAR Methods.

DATA AND SOFTWARE AVAILABILITY

The accession number for the sequence files reported in this paper is NCBI GEO: GSE115490 (https://www.ncbi.nlm.nih.gov/geo/query/acc.cgi?acc=GSE115490). Raw image files are deposited on Mendeley Data (https://doi.org/10.17632/p34w7w78hy.1).

Molecular Cell, Volume *71*

Supplemental Information

HuD Is a Neural Translation Enhancer Acting on mTORC1-Responsive Genes and Counteracted by the Y3 Small Non-coding RNA

Toma Tebaldi, Paola Zuccotti, Daniele Peroni, Marcel Köhn, Lisa Gasperini, Valentina Potrich, Veronica Bonazza, Tatiana Dudnakova, Annalisa Rossi, Guido Sanguinetti, Luciano Conti, Paolo Macchi, Vito D'Agostino, Gabriella Viero, David Tollervey, Stefan Hüttelmaier, and Alessandro Quattrone

Supplementary Figures

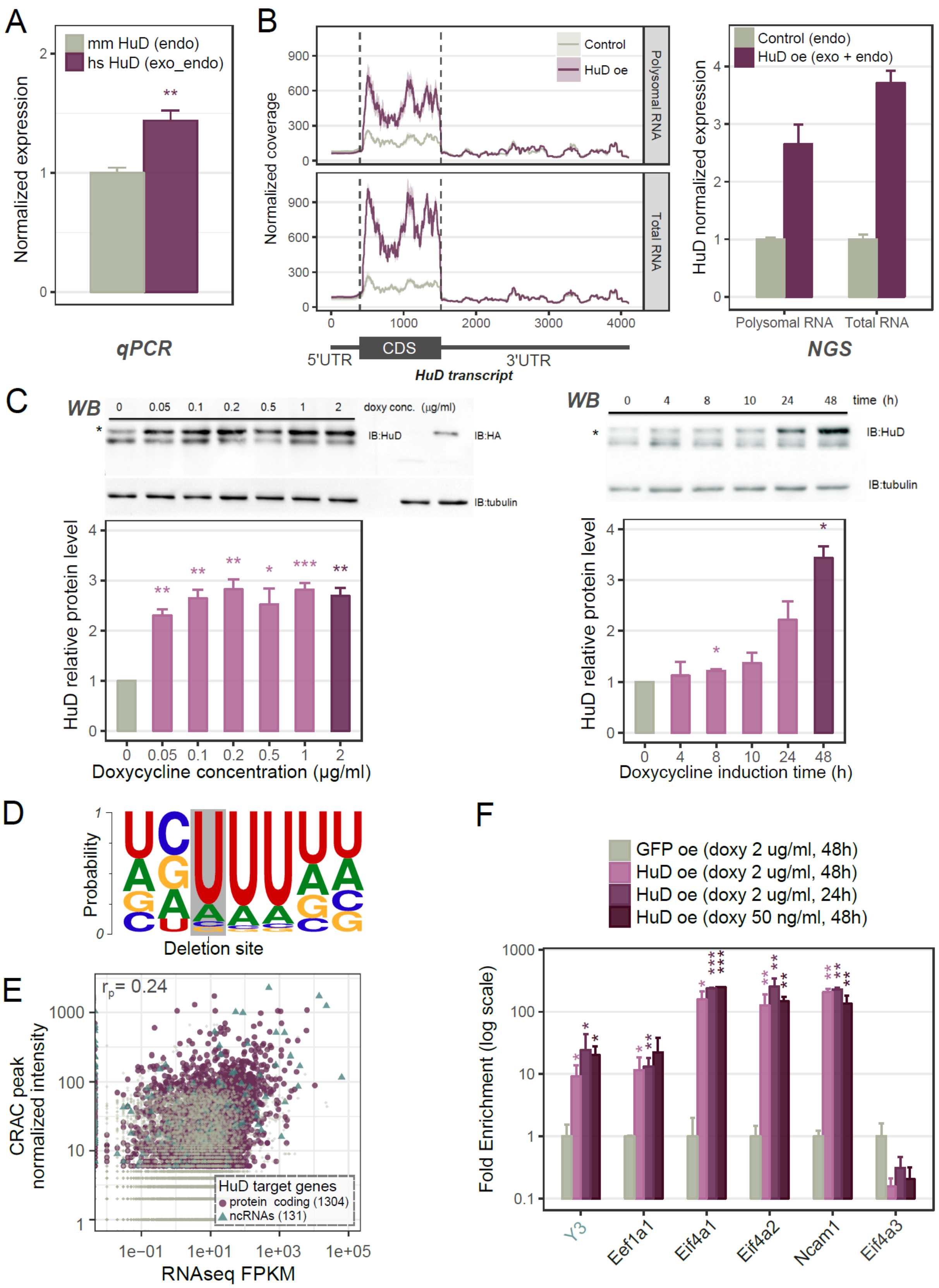

Figure S1. Related to Figure 1.

(A-C) Quantification of exogenous vs endogenous HuD expression in NSC-34 cells upon expression induction (doxy 2ug/ml, 48h).

(A) Quantitative RT-qPCR on human and murine HuD, monitoring endogenous and total HuD expression levels, respectively.

(B) Quantification of HuD exogenous/endogenous levels based on POL-seq and RNA-seq data, comparing the FPKM value of HuD 3'UTR, present only in the endogenous gene, and the FPKM of HuD coding sequence, present in both the endogenous and the exogenous genes.

(C) Quantification of HuD exogenous/endogenous protein expression levels by western blotting. NSC-34 cells were treated with different doses of doxycycline for 48h (upper panel) or with 2 µg/ml of doxycycline for different time points (lower panel). The levels of HuD and loading control α-tubulin were assessed; an HA antibody was used on the same gel to identify the exogenous HuD protein (*).

(D) Logo representation of HuD seed PWM (Position Weighted Matrix) determined from unique CRAC cross-linking locations with combined Fisher p-value < 0.05.

(E) Scatterplot displaying RNA-Seq transcript expression levels (FPKM) and normalized CRAC peak intensity values. The highest peak for each transcript is considered. HuD RNA targets are highlighted and annotated with their gene type.

(F) RIP analysis of HuD-interacting mRNAs encoding (Eef1a1, Eif4a1, Eif4a2 and Y3) under different doses (50 ng/ml or 2 µg/ml) or for different time (24 or 48h) of doxycycline treatment. Ncam1 and Eif4a3 were used a positive and negative control, respectively; data were normalized to Gapdh mRNA levels and expressed over control cells overexpressing His-HA-tagged GFP.

Data are represented as mean ± SEM. t-test $*p < 0.05$, $**p < 0.01$ and $***p < 0.001$.

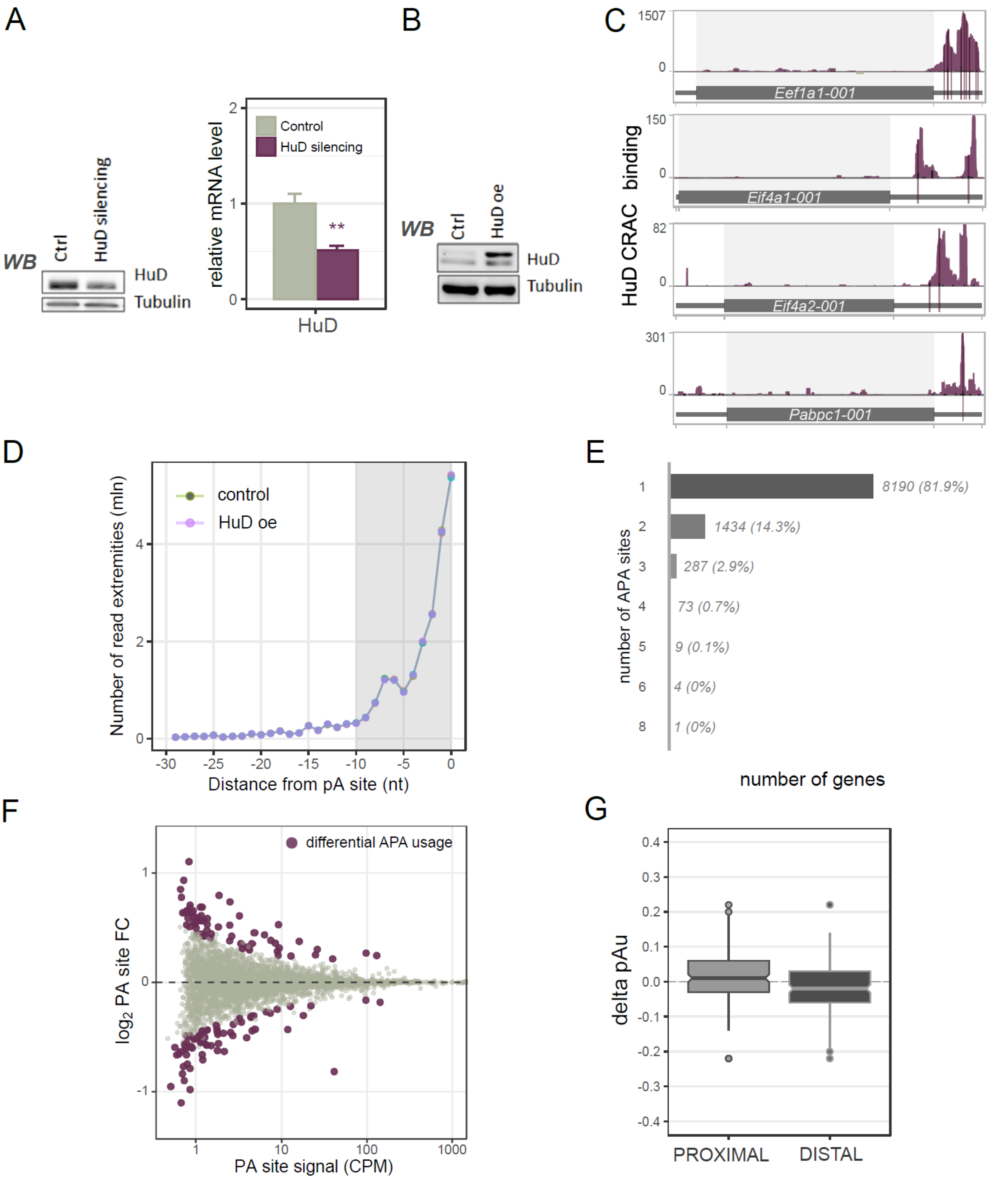

Figure S2. Related to Figure 2.

(A) HuD protein levels (left) and HuD mRNA levels (right) in NSC34 cells after transfection with Ctrl siRNA or HuD siRNA. Gapdh was used as reference gene for qPCR, while tubulin as a reference gene for western

blotting. Data were normalized to tubulin protein levels. Data are represented as mean ± SEM. t-test $**p < 0.01$.

(B) Western blot showing the overexpression of HuD protein upon doxycycline treatment (doxy 2ug/ml, 48h). Results were normalized to tubulin.

(C) Positional binding maps for three mTOR responsive HuD targets: Eef1a1, Eif4a1, Eif4a2 and Pabpc1.

(D-G): Transcriptome-wide alternative polyadenylation (APA) changes upon HuD overexpression in NSC-34 cells. Polyadenylation sites were identified and quantified by Lexogen 3'end mRNA sequencing.

(D) Polyadenylation site usage (pAu) values were determined counting the number of reads starting within 10 nucleotides from known polyadenylation sites.

(E) Distribution of the number of polyadenylation sites identified for each gene.

(F) Identification of differentially used polyadenylation sites upon HuD overexpression.

(G) Distribution of polyadenylation usage differences for proximal versus distal polyadenylation sites upon HuD overexpression.

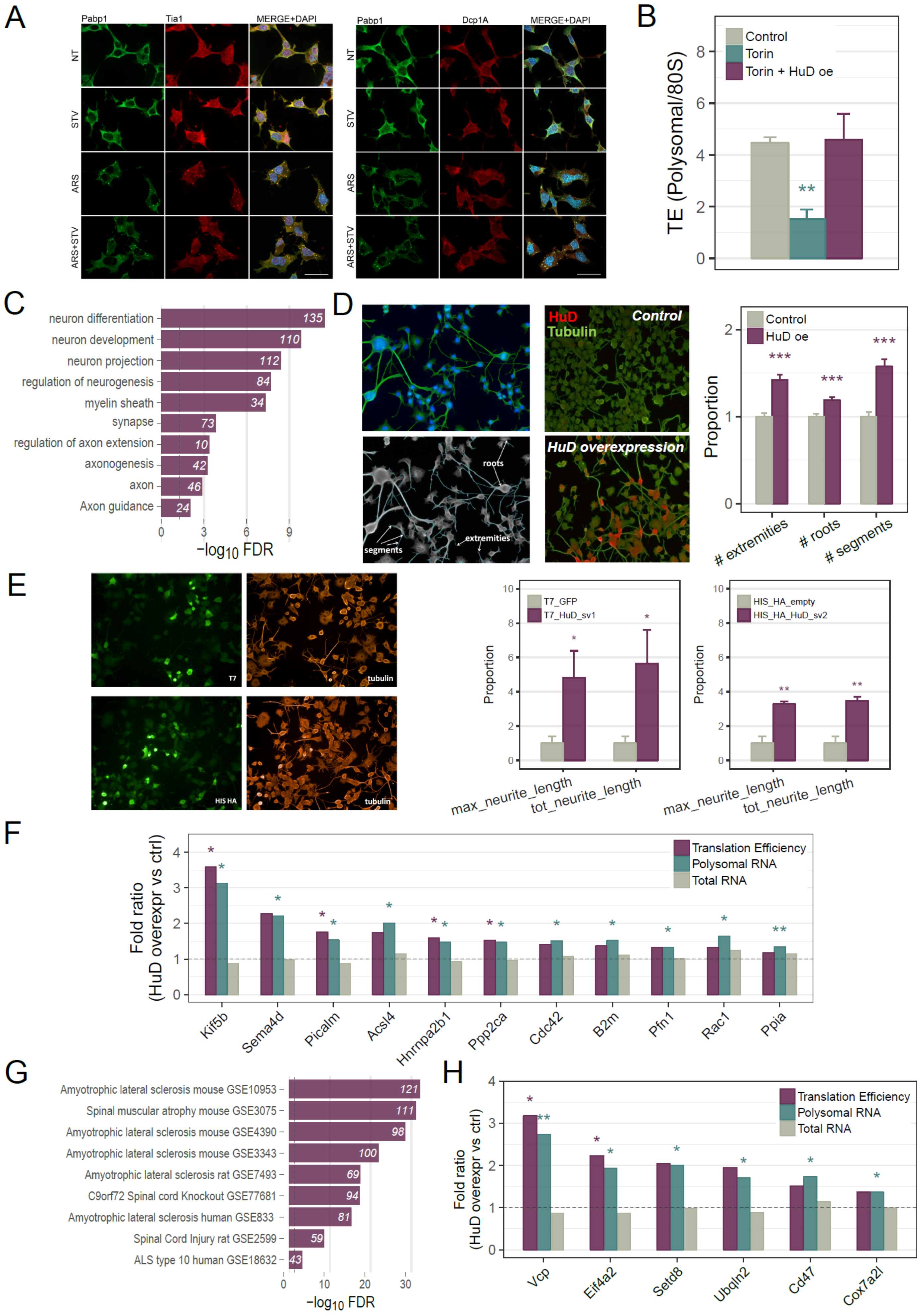
A
Pabp1
Tia1
MERGE+DAPI
Pabp1
Dcp1A
MERGE+DAPI
NT
STV
ARS
ARS+STV
B
Control
Torin
Torin + HuD oe
TE (Polysomal/80S)
**
C
neuron differentiation 135
neuron development 110
neuron projection 112
regulation of neurogenesis 84
myelin sheath 34
synapse 73
regulation of axon extension 10
axonogenesis 42
axon 46
Axon guidance 24
−log10 FDR
D
HuD
Tubulin
Control
HuD overexpression
roots
segments
extremities
Control
HuD oe
Proportion
***
extremities
roots
segments
E
T7
tubulin
HIS HA
tubulin
T7_GFP
T7_HuD_sv1
HIS_HA_empty
HIS_HA_HuD_sv2
Proportion
max_neurite_length
tot_neurite_length
F
Translation Efficiency
Polysomal RNA
Total RNA
Fold ratio (HuD overexpr vs ctrl)
Kif5b
Sema4d
Picalm
Acsl4
Hnrnpa2b1
Ppp2ca
Cdc42
B2m
Pfn1
Rac1
Ppia
G
Amyotrophic lateral sclerosis mouse GSE10953 121
Spinal muscular atrophy mouse GSE3075 111
Amyotrophic lateral sclerosis mouse GSE4390 98
Amyotrophic lateral sclerosis mouse GSE3343 100
Amyotrophic lateral sclerosis rat GSE7493 69
C9orf72 Spinal cord Knockout GSE77681 94
Amyotrophic lateral sclerosis human GSE833 81
Spinal Cord Injury rat GSE2599 59
ALS type 10 human GSE18632 43
−log10 FDR
H
Translation Efficiency
Polysomal RNA
Total RNA
Fold ratio (HuD overexpr vs ctrl)
Vcp
Eif4a2
Setd8
Ubqln2
Cd47
Cox7a2l

Figure S3. Related to Figure 3.

(A) Starvation in NSC-34 cells induces neither stress granule nor P-bodies formation. Left panel: NSC-34 were immunostained for Pabp1 (green) and Tia1 (red) to detect stress granules formation. Arsenite treatment was used as positive control for stress granules formation. The images show that starvation per se does not induce stress granules and does not increase arsenite-triggered stress granules. Right panel: NSC-34 were immunostained for Pabp1 (green) and Dcp1a (red) that is a P-bodies marker. Arsenite treatment was used as positive control for increasing P-bodies' size and number. The images show that starvation does not alter P-bodies in neither size nor number. The scale bars correspond to 40 μm.

(B) Measurement of global TE by sucrose gradient centrifugation as the ratio between the area under polysome peaks and the area under the 80S peak in the following conditions: control, Torin 1 treatment and Torin 1 treatment coupled with HuD overexpression. (data are represented as mean ± SEM. "Torin" was compared to "Control", and "Torin + HuDoe" was compared to "Torin" for testing statistical significance).

(C) Gene ontology neuronal-related enriched terms among HuD targets.

(D) Left: automated neurite segmentation performed with an Operetta HCS device (right). The cells were imaged on the High Content Screening System Operetta™ (PerkinElmer). The cytoplasm was immunostained with anti-tubulin antibody (green) to detect neuritic arborization, whereas the nuclei was identified by Hoechst staining (blue). The number of neurite segments, of extremities and of roots were quantified. Center: representative images of differentiated NSC-34 cells (control or overexpressing HA-tagged HuD) immunostained with anti-HA tag (red) and anti-tubulin antibodies (green); Right: neurite outgrowth quantification in differentiated NSC-34 cells overexpressing HA-tagged HuD compared to control.

(E) left: Representative images of PC12 cells overexpressing T7-HuD or HA-tagged HuD, immunostained with anti-HA or anti-T7 (green) and anti-tubulin antibodies (red); right: Maximum and total neurite length were analyzed using High content screening (HCS).

(F) Measurement of TE, polysomal RNA and total RNA variations on specific neuronal-related targets in HuD overexpressing cells compared to control. TE was calculated as the ratio between the polysomal and total RNA quantified by targeted sequencing and displayed in the plot in green and grey, respectively. HuD increases the TE of several neuronal-related targets, supporting neuronal differentiation.

(In panels B,C data are represented as mean ± SEM. t-test *$p < 0.05$, **$p < 0.01$ and ***$p < 0.001$).

(G) Enrichment of HuD targets among collections of genes with altered expression levels in motor neuron diseases (available in enrichR libraries).

(H) Measurement of TE, polysomal RNA and total RNA variations of HuD targets related to motor neuron diseases upon HuD overexpression in NSC-34 cells. TE was calculated as the ratio between the polysomal and total RNA quantified by targeted sequencing and displayed in the plot in green and grey, respectively.

(In panels B,D, E, F and H data are represented as mean ± SEM. t-test *$p < 0.05$, **$p < 0.01$ and ***$p < 0.001$).

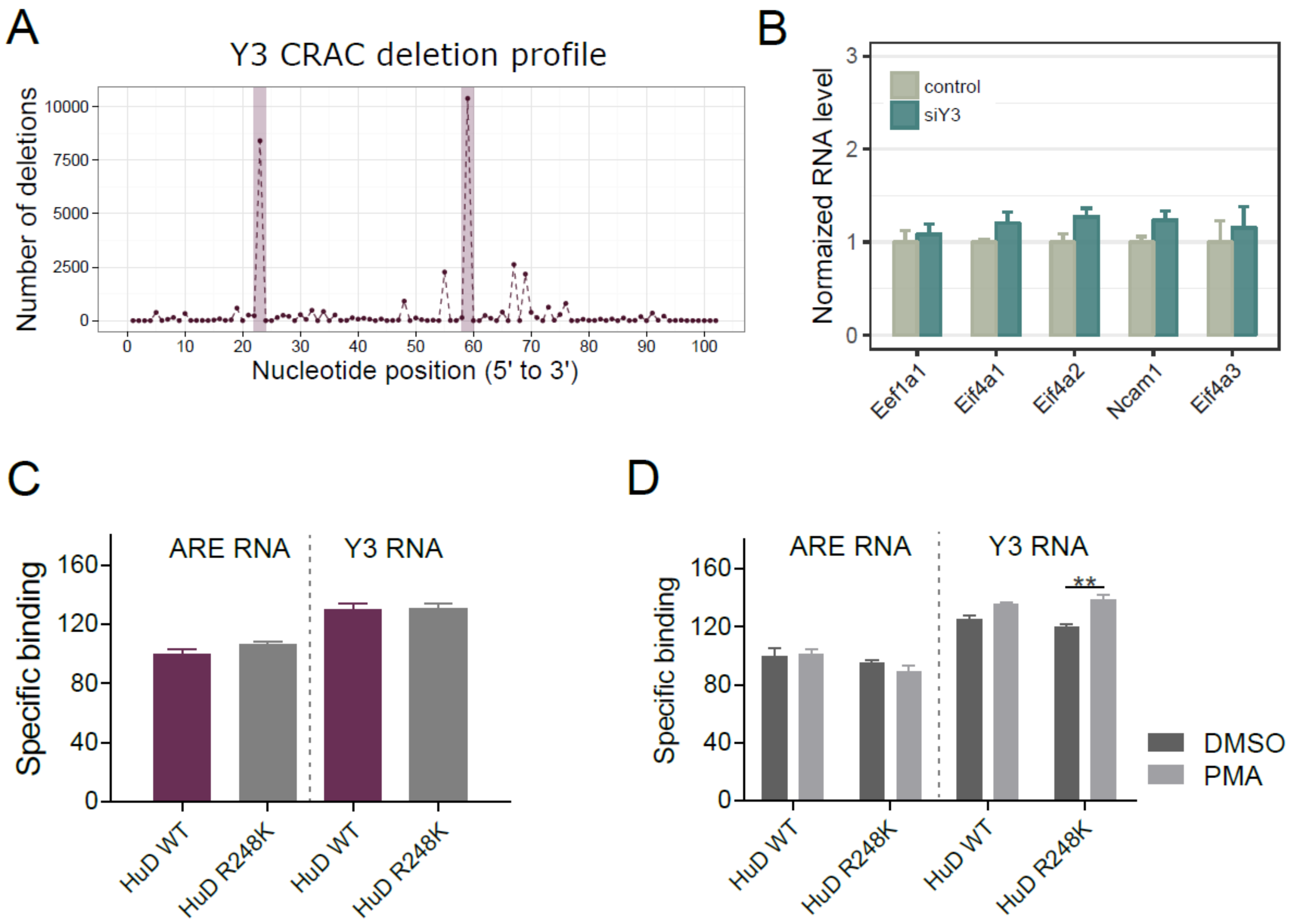

Figure S4. Related to figure 4.

(A) Y3 deletion profile, based on HuD CRAC. HuD binds to Y3 in two discrete locations, both positioned in polypirimidine tracts in the looped region outside the Y3 structural stem.

(B) mRNA levels of HuD target genes in control (scramble) or Y3 depleted cells (siY3) normalized to Gapdh. The graph is shown as mean ± SEM.

(C) Alpha screen assessing the specific binding of tagged-HuD and -HuD R248K proteins with ARE and Y3 RNA probes (C). Two independent experiments were performed at the hooking point with 50 nM of RNA probes.

(D) AlphaScreen assay measuring the specific binding of tagged-HuD and -HuD R248K proteins with ARE and Y3 RNA probes under PMA treatment for 45 min (D). Two independent experiments were performed at the hooking point with 50 nM of RNA probes.

(In panel D, data are represented as mean ± SEM. t-test **$p < 0.01$)

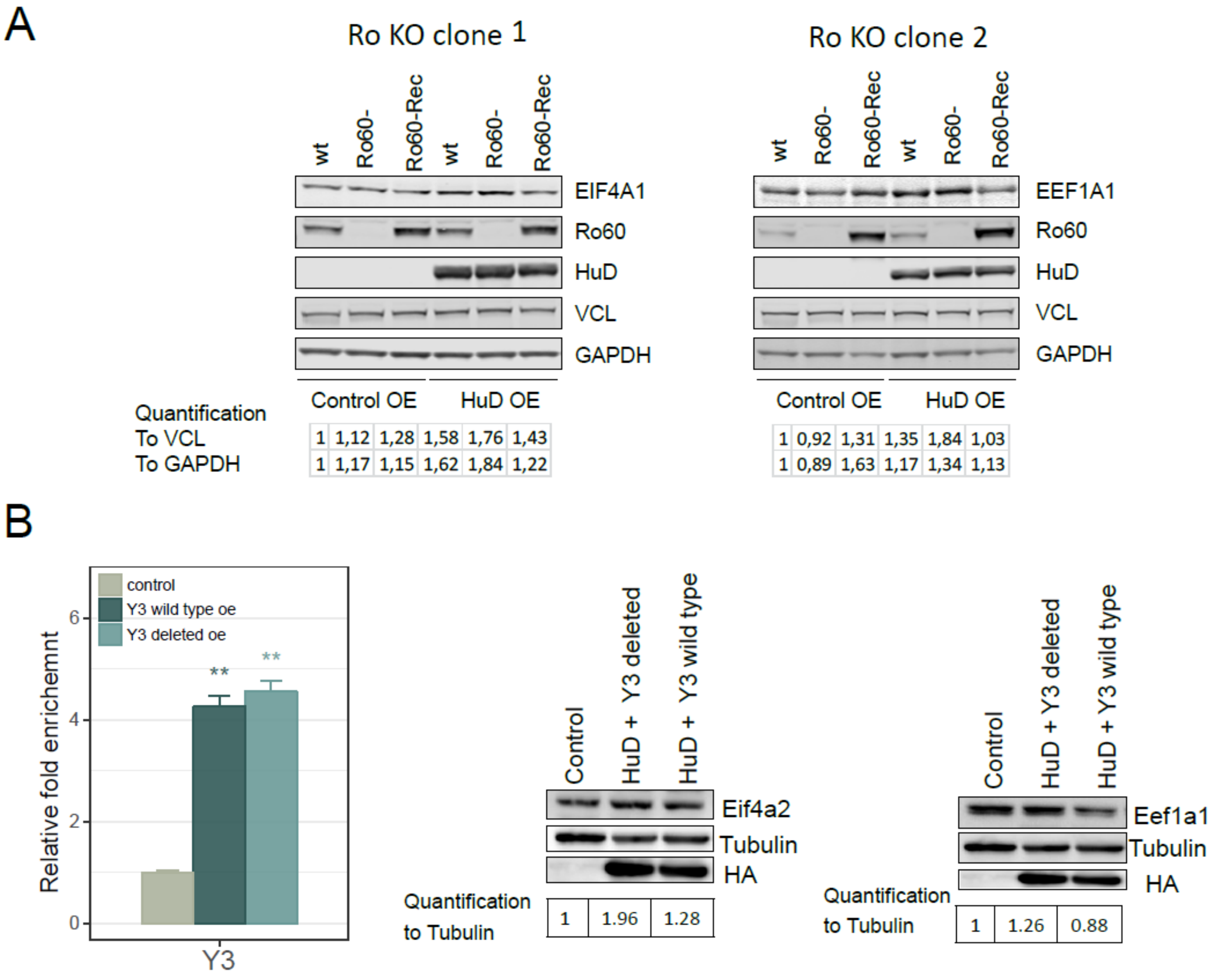

Figure S5. Related to Figure 5.

(A) The human ovarian cancer cell line ES2 was used to study the Ro60/Y RNA dependence of HuD activity on one of its target mRNAs. The set of wt cells, Ro60-knockout cells (Ro60-) and KO-cells with Ro60 recovery (Ro60-Rec) was either transfected with empty plasmid or HuD plasmid in both knockout clones. The HuD targets EIF4A1 and EEF1A1 were quantified by Western Blotting.

(B) HEK-293 cells were co-transfected with HIS-HA-HuD and deleted or wild type Y3. Left panel: qPCR assay confirmed the equal expression both Y3 wild type and Y3 mutant after transient transfection. 5S rRNA was used as housekeeping gene for normalization . Data are represented as mean ± SEM. t-test $**p < 0.01$. Right panel: the effect of the deletion was measured by western blotting on the HuD targets Eif4a2 and Eef1a1 by using α-Tubulin as a reference gene. HA antibody was used to verify HuD overexpression.

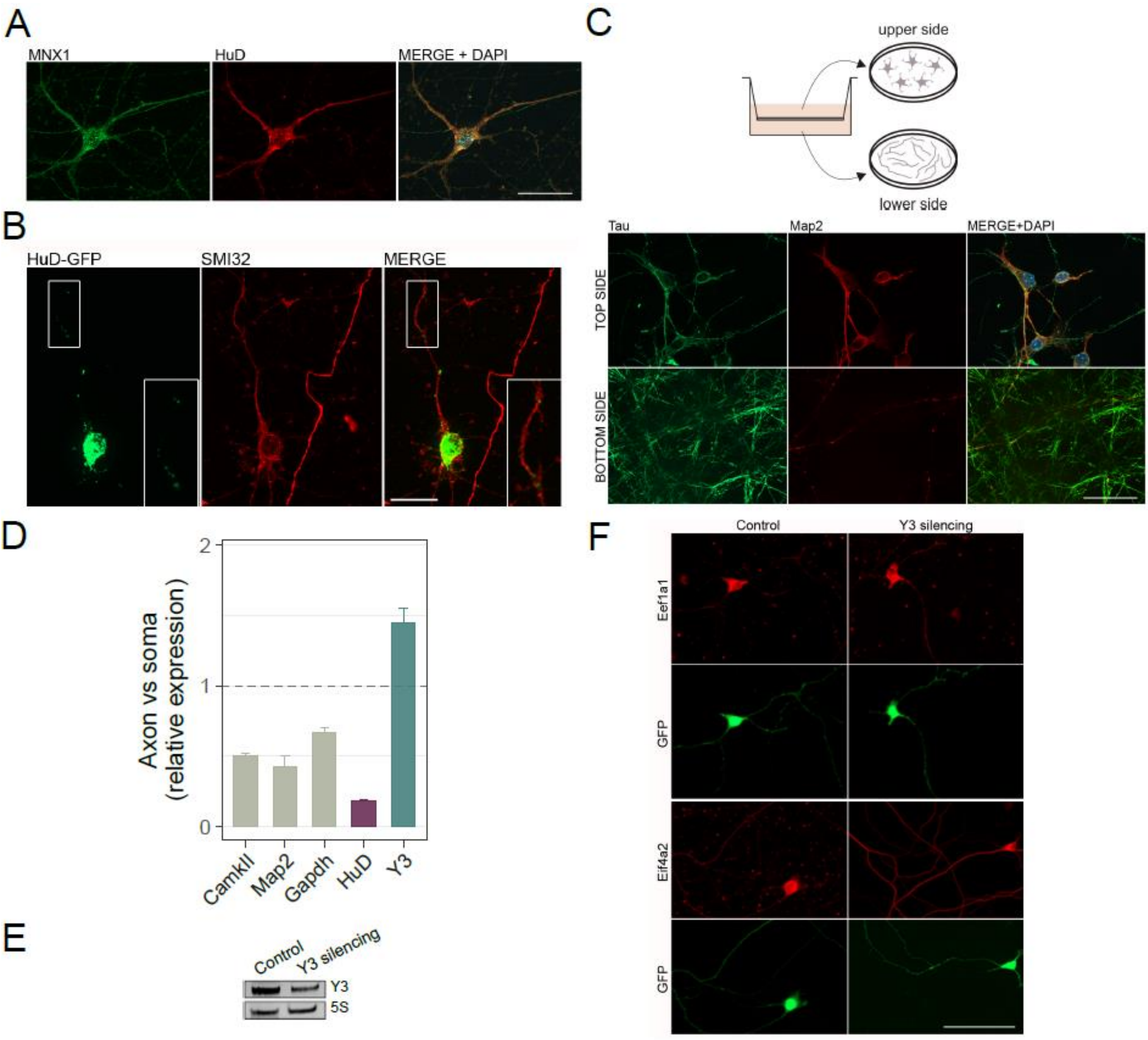

Figure S6. Related to Figure 5. HuD and Y3 are both expressed in motor neurons with a Y3 enrichment in axonal compartment.

(A) Primary motor neurons (5 DIV) immunostained for the motor neuronal marker Mnx1 (Hb9, green) and HuD (red). The scale bar corresponds to 40 µm.

(B) Motor neurons immunostained for the motor neuronal marker SMI32 after 48 hours transfection with pHuD-GFP vector. Both endogenous and transfected HuD shows a granular staining in the cell body, dendrites and along the axons. The scale bar corresponds to 20 µm.

(C) Top: Schematic overview of the separation system. Embryonic motor neurons were cultured in tissue culture inserts fitted with PET membrane with 3 µm pores. Neuron cell bodies remained on the upper membrane surface, whereas axons and few dendrites passed through membrane pores reaching the lower side of the membrane. Bottom: Immunofluorescent staining of motor neurons on PET membrane: neuronal

processes were marked with Tau (green), dendrites Map2 (red), cell nuclei with DAPI (blue). Images confirm that mainly axons (just few dendrites) cross the membrane and reaches the bottom side, with no contamination of cell bodies. On the contrary, cell bodies and dendrites were retained on the top side. The scale bar corresponds to 40 μm.

(D) RT-qPCR analysis of RNA extracted from motor neuron axons (scraped from the membrane bottom side) and compared with RNA extracted from cell bodies and dendrites (scraped from the membrane top side). Relative expression data show that *Y3* RNA is more present in the axons compared to cell soma and dendrites, while *HuD* and *Map2* are more localized to cell soma and dendritic tree.

(E) Assessment of the effective silencing of *Y3* RNA using the Y3-short hairpin vector.

(F) Immunostaining of Eef1a1 and Eif4a2 protein levels in primary motor neurons transfected with an shRNA construct directed against Y3 (sh_Y3) or a control vector (sh_Ctrl). GFP (green) identifies transfected cells that were subjected to quantification of either Eef1a1 or Eif4a2 signal (red). Acquisition and quantification was performed by Operetta HCS device. (n = >20 cells/condition).

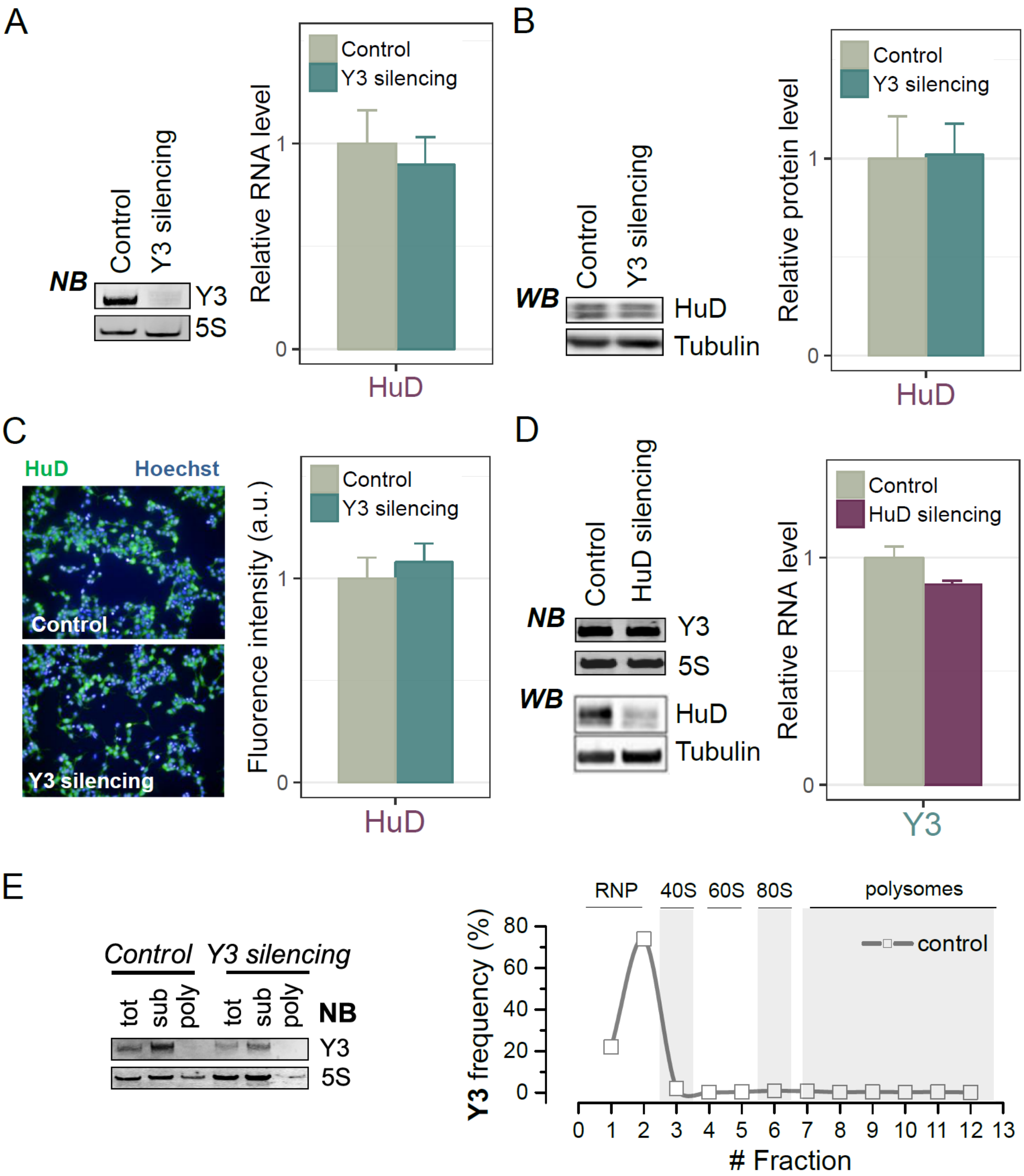

Figure S7. Related to Figure 6. HuD and Y3 do not mutually influence their expression.

(A) Left: Northern blot showing the efficient silencing of NSC-34 transfected with si-Y3 in comparison with control (scramble). 5S is used as housekeeping; right: level of HuD transcript quantified by RT-qPCR in si-Y3 NSC-34 cells compared to control. Rpl10a and Gapdh were used as reference.

(B) HuD protein levels quantified by Western Blot in control and Y3-siRNA cells.

(C) HuD protein levels quantified by immunofluorescence in control and Y3-siRNA cells.

(D) Y3 quantification by Northern blot in control and si-HuD cells. Western Blotting showing silencing of the HuD protein is also shown.

NB, WB and IF images are representative of at least three biological replicates.

(E) Left panel: Y3 quantification in total, subpolysomal and polysomal RNA fractions by Northern blot in control and Y3 depleted NSC-34 cells. Right panel: semi-quantitative analysis of Y3 relative transcript levels along the sucrose gradient fractions of control NSC-34 cells. Y3 is primarily localized in the RNP fractions.

Supplementary Tables

Target name	Forward primer	Reverse primer
**Als2**	catcatgttcaccaccttgaa	ctgtcaggcgccctagtta
**Bdnf**	caggaggaatttctgagtggcca	gcagaaggcctaagcaacttgac
**Eef1a1**	agtcacccgcaaagatgg	cgagttggtggtaggatacaatc
**Eef1b2**	acattgatctctttggatctgatg	gatggaagacttcgcaacaac
**Eif2a**	acttttagtaaggatgggacattgtt	tcccttgttagcgacattga
**Eif2s3x**	agccccgtctcattgtca	cacctcccttaaggtcatcg
**Eif4a1**	ggatcatgtctgcgagtcag	gctatccacaatctcgttcca
**Eif4a2**	cgatctacctaccaatcgtgaa	acctttcctcccaaatcgac
**Eif4a3**	cgaggaggtggacgtgac	tgctgaatcgctgaaggttt
**Eif4g2**	aaaggacagcttaatgcagatga	tgcactgggaggaatcatagt
**Eif4h**	tcaggaaaggtggacctgat	tcccatccacctctagattctc
**Gapdh**	ggaagggctcatgaccacag	acagtcttctgggtggcagtg
**HuD**	cagtgaacaacgtcaaggtcatc	ccttgcacttgttggtgttgaa
**Pabpc1**	ggtgccagacctcatcca	tgtgaggaagctggtctcatc
**Y3**	ggttggtccgagagtagtgg	aaaggctggtcaagtgaagc
**Rpl10a**	gaagaaggtgctgtgtttggc	tcggtcatcttcacgtggc
**Rpl32**	gggagcaacaagaaaaccaa	attgtggaccaggaacttgc
**Rps20**	tcctgactcaccgctgttc	ggcgtctttccggtatcttt
**Map2**	tgccacctgtttctctccac	tcttttgcttgctcgggatt
**Ncaml**	agggcaaggctgctttct	ccccatcatggtttggagt
**CamKII**	cacactcacaccacttcctt	gcattccatacaagagccaaac

Sample Name	Barcode	Read length after barcode removal
**CRAC_CTRL_1**	NNNCGCTTAGC	89
**CRAC_CTRL_2**	NNNTAAGC	92
**CRAC_CTRL_3**	NNNACTCAGC	90
**CRAC_HUD_1**	NNNGCGCAGC	90

**CRAC_HUD_2**	NNNATTAGC	91
**CRAC_HUD_3**	NNNGACTTAGC	89

Table S3. Related to STAR Methods.
Primer Sequences Used for Real-Time PCR and barcodes used for CRAC.
